# Structure-Based Discovery and Bioactivity Evaluation of Novel Aurora-A Kinase Inhibitors as Anticancer Agents via Docking-Based Comparative Intermolecular Contacts Analysis (dbCICA)

**DOI:** 10.3390/molecules25246003

**Published:** 2020-12-18

**Authors:** Majd S. Hijjawi, Reem Fawaz Abutayeh, Mutasem O. Taha

**Affiliations:** 1 Department of Pharmacology, Faculty of Medicine, The University of Jordan, Amman 11942, Jordan; mjd8120578@fgs.ju.edu.jo; 2 Department of Pharmaceutical Chemistry and Pharmacognosy, Faculty of Pharmacy, Applied Science Private University, Amman 11931, Jordan; r_abutayeh@asu.edu.jo; 3 Department of Pharmaceutical Sciences, Faculty of Pharmacy, University of Jordan, Amman 11942, Jordan

**Keywords:** Aurora-A kinase, aurora inhibitors, AURKA, structure-based drug design, docking-based comparative intermolecular contacts analysis, molecular docking, in-silico screening, MTT assay, PANC1, PC-3, T-47D, MDA-MB-231

## Abstract

Aurora-A kinase plays a central role in mitosis, where aberrant activation contributes to cancer by promoting cell cycle progression, genomic instability, epithelial-mesenchymal transition, and cancer stemness. Aurora-A kinase inhibitors have shown encouraging results in clinical trials but have not gained Food and Drug Administration (FDA) approval. An innovative computational workflow named Docking-based Comparative Intermolecular Contacts Analysis (dbCICA) was applied—aiming to identify novel Aurora-A kinase inhibitors—using seventy-nine reported Aurora-A kinase inhibitors to specify the best possible docking settings needed to fit into the active-site binding pocket of Aurora-A kinase crystal structure, in a process that only potent ligands contact critical binding-site spots, distinct from those occupied by less-active ligands. Optimal dbCICA models were transformed into two corresponding pharmacophores. The optimal one, in capturing active hits and discarding inactive ones, validated by receiver operating characteristic analysis, was used as a virtual in-silico search query for screening new molecules from the National Cancer Institute database. A fluorescence resonance energy transfer (FRET)-based assay was used to assess the activity of captured molecules and five promising Aurora-A kinase inhibitors were identified. The activity was next validated using a cell culture anti-proliferative assay (MTT) and revealed a most potent lead **85(NCI 14040)** molecule after 72 h of incubation, scoring IC_50_ values of 3.5–11.0 μM against PANC1 (pancreas), PC-3 (prostate), T-47D and MDA-MB-231 (breast)cancer cells, and showing favorable safety profiles (27.5 μM IC_50_ on fibroblasts). Our results provide new clues for further development of Aurora-A kinase inhibitors as anticancer molecules.

## 1. Introduction

Aurora kinases are a group of serine/threonine kinases fundamental for cell cycle control and mitosis. The first Aurora-kinase was discovered in Drosophila [1], where its mutations caused a failure of centrosome separation, leading to the formation of scattered monopolar spindles instead of bipolar ones; therefore, this kinase was named ‘‘Aurora’’ indicating resemblance to the northern lights phenomenon called Aurora borealis [1]. The homologues of Aurora have been identified in a family member, including Aurora-A, Aurora-B, and Aurora-C. The ATP-active binding site that is lined by twenty six residues, of which the three variants Leu215, Thr217, Arg220 are specific to and distinguish the Aurora-A kinase [2]. The localization and functions of the three homologues are largely distinct from one another [3]. Aurora-A kinase localizes to centrosomes and spindle poles from prophase to metaphase. Aurora-A is degraded at late mitosis or the early G1 stage [4,5,6].

Aurora-A kinase gene (AURKA) orchestrates the mitotic entry with other key players [7]. AURKA plays a crucial role in the performance of centrosome maturation and separation [8,9], accurate bipolar spindle assembly [10,11], and alignment of metaphase [5].

Exceptional attention for Aurora kinases has arisen since it was found that any defects in these kinases cause severe mitotic abnormality [3]. Of all the Aurora kinase family members, Aurora-A shows the strongest link to cancer. First, overexpression is oncogenic; second, the gene is located on chromosome 20q13, which is frequently amplified in multiple cancers, and this is usually accompanied by overexpression of Aurora-A, while the Aurora-B gene maps to chromosome 17p13 that is often deleted in a variety of cancers [4].

Amplification of the Aurora-A gene and its overexpression are observed in many malignancies, including breast, liver, pancreatic, bladder, and gastrointestinal cancers [12,13,14,15,16,17,18], and also in many hematological malignancies, multiple myeloma, and lymphoma [19,20]. Multiple roles are played by Aurora-A in regulating cancer development via promoting cell cycle progression [21,22]. AURKA overexpression enhances cell survival by suppressing autophagy through activating the mammalian target of rapamycin (mTOR) signaling [21,23,24], activating anti-apoptosis signaling, and/or cancer cell survival and chemoresistance by activating of PI3K/Akt/GSK3 [25,26] and NF-*κ*B signaling cascades [27,28]. AURKA overexpression induces the disruption of cell cycle checkpoints leading to aneuploidy and genomic instability [29,30]. AURKA overexpression contributes to epithelial-mesenchymal transition (EMT) by suppressing E-cadherin, β-catenin, and p53 [31]. Second, Aurora-A promotes EMT through activating several oncogenic signaling including AKT, MAPK, and focal adhesion kinase (FAK) [31,32]. Lastly, Aurora-A overexpression is observed in Cancer Stem Cell (CSC) enriched populations [12,17,33], denoting the central role of Aurora-A in promoting stem-like properties of cancer [17].

**Figure 1:**
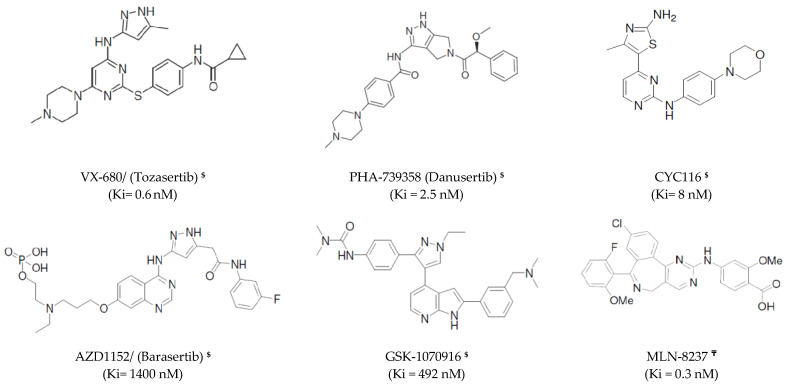
The chemical structures of Aurora-A kinase inhibitors studied via clinical trials. **^$^** Pan-selective to Aurora kinases A, B & C. **^₸^** Selective to Aurora- A kinase [39,40,41,42,43,44,45,46,47,48,49,50].

Most chemotherapeutic anticancer drugs used in the clinic today include agents that target the cell cycle to hinder the hyperproliferation status of the cancerous cells [34]. One class, or the cell cycle inhibitors based on their mode of action, are called antimitotics [35]. Historically, antimitotic agents were classified to two different classes. The first includes compounds that prevent microtubule assembly and disassembly (modulators of microtubule dynamics) [36]. The second one regulates mitosis by interacting with specific mitotic targets, such as mitotic kinesins or kinases [37]. The MT modulators, such as taxanes and various *Vinca* alkaloids, exhibit several undesirable side effects due to the important functions that microtubules play [36]. Thus, novel drug targets that inhibit the progression of mitosis, but independent on microtubules or non-tubulin binding, are highly required. Recent drug development focuses on improved novel drugs that induce mitosis-associated cell death or target mitotic kinesins and kinases, such as targeting Aurora-A [35,38]. More than a dozen small-molecule Aurora-A kinase inhibitors were tested in clinical trials (Figure 1) for different cancers in humans [39]. The most popular Aurora-A kinase inhibitors employed in clinical trials are summarized in Figure 1 with their bioactivity determined using a kinase binding assay [39,40,41,42,43,44,45,46,47,48,49,50]. Despite the encouraging anticancer effects in both preclinical and clinical studies, until now, no agent has been approved for use as anti-Aurora-A kinase to treat cancer. In this study, we aim to design new Aurora-A kinase inhibitors as promising anticancer drugs targeting the mitotic phase.

Computer-aided structure-based modeling is a drug discovery procedure that depends on the existence of a three-dimensional (3D)-structure of the enzyme to be targeted [51,52]. Docking is a fundamental element for structure-based drug design approaches that involve virtual fitting of ligands into the relevant binding site. Forcefield-based algorithms are employed by docking engines in order to calculate the interactions between bonded groups within the virtual ligand–protein complex, either attractive or repulsive [51,53,54]. Docking results in various conformational patterns exploring the best fitting one. Docking-based comparative intermolecular contacts analysis (dbCICA) is a new 3D-quantitative structure–activity relationship (QSAR) approach that was developed to build structure-based pharmacophores after verifying the best docking configurations [55,56]. Particular docking configurations are validated through defining a cluster of binding-site atoms in the binding-site to which potent ligands are fitted, while poorer or inactive ones evade contacting to docking ligands to the 3D structure for the protein [56].

Corresponding docking settings are assumed successful when they properly differentiate the molecules in the binding site, in an affinity-discriminating manner, explaining both the bioactivity variation among them and determining the critical interactions important for the affinity [57,58]. Moreover, successful dbCICA models give information about the vital amino-acids engaged in ligand-binding, which will be valuable in subsequent structure-based optimization [59]. Next, pharmacophoric model(s) are built based on the critical contact points determined by dbCICA modeling.

At last, pharmacophores are used for in silico database searching, aiming to find novel hits [51,56,57,60,61,62,63,64,65,66,67].

dbCICA was applied previously to discover new heat shock protein 90 inhibitors, new glycogen phosphorylase inhibitors, new fungal N-myristoyl transferase inhibitors, new CPK1 inhibitors, new FGFR1 inhibitors, new 3CLpro inhibitors, new Acetylcholinesterase inhibitors, new Gyrase b, new Flt3 and lately new JNK3 inhibitors [51,52,53,54,55,56,57,60,61,62,63,64,65,66,67].

In this research, seventy-nine Aurora-A Kinase inhibitors were docked into the binding pocket of the protein crystal-structure of Aurora-A Kinase, utilizing the LibDock docking engine [59]. The inhibitors were docked in two ionization states (unionized and ionized) and two binding-site hydration states (anhydrous and hydrous). High ranking docked poses produced by each docking setting were scored using seven scoring functions. Subsequently, dbCICA was used to gauge the success of different docking/scoring settings. Thereafter, the best dbCICA models were recruited as templates to create pharmacophores that were used later as a query to search, and to screen the National Cancer Institute (NCI) database. A fluorescence resonance energy transfer (FRET)-based Z′-LYTE^®^ kinase assay (Invitrogen, Carlsbad, CA, USA) [68,69] enzyme assay was used to assess the activity of captured molecules requested from the NCI. Five promising Aurora-A kinase inhibitors were identified. Their bioactivity was next evaluated using a FRET-based kinase assay and cell culture anti-proliferative assay (MTT), which revealed five promising molecules. The most potent lead inhibitor **85(NCI 14040)** scored half-maximal inhibitory concentration (IC_50_) values of 3.5 μM, 8.2 μM, 8.8 μM, and 11.0 μM against PANC1 (pancreas adenocarcinoma), PC-3 (prostate adenocarcinoma), T-47D (ductal breast carcinoma), and MDA-MB-231 (triple negative breast adenocarcinoma), respectively, and showed favorable safety profile (27.5 μM IC_50_ on fibroblasts). Our results provide new clues for further development of Aurora-A kinase inhibitors as anticancer molecules.

## 2. Results

### 2.1. Structure-Based Molecular Modeling

To understand the interactions associated in ligand binding into Aurora-A kinase, we implemented the innovative structure-based 3D-QSAR methodology; docking-based comparative intermolecular contacts analysis (dbCICA) [51,56,57,60,61,62,63,64,65,66,67] as a tool to study how the seventy-nine Aurora-A kinase inhibitors (Table S1 in Supplementary Material) bind into Aurora-A binding pocket. dbCICA modeling was used to create successful pharmacophore hypotheses that highlighted the critical binding interactions engaged in ligand-Aurora-A kinase binding. These successful pharmacophores were exploited as 3D search queries to screen the NCI database for novel anti-Aurora-A kinase molecules.

The collected seventy-nine inhibitors (Table S1 in Supplementary Material) [42,43,44,70,71,72] were docked in Aurora-A kinase binding pocket using LibDock [59,73] docking engine. In each case, four docking configurations were used: ionized ligands vs. unionized ligands, and hydrous binding site vs. anhydrous binding site. High ranking docking solutions were scored using seven different docking-scoring functions: Jain [74], LigScore1, and LigScore2 [75], PLP1, PLP2 [76], PMF and PMF04 [77,78]. The highest-ranking poses/conformers were aligned together to construct corresponding dbCICA models, according to each scoring function. 

The cycles of docking/scoring/dbCICA modeling were repeated to conduct all probable docking combinations, originating from using ionized ligands or un-ionized ligands, and the absence of crystallographically explicit water molecules, or its presence within the binding site. 

Intermolecular ligand-binding site contacts were discovered by applying two thresholds of distances: 2.5 Å and 3.5 Å. Thus, two corresponding contacts binary matrices were engendered for each docking solution (detailed in methods Section 4 Docking-Based Comparative Molecular Contacts Analysis (dbCICA)). Afterwards, a genetic algorithm (GA)-based search was employed to find the best combination of receptor–ligand intermolecular contacts, efficient in justifying the variations in bioactivity within training compounds. GA was exploited to visualize combinations of four to ten directly proportional intermolecular (positive) contacts followed by five or ten inversely proportional (negative) contacts. Interestingly, an intermolecular ligand binding site contact threshold of 3.5 Å yielded superior dbCICA models compared to their 2.5 Å counterparts; moreover, combining ten inversely proportional (negative) contacts yielded superior dbCICA models compared to combining five (negative) contacts.

Table 1 shows the contacts distance thresholds, number of negative and positive contacts, and the statistical criteria of the best dbCICA models based on the LibDock docking engine and multiple scoring functions. Clearly from Table 1, the best correlation statistics (particularly r^2^_5-fold_) were achieved for dbCICA models, based on two conditions: (i) docking ionized ligands into a hydrous binding site, using the PMF04 scoring function with five features and ten exclusion volumes, i.e., model (SB-1), and (ii) docking ionized ligands into a unhydrous binding site, using the Ligscore2 scoring function, with ten features and ten exclusion volumes i.e., model (SB-2). Table 2 shows critical Aurora-A kinase binding-site contact atoms and their weights as suggested by optimal dbCICA models, (SB-1) and (SB2).

### 2.2. Building Pharmacophores Based on Optimal dbCICA models

**Figure 2:**
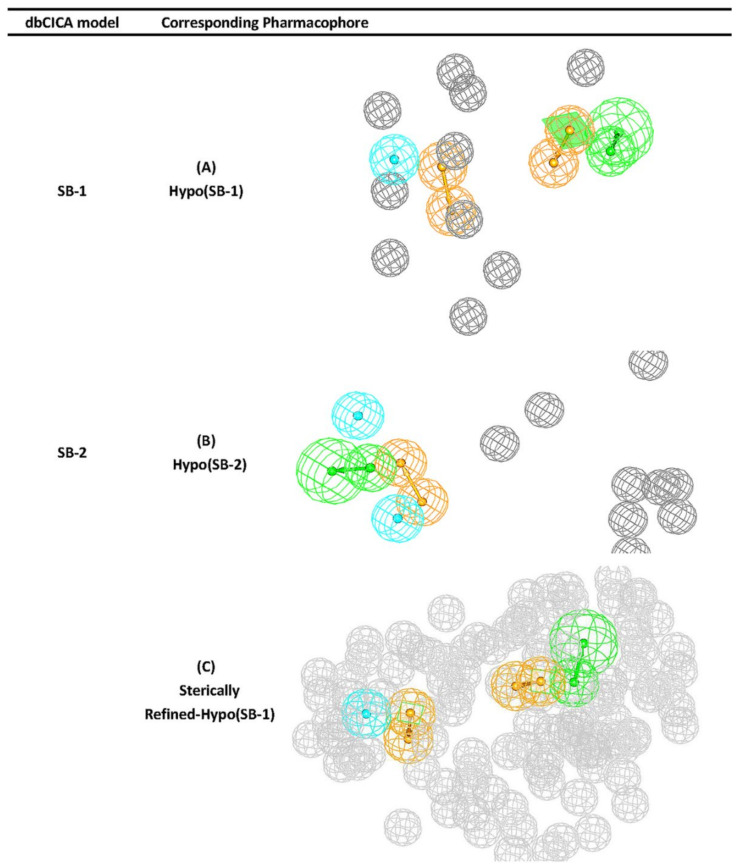
Successful dbCICA models, their corresponding pharmacophores, and the refined pharmacophore Hypo(SB-1) (**A**) pharmacophore extracted form successful SB-1 model that is based on docking ionized ligands into hydrous binding site using PMF04 scoring function with five features. (**B**) Hypo(SB-2) pharmacophore extracted form successful SB-2 model that is based on docking ionized ligands into unhydrous binding site using Ligscore2 scoring function with ten features and ten exclusion volumes. (**C**) Sterically-refined version of Hypo(SB-1) includes 93 exclusion spheres that are shown as grey spheres (1.2 Å tolerance radii). Light blue sphere represents Hbic (hydrophobic feature), orange-vectored spheres represent ring aromatic feature, green-vectored spheres represent hydrogen bond acceptor (HBA), and gray spheres represent exclusion region.

**Figure 3:**
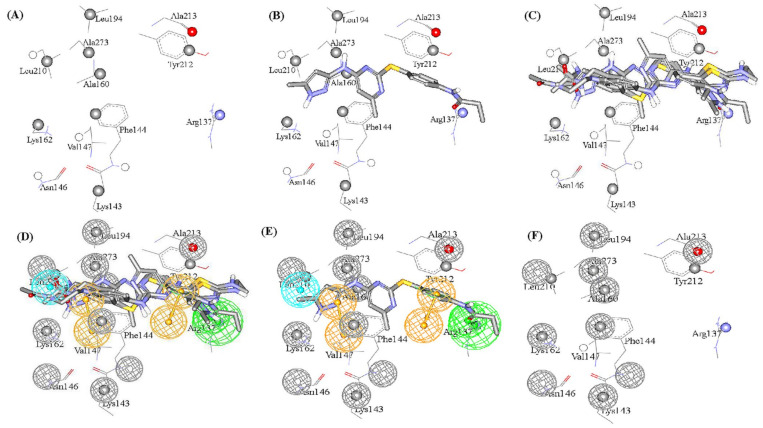
Steps for manual generation of binding model Hypo(SB-1) as guided by dbCICA model SB-1 (Table 1 and Table 2): (**A**) The binding site moieties selected by dbCICA model SB-1 with significant contact atoms shown as spheres. (**B**) The docked pose of well-behaved compound 52 (Ki = 5.1 nM) within the binding pocket. (**C**) The docked poses of the well-behaved compounds 50, 52, 71, and 77. (**D**) Manually placed pharmacophoric features onto chemical moieties common among docked well-behaved compounds 50, 52, 71, and 77. (**E**) The docked pose of 52 and how it relates to the proposed pharmacophoric features. (**F**) Exclusion spheres fitted against binding site atoms showing negative correlations with bioactivity (as emergent in dbCICA model SB-1).

Successful dbCICA models and their corresponding pharmacophore are summarized in Figure 2. As an example, for the complete stepwise process of pharmacophore building, dbCICA model (SB-1) translation into its corresponding pharmacophore within DiscoveryStudio 2.5.5 environment is demonstrated in Figure 3A–F (see Section 4 Manual Generation of Pharmacophores Corresponding to Successful dbCICA Models).

Initially, the binding pocket was annotated by rendering significant contact atoms in spherical forms according to the particular successful dbCICA model (Figure 3A). Subsequently, the docked poses of potent (Ki ≤ 5.1 nM) and the docked ligands that were well-behaved were left in the binding pocket (as in Figure 3B,C). Well-behaved ligands are those of least difference between fitted and experimental bioactivities, as determined by regression against their docking-based contact-summations in the specified dbCICA model. Thereafter, proper pharmacophoric features were positioned onto common aligned chemical functionalities of well-behaved docked potent ligands (Figure 3D). The pharmacophoric features were assigned in a way that hallmarks the interactions encoded by the critical contacts.

The conversion of the dbCICA model SB-1 (Table 1 and Table 2) into the corresponding pharmacophore model Hypo(SB-1) (Figure 2) is described herein (i.e., as an example, Figure 3A–F.

Figure 3 shows how pharmacophore Hypo(SB-1) was extracted from dbCICA model SB-1 (Table 1 and Table 2 and Figure 2). Appearance of significant contact at the NH of ARG137 and agreement among potent well-behaved docked ligands on placing nearby hydrogen-bond acceptor atoms (carbonyl oxygen of **52** and the heterocyclic *sp^2^* N of **50**, and **77**, **71**, Figure 3C,D) directed towards the guanidine of ARG137 suggested placing the HBA feature onto these ligand atoms, directed towards the guanidine of ARG137 (Figure 3D).

Likewise, emergence of significant positive contact at the isopropyl side chain of LEU210, combined with agreement among well-behaved potent ligands on placing hydrophobic groups nearby (e.g., methylpyrazole in **52**) suggested placing hydrophobic (Hbic) feature onto these groups, as in Figure 3D.

The emergence of significant positive contacts at TYR212 and PHE144, combined with agreement among potent well-behaved docked inhibitors to project aromatic molecular fragments, such as the pyrimidine, pyrazole, and phenyl ring in **52** and **50**, and pyrimidine, thiazole, and the phenyl ring in **71**, and pyrazine and phenyl rings in **77**, adjacent to these significant contacts, prompted us to place the aromatic ring pharmacophoric features (RingArom) at these positions (as in Figure 3D,E). Apparently, these features encode for stacking against the aromatic side chains of TYR212 and PHE144 (Figure 3C–E). Figure 2 shows the dbCICA-based pharmacophores, while Table 3 shows their 3D coordinates.

### 2.3. Evaluation via Receiver Operating Characteristic Analysis and HIPHOP Refinement Using Exclusion Spheres 

The resulting pharmacophores; Hypo(SB-1) and Hypo(SB-2) were evaluated using receiver operating characteristic (ROC) curve analysis to validate their capability of selectively capturing various potent Aurora-A kinase inhibitors from large lists of closely related experimentally-established Aurora-A kinase inhibitors extracted from the European Bioinformatics Institute database ChEMBL database (Testing Set, Table S2 in Supplementary Material), meaning that ROC analysis evaluates the ability of a pharmacophore to correctly discriminate between active compounds to be mapped and inactive compounds to be discarded. There are several successfulness criteria for ROC evaluation: (1) area under the curve (AUC) of the corresponding ROC curve; (2) sensitivity (SE) known as overall true positive rate (TPR) or truly active compound being captured, the (TPR) is calculated by dividing the true positive (TP) by the sum of true positives (TP), and false negatives (FN); (3) specificity (SP) known as overall true negative rate (TNR) or truly inactive compounds being discarded after proper identification, the (TNR) is calculated by dividing the true negative (TN) by the sum of true negatives (TN) and false positives (FP), and also (4) overall accuracy (ACC), and (5) overall false positives (FP). ROC performances are shown in Table 4. Hypo(SB-1) had superior/better specificity (SP) or (TNR) than Hypo(SB-2), though Hypo(SB-1) had weaker sensitivity (SE) or (TPR). It is known that the sensitivity (SE) gives information about active molecules that will be discarded, or the false negative compounds. The lower the false negative is, the higher the sensitivity, and the better the test in selecting active ones. Besides, Specificity (SP) gives information about inactive compounds that are wrongly mapped or captured, or the false positive compounds. The lower the false positive is, the higher the specificity, and the better the test in discarding inactive compounds. However, it is well-known that it is not possible to optimize both the specificity and the sensitivity simultaneously, and thus the ROC curve analysis providing a comprehensive image of the capability of a test to perform the distinction, considering all selection thresholds [79,80].

Therefore, the behavior of Hypo(SB-1) is not considered inferior in terms of sensitivity (SE). Looking back at (AUC) and (ACC) (Table 4), Hypo(SB-1) has higher (AUC) than Hypo(SB-2). It is known that the greater the AUC, the more effective the virtual screening workflow in discriminating active from inactive compounds. The most important dependable principle to assess which pharmacophore has better performance is concluded from ROC curves in Table 4. The optimal ROC curve is where active molecules are completely separated from inactive ones; the curve skyrockets vertically northwest to the upper left corner, significantly, starts at the point (0,1), which represent perfect classifier pharmacophore [79,80]. Accordingly, Hypo(SB-1) excel over Hypo(SB-2) because though Hypo(SB-2) starts at (0,1), however, it swings away from *y*-axis earlier than the Hypo(SB-1) does. Hypo(SB-1) starts at (0,1) and lays over *y*-axis, which means that it assigns the highest capturing values to several active molecules before it mistakes and captures an inactive molecule for the first time.

Based on ROC, results Hypo(SB-1) had better behavior over Hypo(SB-2) in terms of ROC-AUC, ACC, and TPR, and in order to enhance its ability to discard inactive compounds, or to improve its (TNR), it was decided to complement it with exclusion spheres by employing HIPHOP REFINE module of DiscoveryStudio 2.5.5. [81,82]. Although pharmacophore models help in understanding ligand–receptor interactions, they lack proper steric constrains crucial for identifying the binding pocket size. This problem renders pharmacophoric models sometimes promiscuous. Thus, it was decided to decorate our optimal dbCICA pharmacophore; Hypo(SB-1)**,** with exclusion spheres, using the HIPHOP REFINE module of DiscoveryStudio software suit [81,82]. Compounds listed in Table S2 in Supplementary Material, were utilized to decorate pharmacophore Hypo(SB-1) with exclusion spheres. This HIPHOP REFINE Testing Set (Table S2 in Supplementary Material) was chosen in a way that the weakly active-member bioactivities are explainable by steric clashes with the binding pocket. HIPHOP REFINE was configured to permit a maximum of 100 exclusion spheres to be added to pharmacophoric hypothesis. Pharmacophore Hypo(SB-1) was decorated with an additional 93 exclusion volumes.

Unsurprisingly, the sterically Refined-Hypo(SB-1) version of the pharmacophore (shown in Figure 2) illustrated improved ROC–AUC, ACC, and superior capability of discarding inactive compounds (TNR) in comparison to it is unrefined counterpart, and this is based on ROC analysis for Refined-Hypo(SB-1) (Table 4). Accordingly, it was decided to use the Refined-Hypo(SB-1) pharmacophore for in-silico search of the National Cancer Institution (NCI) database for new anti-Aurora-A kinase inhibitors.

### 2.4. In-Silico Screening and FRET Based Enzyme Inhibition Assay 

The fundamental use of pharmacophores models is the discovery of new chemical scaffolds demonstrating similar biological characteristics or scaffold hopping [56,57,58,59,60,61,62,63,64,65,83,84,85]. Therefore, Refined-Hypo (SB-1) pharmacophore (Figure 2) was used as a 3D query to search and screen the NCI list of compounds for new Aurora-A kinase inhibitors with novel chemotypes. The large number of added exclusion volumes on Refined-Hypo (SB-1) pharmacophore (i.e., 93), led to intensive data distinction, by which a huge number of inactive compounds were discarded upon in-silico screening, resulting in enriched probability that the remaining un-discarded hits are more likely to be active ones. The captured hits were filtered based on SMARTS SMILES Arbitrary Target Specification pattern) filtration to exclude reactive ligands [81] and based on Lipinski’s rule to ensure good pharmacokinetic properties [86]. Surviving hits were fitted against the Hypo(SB-1) and the lowest fitting hits were discarded leaving only 117 hits that were subsequently docked into Aurora-A kinase protein binding pocket (PDB code: 3w2c) employing the same docking/scoring settings of corresponding dbCICA models (SB-1) and (SB-2) (in Table 1). The crystallographic 3D coordinates of Aurora-A kinase protein were retrieved from the Protein Data Bank (PDB code: 3w2c).

The resulting docked poses (i.e., of hits) were analyzed to identify their binding critical contacts. The activity of each hit was predicted by using the sums of critical contacts as identified by the respective dbCICA model (Table 1 and Table 2) through substituting the sum of contacts with binding-site atoms in the corresponding dbCICA-regression equations [56,57,60,61,62,63,64,65,66,67].

**Figure 4:**
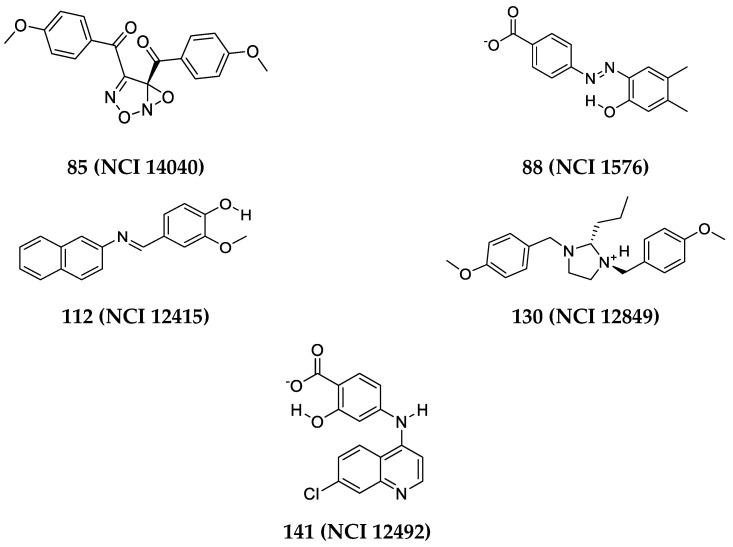
Chemical structures of the five hits that demonstrated inhibitory activity against Aurora-A kinase using Z′-LYTE^®^ assay (Invitrogen, Carlsbad, CA, USA) kinase inhibition assay with IC_50_ values summarized in Table 5. The identities and purities were validated by NMR and mass spectroscopy in (Figures S3–S38 in Supplementary Data).

Only 75 highest ranking hits were obtainable from the NCI to be further assessed via kinase inhibition assay. Table S3 and Figure S2 in Supplementary Material show the structures of the seventy-five captured hits (numbered 80–154) available from the NCI, their contact atoms summation, and their predicted bioactivities. These hits were bioassayed against Aurora-A kinase with a FRET-based enzyme inhibition assay conducted using Z′-LYTE^®^ kinase assay (Invitrogen, Carlsbad, CA, USA) [68,69]. The 75 hits were initially screened at 10 μM; results are summarized in Table S3, Supplementary Material. Five promising hits have shown anti-Aurora-A Kinase inhibition percentages exceeding 51% at 10 μM as detailed in Table 5. Inhibition percentages at 10 μM were equal to 86% by hit **130 (NCI 12849)**, 75% by **88 (NCI 1576)**, 56% by **85 (NCI 14040)**, 55% by **112 (NCI 12415)**, and 51% by **141 (NCI 12492)**. Chemical structures of the five hits are shown in Figure 4.

**Figure 5:**
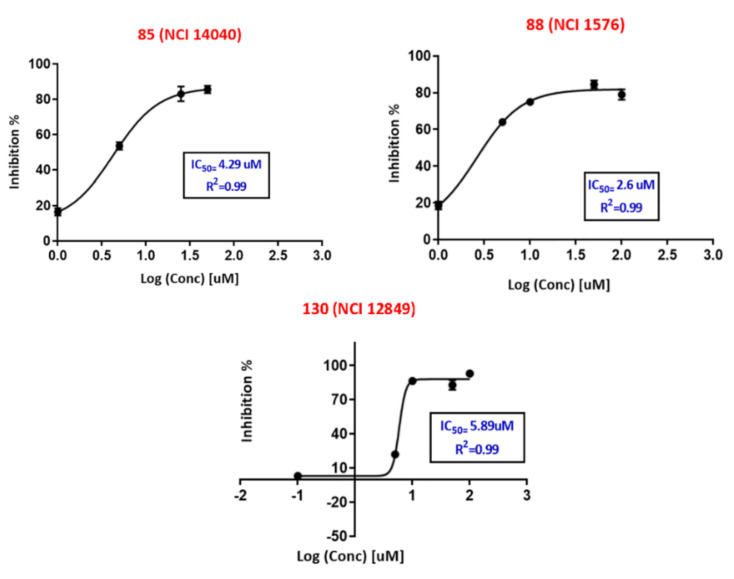
Dose-response curves for the three potent hits as anti-Aurora-A kinase using Z′-LYTE^®^ kinase assay (Invitrogen, Carlsbad, CA, USA) showing their IC_50_ values in Table 5.

Three of these promising hits (inhibition % at 10 μM above 56%) were next tested at several concentrations range (0.1–100 μM) to determine their IC_50_ values by using nonlinear regression to plot log (concentration) vs. inhibition % curves (shown in Figure 5) using GraphPad Prism software. Hits **88 (NCI 1576)** and **85 (NCI 14040)** demonstrated highest activity with IC_50_ values of 2.6 μM and 4.3 μM, respectively, and similar hill slope coefficient ≈1.7. Whereas hit **130 (NCI 12849)** IC_50_ was 5.9 μM with hill slope of 7.6.

The five promising hits codes, their contact atoms summation, their predicted bioactivities, and their inhibitory IC_50_ values are summarized Table 5 (other weakly active hits are presented in Table S3 and Figure S2 in Supplementary Material).

**Figure 6:**
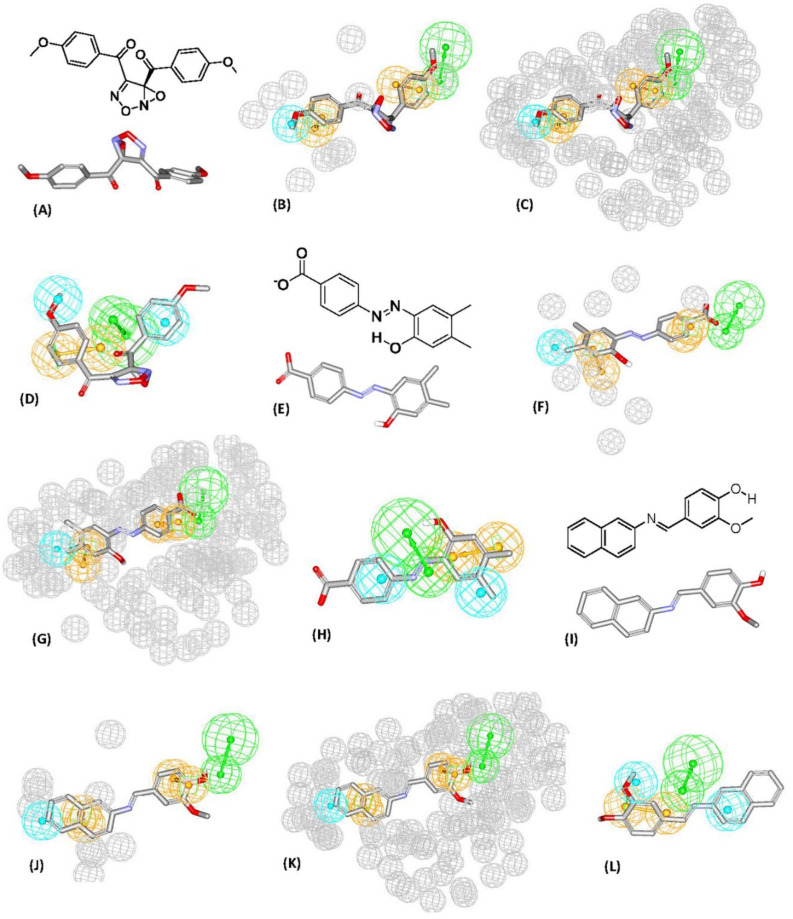

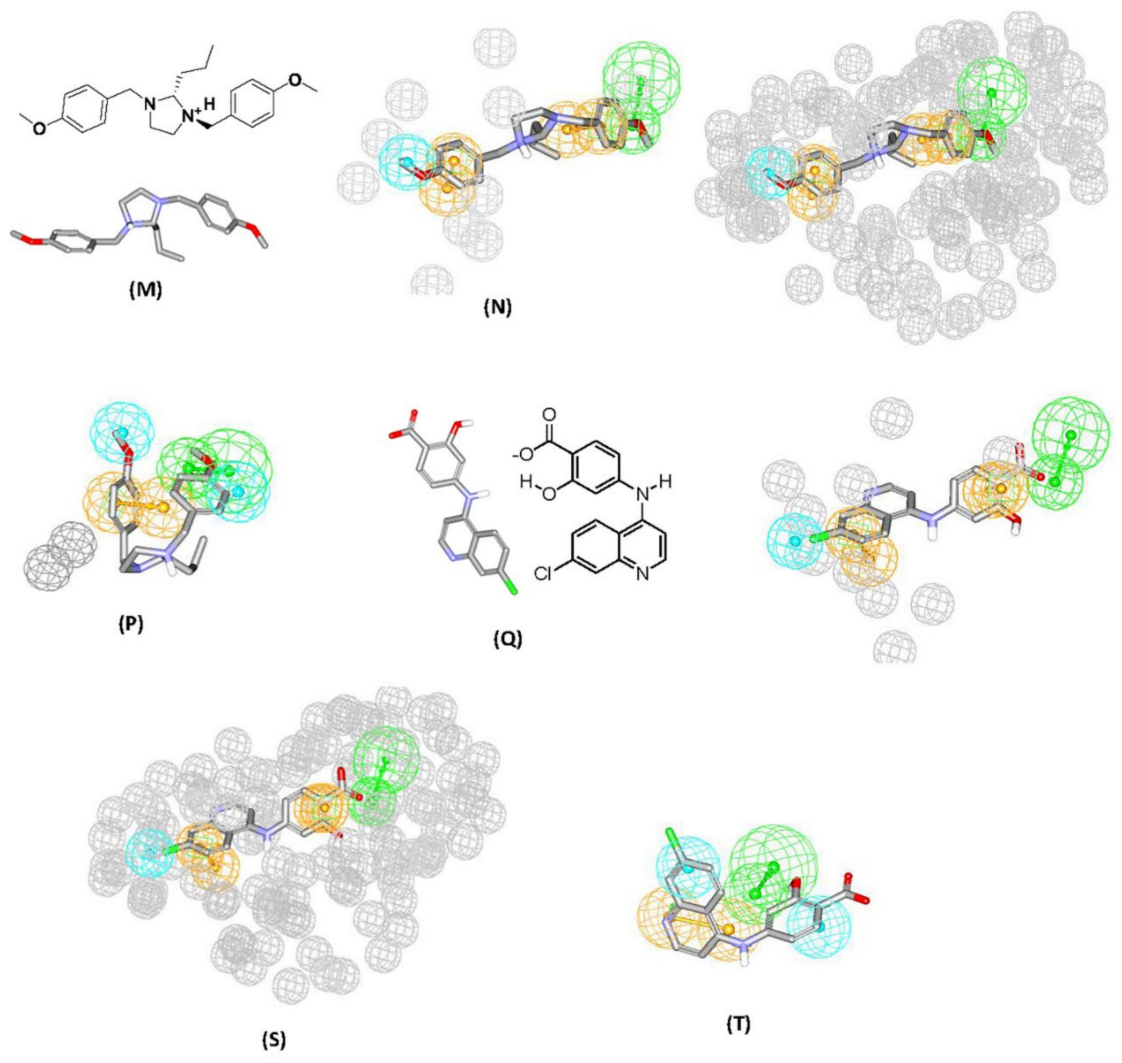
(**A**) Chemical structure of hit **85** (NCI 14040), (B) Hit **85** (NCI 14040) fitted to Hypo(SB-1) pharmacophore before refinement. (**C**) Hit **85** (NCI 14040) fitted to sterically Refined-Hypo(SB-1) pharmacophore. (**D**) Hit 85 (NCI 14040) fitted to the Hypo(SB-2) pharmacophore, (**E**) Chemical structure of **88 (NCI 1576)**, (F) Hit **88 (NCI 1576)** fitted to Hypo(SB-1) pharmacophore before refinement, (**G**) Hit **88(NCI 1576)** fitted to sterically Refined-Hypo(SB-1) pharmacophore. (**H**) Hit **88(NCI 1576)** fitted to the Hypo(SB-2) pharmacophore. (**I**) Chemical structure of **112 (NCI 12415)**, (J) Hit **112 (NCI 12415)** fitted to Hypo(SB-1) pharmacophore before refinement. (**K**) Hit **112 (NCI 12415)** fitted to sterically Refined-Hypo(SB-1) pharmacophore. (**L**) Hit **112 (NCI 12415)** fitted to the Hypo(SB-2) pharmacophore. (**M**) Chemical structure of hit **130 (NCI 12849)**, (N) Hit **130 (NCI 12849)** fitted to Hypo(SB-1) pharmacophore before refinement. (**O**) Hit **130 (NCI 12849)** fitted to sterically Refined-Hypo(SB-1) pharmacophore. (**P**) Hit **130 (NCI 12849)** fitted to the Hypo(SB-2) pharmacophore. (**Q**) Chemical structure of hit **141 (NCI 12492)**. (R) Hit **141 (NCI 12492)** fitted to Hypo(SB-1) pharmacophore before refinement. (**S**) Hit **141 (NCI 12492)** fitted to sterically Refined-Hypo(SB-1) pharmacophore. (**T**) Hit **141 (NCI 12492)** fitted to the Hypo(SB-2) pharmacophore.

Figure 4 shows the dose-response curves of the three most potent hits. Evidently, the dose-response curves of hits **88 (NCI 1576)** and **85 (NCI 14040)** show reasonable Hill slopes/coefficient values (<2) [87,88] and excellent R^2^ values, which strongly suggest their authenticity (i.e., non-promiscuous) in binding to the protein kinase. However, **130 (NCI 12849)** show steep dose-response curve, and Hill slope coefficient of 7.6 indicate additional non-specific enzymatic mechanisms of inhibition. Figure 6A–T demonstrates how these hits fit or map to the optimal Hypo(SB-1) before and after refinement (binding to sterically refined (SB-1)), and to Hypo(SB-2). 

Nuclear magnetic resonance (NMR) and mass spectrometry were used to validate the chemical structures and purities of those hits (Figures S3–S38 in Supplementary Data).

### 2.5. Cell Culture Anti-Proliferation Assay: In Vitro Evaluation

The five potent hits showing anti-Aurora-A Kinase inhibition above 51% at 10 μM using FRET-based Enzyme Inhibition Assay (Table 5) were further examined using MTT cytotoxicity assay against various cancer cells. The standard Aurora-A kinase inhibitor Tozasertib/(VX-680) [41] was used as a positive control. Tozasertib/(VX-680) is a pyrimidine derivative discovered in 2004 with high-affinity to Aurora-A Kinase and its inhibitory constant or Ki value for Aurora-A is 0.6 nM [40,41] (Figure 1). In-vivo, Tozasertib/(VX-680) reduced the tumor size by 22–98% in xenograft models of various cancer types [40,41,89]. Tozasertib is the first Aurora kinase inhibitor to be used in patients; however, its clinical trials were terminated in 2007 because it induced cardiovascular complications in one patient [17,39,40,41,90,91].

In the present research, the cytotoxicity of the five compounds was initially screened against cancer cell-lines including MDA-MB-231, PANC1, and T-47D at two preliminary concentrations: 50μM and 25μM. Cytotoxicity MTT assay results are detailed in (Table 6). Next, the compounds that produced cell death above 50% at 50μM were further examined to determine their (IC_50_) values. For MDA-MB-231 breast cancer cells, three hits **88 (NCI 1576)**, **130 (NCI 12849),** and **141 (NCI 12492)** all produced an IC_50_ inhibitory profile above 50 μM, accordingly, they were not investigated for further specific IC_50_ determination. Two compounds demonstrated anticancer bioactivity against MDA-MB-231 scoring IC_50_ values of 11.0 μM and 17.9 μM for hits **85 (NCI 14040)** and **112 (NCI 12415)**, respectively. Tozasertib/(VX-680), exhibited 7.7 μM IC_50_ against MDA-MB-231 cells, proposing that the two bioactive hits **85 (NCI 14040)** and **112 (NCI 12415)** produce lower anticancer efficacy against MDA-MB-231 than the standard positive control inhibitor; (VX-680).

Exploring the cytotoxicity results against PANC1 pancreatic cancer cells and T-47D breast cancer cells, four compounds demonstrated an IC_50_ inhibitory profile above 50 μM except hit **85 (NCI 14040)** that produced an IC_50_ value of 8.8 μM against T-47D cells, and a superior efficacy on PANC1 cells among all the tested compounds, and against all the examined cell-lines in this research with an IC_50_ of 3.54 μM. Interestingly, **Tozasertib/(VX-680)** IC_50_ values against PANC1 and T-47D cells were 7.6 μM and 17.9 μM, respectively, implying that hit **85 (NCI 14040)** possesses higher anti-proliferative activity as an Aurora-A kinase inhibitor than does the standard control; **Tozasertib/(VX-680).** Lastly, it was decided to test the hits showing activity against previous cell-lines on prostate cancer cells (i.e., PC-3). The IC_50_ of the potent hit **85 (NCI 14040)** was 8.2 μM showing superior efficacy over the (VX-680) standard inhibitor which produced an IC_50_ of 21.4 μM against PC-3 cell-line. Hit **112 (NCI 12415)** produced an IC_50_ value above 50 μM against PC-3 cell-line. 

Finally, to investigate the safety index of the compounds with specified IC_50_ values, these compounds were examined using MTT assay against Fibroblasts to determine their cytotoxic effect against non-cancerous cells. A compound that demonstrates a higher IC_50_ value against normal non-cancerous cells than the IC_50_ it produces against cancer cells, demonstrates favorable safety profile. The IC_50_ value of hit **85 (NCI 14040)** was 27.5 μM against fibroblasts, which is a concertation that is higher than all the IC_50_ values it produced against PANC1, PC-3, T-47D, and MDA-MB-231 cells (IC_50_ values of 3.54 μM, 8.2 μM, 8.8 μM, and 11.0 μM), respectively, proving its high safety profile. On the other hand, the standard (**VX-680**) produced 9.2 μM IC_50_ value on Fibroblasts, and thus considered safe for the use against PANC1 and MDA-MB-231 cells (i.e., IC_50_ ≈ 7.6 μM on both types). However, (**VX-680**) has demonstrated unfavorable or low safety profile upon the treatment of T-47D and PC-3 cancer as it produced IC_50_ values (i.e., 17.9 μM and 21.4 μM, respectively) on both cell-lines higher than what it produced against fibroblasts (IC_50_ = 9.2 μM), employing that (**VX-680**) concentration required to defeat cancerous causes the death of normal cells.

At last, hit **112 (NCI 12415)** produced 17.9 μM IC_50_ against MDA-MB-231, and scored 15.1 μM IC_50_ against fibroblasts, indicating toxicity and low safety.

## 3. Discussion and Conclusions

Aurora-A kinase inhibitors have shown promising results in suppressing many types of human cancers, because it is involved in regulating multiple molecular targets and signaling pathways, specifically in mitosis. Therefore, Aurora-A is considered a promising kinase to be targeted via designing new inhibitors against it. In this research, a structure-based computational workflow was implemented to explore the structural features required to design new anti-Aurora-A kinase molecules by employing seventy-nine Aurora-A kinase known inhibitors and utilizing an innovative structure-based methodology, i.e., dbCICA, to identify the best possible docking settings required to fit the seventy-nine inhibitors into the active binding site pocket of Aurora-A kinase protein structure. Optimal dbCICA models were converted into corresponding pharmacophore models. The model that had the optimal quality of capturing the active hits and discarding the inactive molecules (i.e., SB-1) was used after refinement as an in-silico search query to screen the National Cancer Institute chemical structural database. The (SB-2) dbCICA model was used for purposes of re-evaluating the activity predicted by (SB-1) or to assess the similarity extent in “predicted activity” between the two models.

Previous studies have shown that there are three active site residues of Aurora-A including (Leu215, Thr217, and Arg220) that lead to selective Aurora-A inhibition upon targeting these residues in the active site [39,92]. It is noteworthy to emphasize that Arg220 and Leu215 are two critical binding site contacts determined by the (SB-2) dbCICA model (Table 2). The Refined-Hypo (SB-1) was adopted in this research because it demonstrated better ROC analysis than (SB-2) model, additionally, the predicted activity by (SB-2) was taken into consideration upon requesting the hits from NCI (Table S3 in Supplementary Material).

Looking into the FRET-based kinase inhibition assay results (Table 5) the IC_50_ values for the Hits **88 (NCI 1576)**, **85 (NCI 14040) and 130 (NCI 12849)** were equal to 2.6 μM, 4.3 μM, and 5.9 μM, respectively. Although there is a minimal mismatch in the hierarchy between the predicted activities employed by (SB-1) model and the activities obtained by FRET-based kinase assay, however, it is a considered a minimal difference in hierarchy because their IC_50_ values are relatively similar. It must be noted that predicted bioactivity obtained by dbCICA models were utilized to prioritize the captured hits for subsequent in vitro testing, bearing in mind that dbCICA approach succeeded in extracting 75 suggested molecules out of an enormous number of compounds in the NCI database, from which 5 molecules are active anti-Aurora-A inhibitors with a top-ranking potent lead inhibitor against four cancer types with a favorable safety profile.

Interestingly, the predicted activity by (SB-1) model, whose refined pharmacophore was used as 3D search query, has revealed that hit **85 (NCI 14040)** is the most effective Anti-Aurora-A among the five active hits (i.e., predicted Ki = 1.2 nM). This result is consistent with the discovery of the effect that hit **85 (NCI 14040)** produce as the most potent inhibitor against PANC1, PC-3, T-47D, and MDA-MB-231 cells (IC_50_ values of 3.54 μM, 8.2 μM, 8.8 μM, and 11.0 μM), respectively, demonstrating consistency between cell-based assay and dbCICA prediction in this case.

The increasing availability of crystal structures of Aurora-A Kinase with different inhibitors will allow further optimization of existing leads as well as the discovery of new ones. Moreover, the newly identified compounds belong to distinct chemical classes than the existing inhibitors, shown in Figure 1, in which Aurora-A kinases comprise successive donor-acceptor functional groups, accordingly, this research enlarges the arsenal of chemical scaffolds for additional rational drug design. The subsequent future lead optimization research will be further assisted by modifying functional groups to approach more selective, more potent, and less toxic compounds.

Although this research study delivers new inhibitors against Aurora-A kinase with new chemical structural backbone compared to the previously published Aurora-A inhibitors employed in clinical trials, however, some limitations need to be addressed here and examined in future work. Bioactivity evaluation should be estimated from three independent experiments in duplicates or triplicates at each concentration point. Permeability and selectivity of these inhibitors should be investigated. As a beneficial improvement; the Hypo(SB-2) pharmacophore could be utilized for in silico 3D search query to explore the NCI library for additional possible Aurora-A kinase inhibitors rather than using it for purposes of validating the activity predicted by Hypo(SB-1), as previous studies have shown that there are three active site residues for Aurora-A (i.e., Leu215, Arg220 and Thr217) that lead to selective Aurora-A kinase inhibition upon targeting these residues [39,92] and it is notable that Leu215 and Arg220 are two critical binding site contacts defined by the (SB-2) dbCICA model (Table 2) which implies that (SB-2) might deliver promising selective anti-Aurora-A kinase molecules.

## 4. Materials and Methods

### 4.1. Materials 

#### 4.1.1. Software and Hardware

The following software packages were utilized in the present research:

- ChemDraw Ultra, Cambridge Soft Corp., Cambridge, MA, USA (Version 7.0.3)
- Marvin View, ChemAxon Ltd., San Diego, CA, USA (Version 5.1.4)
- DiscoveryStudio, Biovea Inc., San Diego, CA, USA (Version 2.5.5)
- MATLAB, MathWorks Inc. Dublin, Ireland (Version 7.4.0.287)

#### 4.1.2. Biological Assay Materials

Dulbecco’s Modified Eagle Medium (DMEM) high glucose (Caisson Labs, UT, USA), RPMI-1640 medium (Caisson Labs, UT, USA), Dulbecco’s Modified Eagle Medium without phenol red (Caisson Labs, UT, USA), Fetal Bovine Serum Heat Inactivated (Capricorn, Ebsdorfergrund, Germany), Phosphate Buffer Saline (Caisson Labs, UT, USA), Trypsin-EDTA 0.25% (Capricorn, Ebsdorfergrund, Germany), Trypan blue dye (Sigma-Aldrich, USA), Tetrazolium dye 3-(4,5-dimethylthiazol-2-yl)-2,5-diphenyltetrazolium bromide (MTT) powder kit (Sigma-Aldrich, St. Louis, MO, USA), Dimethyl sulfoxide DMSO cell culture grade (Sigma-Aldrich, St. Louis, MO, USA), T-75 Treated Flasks (Corning, One Riverfront Plaza, NY, USA), 96-Well Tissue Culture Flat Bottom Treated Microplates (Corning, One Riverfront Plaza, NY, USA), Aurora-A Kinase Standard Inhibitor (VX-680) cat. no. 5907 (Tocris Bioscience, Minneapolis, MN, USA. Cell were originally purchased from the American Type Culture Collection (ATCC, Manassas, VA, USA). The tested cell lines ATCC cat. numbers: triple negative breast adenocarcinoma MDA-MB-231 (HTB-26), pancreas adenocarcinoma PANC1 (CRL-1469), ductal Breast carcinoma T-47D (HTB-133), and prostate adenocarcinoma PC-3 (CRL-1435).

### 4.2. Method

**Figure 7:**
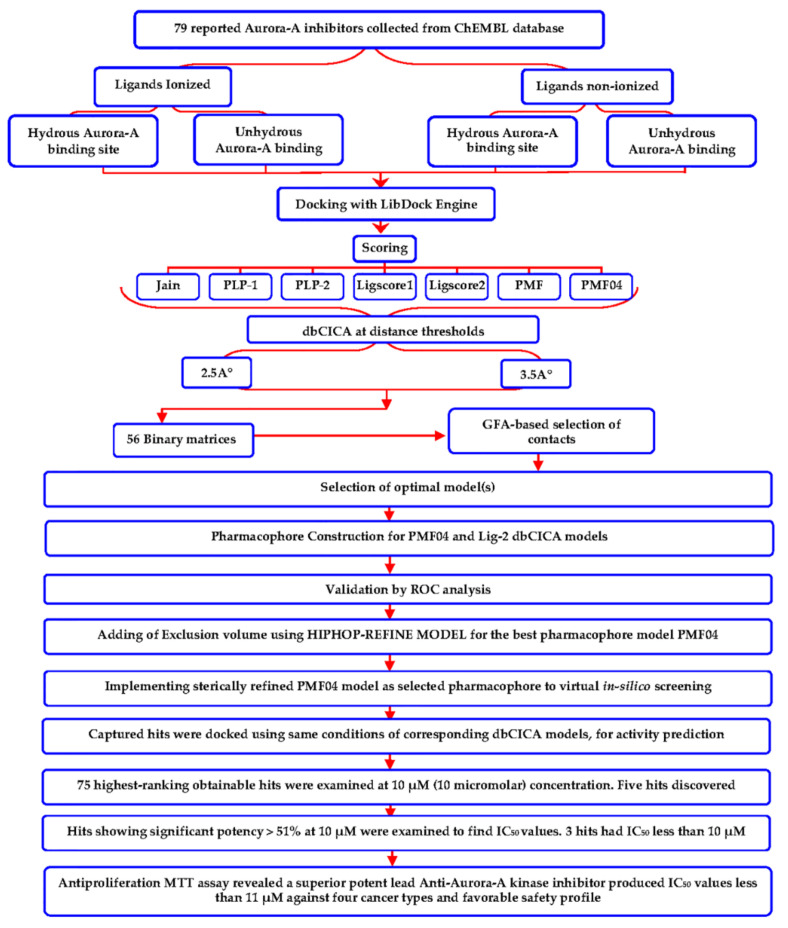
Summary for adopted structure-based modeling and biological evaluation workflow.

The workflow adopted in this research project is summarized in Figure 7.

#### 4.2.1. Molecular Modeling

##### Data Set for Aurora-A Kinase inhibitors and Ligands Preparation

(ChEMBL) database (https://www.ebi.ac.uk/chembl/) was searched for Aurora-A Kinase inhibitors. The search identified 1168 available Aurora-A Kinase inhibitors, they were downloaded and carefully inspected to confirm the consistency of their bioassay procedure standardized with a well-known Aurora-A kinase standard inhibitor. The consistent bioassay determining their bioactivity (Ki value) is affinity or binding kinase assay with minor differences in the procedure, any molecule assayed functionally was not utilized. List of seventy-nine Aurora-A Kinase inhibitors were collected for modeling from six literature articles [42,43,44,70,71,72]. The consistency of the bioassay data is critical for quantitative structure–activity relationship (QSAR) modeling including dbCICA [56,57,61]. The collected list of compounds and their reported Ki values (in (nM)) are demonstrated in Table S1 under Supplementary Material. The logarithmic transformations of 1/Ki were used in structure–activity correlation in order to correlate free energy change to the bioactivity linearly. 

The chemical structures of the collected compounds were exported from ChEMBL in simplified molecular-input line-entry system (SMILES) format. Subsequently, they were imported into DiscoveryStudio (version 2.5.5) [81], and transformed automatically into a reasonable single conformer 3D structure using rule-based methods implemented in DiscoveryStudio 2.5.5 [81], and saved in structure database format for subsequent docking experiments. For each inhibitor, two protonation states were considered: un-ionized and ionized as guided by MarvinView (Version 5.1.4) at physiological pH (7.4). In the ionized structures, amino substituent (pKa ≈ 9.0–9.5) were protonated and granted positive charges while the carboxylic acids (pKa ≈ 4.5) were deprotonated and granted formal negative charges. The unionized and ionized lists were utilized in further docking experiments. 

##### Preparation of Aurora-A Kinase Crystal Structure

The crystallographic 3D coordinates of Aurora-A kinase protein were retrieved from the Protein Data Bank (PDB code: 3w2c, resolution = 2.45 Å). Hydrogen atoms were added to the protein utilizing DiscoveryStudio 2.5.5 templates for protein residues. Energy minimization was not performed upon using the protein structure for docking. Explicit water molecules were either removed or kept according to the desired docking conditions, i.e., docking in hydrous or anhydrous binding pocket. The binding pocket was defined as the cavity volume occupied by co-crystallized ligand within the PDB protein (3w2c).

##### Docking and Scoring

Docking experiments were performed using LibDock docking engine, which consider the flexibility of the ligand while treat the receptor as rigid [59,73]. Docking was performed by using the seventy-nine Aurora-A Kinase inhibitors with known (absolute) stereochemistries as ligands (1–79, Table S1 in Supplementary Material) after removing hydrogen atoms into the (3w2c) protein structure putative active site/binding pocket guided by binding hotspots. Polar and apolar receptor interactions hotspots align the ligand conformations [59,73]. Details of LibDock docking process and corresponding docking settings are described in Section S1 in the Supplementary Materials. 

The highest ranking docked poses/conformers generated by LibDock were scored using 7 scoring functions: Jain [74], LigScore1, and LigScore2 [75], PLP1, PLP2 [76], PMF and PMF04 [77,78], for details refer to Section S2 in the Supplementary Materials. This step resulted in seven groups of docking/scoring combinations for each of the seventy-nine compounds. The docking/scoring cycles were repeated 4 times (2 × 2) to cover the different combinations of docking conditions, i.e., ionized ligand and unionized ligand, and hydrous binding site and anhydrous binding site. The docking experiment yielded 28 sets of docked poses (in SD format) that represent the combination of seven scoring functions, unionized vs. ionized ligands, un-hydrous vs. hydrous binding sites. The highest scoring docked pose for each inhibitor was chosen to be used in subsequent (dbCICA) modeling [56,57,60,61,62,63,64,65,66,67].

##### Docking-Based Comparative Molecular Contacts Analysis (dbCICA)

This methodology was performed through the following steps:

1. Allocating binary code based on neighboring contacts: the closest binding site atoms for each docked conformer of each ligand was identified. The neighboring atoms lying exactly at or closer than a predefined distance-threshold of 3.5 Å or 2.5 Å are allocated an intermolecular contact value of “one”, otherwise they are given a contact value of “zero”. The distance evaluation was automatically performed via employing the Intermolecular Monitor of DS 2.5.5.
2. A 2D matrix is engendered for every docking-scoring configuration, such that each matrix is composed of column labels correlated to different binding site atoms and row labels correlated to docked ligands. The binary code is filled into the matrix, by which “zeros” correspond to inter-atomic distances that exceed the predetermined threshold and “ones” for distances equal or below the predefined threshold cutoffs.
3. Two binary matrices (each one relevant to distance threshold: 2.5 Å or distance threshold: 3.5 Å) were constructed for each docking configuration (i.e., **2** protein hydration states × **2** ligand ionization states × **2** distance thresholds × **7** scoring functions). Accordingly, 56 binary files were generated in the study.
4. Each individual column in the matrix was regressed against respective molecular bioactivities (i.e., −log (Ki)). If the columns showed negative correlation with bioactivity, thus, they are inverted, i.e., zeros are converted to ones and vice versa, and excluded from the subsequent step.
5. The remaining binary matrix composed of bioactivity correlated with positive contact columns, is then subjected to genetic algorithm (GA)-based search (within MATLAB) for optimal sum of contacts columns able to explain variations in bioactivity (detailed information on how GA is implemented in dbCICA modeling, is in Section S3 under Supplementary Materials). The dbCICA model is built or represented based on selecting the best column-summation model.
6. GA was directed to deliver best dbCICA models resulting from sets of 4, 5, 6, 7, 8, 9, and 10 concurrent contacts with the best behavior.
7. dbCICA algorithm permits varying contacts-weights to detect intermolecular contacts comprising higher weights contributions in optimal dbCICA models: variable contacts weights emerged by positive contacts column could be up to three times in the summation model. To perform this, dual valued genes are implemented in the GA, whereby every gene encodes not only the respective contacts column number but also its weight.
8. After using positive contacts to identify the optimal summation model(s), subsequently, GA is used to seek the best summation generated by linking inverted columns (exclusion columns, detailed in steps 4 and 5 at Section 4 Docking-Based Comparative Molecular Contacts Analysis (dbCICA)) with the best positive summation model(s). In this project, we executed two exclusion settings; the allowed negative contacts were either five or ten.

##### Manual Generation of Pharmacophores Corresponding to Successful dbCICA Models

Building pharmacophores was guided by using the best dbCICA models. Subsequently, pharmacophores were used as 3D-search queries for the discovery of new Aurora-A kinase inhibitors. The pharmacophores were created via the following steps:

1. The docking setting that comprised the best dbCICA models were nominated. In this project two conditions produced the best models: (1) docking the ionized ligands into hydrous binding site using PMF04 scoring function with five features and ten exclusion volumes, named (SB-1) (2) docking ionized ligands into unhydrous binding site using Ligscore2 scoring function with ten features and ten exclusion volumes, named (SB-2) (Table 1 and Table 2). The docked poses, corresponding to the model of the most potent ligands (Ki ≤ 17 nM), were preserved in the binding pocket while less potent or inactive ligands were discarded.
2. Afterwards, these best dbCICA models are used to predict the bioactivities of the potent Aurora kinase A inhibitors in the binding pocket i.e., by substituting the sum of contacts for every docked molecule in the relevant regression equation associated to the dbCICA model. The well-behaved and potent compounds (i.e., showing minimal difference between real and fitted bioactivities) were maintained in the binding pocket for subsequent steps.
3. Those compounds, having weights of 2 or 3 or major positive molecular contacts in the binding site, were carefully evaluated and annotated to discover the ligands closest moieties. Consensus among potent training molecules to locate moieties of common physicochemical properties next to significant contact atom permit placing a representative pharmacophoric feature onto that region. For example, if potent docked molecules agreed on the presence of aromatic rings next to a certain dbCICA significant contact atom then a hydrophobic aromatic feature is positioned on top of the aromatic rings within the predefined threshold of distance. Manual addition of pharmacophoric features was employed using DiscoveryStudio 2.5.5 feature library and default feature radii (1.6 Å or 2.2 Å).
4. Finally, the steric constraints of the binding pocket were accounted for based on binding-site contact atoms of negative correlations with bioactivity. Negative contacts specify the spaces where the conformers of inactive or minimal active compounds dominate and surely, active ones are absent. Therefore, these exclusion volumes were filled with exclusion spheres manually from DiscoveryStudio 2.5.5 feature library and default feature radius (1.2 Å) was employed.
5. The overall manual pharmacophores modeling procedure generated two pharmacophore models from dbCICA based on: first, ionized ligands into hydrous binding site using PMF04 scoring function with five features and 10 exclusion volumes i.e., Hypo(SB-1). Second, based on ionized ligands into unhydrous binding site using Ligscore2 scoring function with ten features and ten exclusion volumes Hypo(SB-2) (see Table 1 and Table 2, Figure 2 and Figure 3A–T, for model’s summary).

##### Validation of Generated Pharmacophore Models Using Receiver Operating Characteristic (ROC) Curve Analysis 

The classification power of dbCICA derived pharmacophores; Hypo(SB-1) and Hypo(SB-2) was validated using receiver operating characteristic (ROC) curve analysis.

The ROC analysis assesses the ability of the pharmacophore to distinguish between Aurora-A kinase active or inactive group of molecules within a designated large “testing list” and to selectively capture the ones with better activity. This testing list was entirely composed of experimentally validated compounds extracted from the European Bioinformatics Institute database (ChEMBL, https://www.ebi.ac.uk/chembl) and included 86 active compounds (anti-Aurora-A Kinase with Ki values ≤10 nM) and 248 less-active compounds (anti-Aurora-A Kinase with Ki values > 500 nM considered as decoy list).

Selection process of the testing set compounds is detailed in Section S5 under Supplementary Materials. Conformational ensembles were generated for the testing set using “CESEAR” conformation generation option implemented in DiscoveryStudio 2.5.5.

The results were presented in the form of ROC curves (Table 4). ROC analysis provides several success criteria for evaluation: (1) area under the curve (AUC) of the corresponding ROC curve, (2) overall accuracy (ACC), (3) overall specificity (SP), or true negative (TNR), (4) overall sensitivity (SE), or true positive rate (TPR) and (5) overall false positives (FP). ROC curves were plotted by considering the highest score (fit value against the tested pharmacophore) of an active molecule as the first threshold then counting the number of decoy compounds within this cut-off value, and the corresponding sensitivity and specificity pair is calculated. This process is repeated using the active molecule possessing the second highest score and so forth, until the scores of all active compounds are considered as selection cut-off values rate [79,80,93,94]. Details of ROC analysis are shown in Section S4 under Supplementary Materials. dbCICA models ROC analysis curves and results are illustrated in Table 4.

##### Addition of Exclusion Volumes

Based on ROC results and in order to improve the classification properties of Hypo(SB-1) model, which had better behavior over Hypo(SB-2), as detailed in results Section 2.3, and in Table 4, it was decided to complement Hypo(SB-1) with exclusion spheres by employing HIPHOP REFINE module of DiscoveryStudio 2.5.5 [81,82]. HIPHOP REFINE identifies spaces occupied by the conformations of inactive compounds and free from conformations of active ones. These areas are filled with exclusion volumes to represent the steric constrains of the binding pocket [56,82,95]. See Section S5 under Supplementary Materials for more information on HIPHOP REFINE and how it adds exclusion spheres to pharmacophore model Hypo(SB-1)**.** A subset of training compounds was carefully selected for HIPHOP REFINE modeling of the Hypo(SB-1) as detailed in Table S2. This list includes active, moderately active, and inactive compounds. Based on their activity, exclusion spheres occupy the space conformations of inactive compounds and avoid space conformations of active compounds. The HIPHOP REFINE process resulted in adding 93 exclusion volumes to Hypo(SB-1), and the sterically refined pharmacophore was named Refined-Hypo(SB-1). 

The performance of Refined-Hypo(SB-1) was evaluated again by ROC analysis, as illustrated in (ROC) curves in Table 4, and accordingly, Refined-Hypo(SB-1) model was adopted for in-silico search screening, whereas Hypo(SB-2) was used as a confirmatory model for the predicted activity.

##### In-Silico Screening of NCI for new Aurora-A Kinase Inhibitors

The sterically refined dbCICA-derived pharmacophore Refined-Hypo(SB-1) in Figure 2 was used as 3D query to search and screen the National Cancer Institute compounds database (253,368 compounds). Screening was performed by employing the “Fast Flexible Database” search option implemented within DiscoveryStudio 2.5.5, and captured 293 hits that were subsequently filtered based on SMARTS filtration [81] and based on Lipinski rule [86], which yielded 262 hits. Subsequently, conformational ensembles were generated for surviving hits using “CEASAR” conformation generation option within DiscoveryStudio2.5.5 to allow fitting hits against the capturing pharmacophore. The lowest fitting 145 hits (ca. the least 55% against Hypo(SB-1) pharmacophore) were discarded leaving 117 hits for subsequent docking into Aurora-A kinase. 

The remaining 117 hits were docked into Aurora-A kinase protein binding pocket (PDB code: 3w2c) employing the same docking/scoring settings of a corresponding dbCICA model (SB-1) (Table 1 and Table 2, pharmacophores in Figure 2) (i.e., docking the ionized ligands into hydrous binding site using PMF04 scoring function with five features and ten exclusion volumes). Steps of activity prediction were also employed using docking/scoring settings of (SB-2) dbCICA model to assess the similarity extent in “predicted activity” of (SB-2) model and the better performing (SB-1) model. Therefore, the remaining 117 hits were also docked into Aurora-A Kinase binding pocket (3w2c) using the docking-scoring settings of (SB-2) dbCICA model i.e., docking ionized ligands into unhydrous binding site using Ligscore2 scoring function with ten features and ten exclusion volumes.

The resulting docked poses (i.e., of hits) were analyzed to identify their binding critical contacts. The sums of critical contacts (as identified by corresponding dbCICA model, Table 1 and Table 2) for each compound were used to predict their corresponding Ki values or bioactivities by substituting their sum of contacts with binding site atoms in the respective dbCICA-regression equations (Table 1), tying the contacts sums with bioactivities. 

The process of hits request was done in accordance with the predicted activity of (SB-1) model (i.e., whose settings were used as the 3D search query derived pharmacophore Refined-Hypo(SB-1), and after a voting step to prioritize the hits that scored high predicted activity via (SB-2) dbCICA model, and lastly, based on the compounds’ availability at NCI. The highest-ranking 75 obtainable hits were requested from the NCI for subsequent enzyme inhibition assay and in-vitro cell-based biological evaluation. Table S3 and Figure S2 in Supplementary Material summarizes the structures of all the 75 captured hits (numbered 80–154) obtained by in-silico screening, their contact atoms summation, and their predicted bioactivities. 

#### 4.2.2. Biological Evaluation of Captured Hits

##### FRET-Based Enzyme Inhibition Assay

The activity, the 75 captured hits, was evaluated using enzyme inhibition assay. This bioassay was conducted using Z′-LYTE^®^ kinase assay (Invitrogen, Carlsbad, CA, USA) [68,69]. This biochemical assay depends on fluorescence emission as detection method utilizing a synthetic fluorescence resonance energy transfer (FRET) peptide with two fluorophores: coumarin that work as donor and fluorescein as acceptor [69]. Each hit or compound was dissolved in DMSO to form 10 mM stock solution. Hits were screened at 10 μM concentration. The bioassay was performed in black 384-well plate. For each assay, 100 nL of tested compound stock solution (10 mM) was mixed with 2.4 μL kinase buffer, 5 μL (peptide substrate/Aurora-A Kinase) mixture, and 2.5 μL ATP solution. The final 10 μL kinase reaction consisted of (0.91–8.56) ng Aurora-A Kinase (in DMSO < 1%), and 2 μM Ser/Thr 01 (kinase peptide substrate from Z′-LYTE^®^ (Invitrogen, Carlsbad, CA, USA) and 10 μM ATP in 50 mM HEPES pH 7.5, 0.01% BRIJ-35, 10 mM MgCl_2_, and 1 mM EGTA. The mixture was incubated at room temperature for 60 min to allow the kinase reaction to complete. Subsequently, 5-μL development reagent A (from Z′-LYTE^®^, 1:4096 dilution) were added to the reaction mixture and shaken over 30 s. The mixture was incubated for another 60 min at room temperature before the fluorescence is measured at λEx of 445 and λEm of 520 nm (λEx: excitation wavelengths, λEm: emission wavelengths).

For initial screening, the 75 hits were examined at 10 μM. Five compounds have shown anti-Aurora-A Kinase inhibition percentages > 51% at 10 μM (Table 5), which were further tested at several concentrations range (0.1–100 μM) to determine their anti-Aurora-A Kinase IC_50_ values. The tests were conducted in duplicates. Staurosporine was used as control (standard inhibitor with IC_50_ of 3.72 nM) [62]. Nonlinear regression of log (concentration) vs. inhibition percentages values using GraphPad Prism 7.03 software was adopted to plot the dose–response curves (Figure 5) and calculate IC_50_ values (Table 5). 

The five bioactive hits were further examined using Cell Culture Based Cytotoxicity Assay.

##### Cell Culture Based Antiproliferation Assay

The human cell-lines triple negative breast adenocarcinoma MDA-MB-231, Pancreas adenocarcinoma PANC1, ductal breast carcinoma T-47D and fibroblasts were preserved and expanded in DMEM medium, while the prostate adenocarcinoma PC-3 cells were cultured in RPMI-1640 growth medium. All growth media were supplemented with 10% heat-inactivated fetal bovine serum (FBS). Cells were maintained under standard culture conditions at 37 °C, 5% CO_2_ in a humidified incubator. At 80% confluency, cells were washed with 5 mL phosphate buffer saline (1X PBS), then incubated for 4 min with 2–3 mL Trypsin-EDTA 0.25% in order to obtain suspended cells and collect them in a known volume of fresh media for cell count. Cells were counted using conventional hemocytometer and trypan blue dye method.

The five potent hits that have shown anti-Aurora-A Kinase inhibition percentages above 51% at 10 μM upon conducting the kinase enzyme Inhibition assay (Table 5) were further validated using MTT cytotoxicity Assay. This assay includes 3-(4,5-Dimethylthiazol-2-yl)-2,5-diphenyl tetrazolium bromide (MTT) dye, which is a yellowish tetrazole reduced by viable cells into formazan purple color, which detects the normal mitochondrial role in viable cells. In 96-well coated flat bottom microplates, 100 μL cell suspension of PANC1 or T-47D cells were seeded at a density of 7000 cell/well, and 6000 cell/well for MDA-MB-231, PC-3, or fibroblasts, and incubated under the same conditions at 37 °C. After 24 h, each well was treated with the intended drug concentration diluted in 200 μL fresh media after removal of the old 100-μL media. After 72 h incubation, drug-containing media was aspirated and on each well 100 μL phenol-red free media and 15-μL MTT solution (5 mg/mL (wt/vol) PBS). After 3 h of dark incubation at 37 °C, MTT and media were carefully withdrawn from each well, and 200 μL/well DMSO were added. Colorimetric absorbance (OD) was measured at 570 nm wavelength using 96-well plate reader (Tecan-Sunrise, Männedorf, Switzerland).

The five compounds were prepared in a stock concentrate of 10 mM using cell culture grade DMSO to be utilized in MTT assay. The cytotoxicity of each compound was initially screened on cancer cell-lines at two concentrations: 50 μM and 25 μM in duplicates. Wells treated with 200 μL media plus DMSO only (0.5% *v*/*v*) represented negative control and the standard inhibitor Tozasertib/(VX-680) was used as positive control. The outgrowth inhibition rate or (cell death) was calculated using this formula: outgrowth inhibition % = (1 − (mean (ab treated)/mean (ab control))) × 100%. Subsequently, compounds that produced cell death or inhibition rate above 50% at 50 μM were further tested to determine their half-maximal inhibitory concentration (IC_50_) values. Cells were treated with multiple decreasing concentrations of the tested compound starting at 50 μM in triplicates for IC_50_ determination. The safety index for the compounds with specified IC_50_ values was evaluated via determining their IC_50_ values against normal Fibroblast cells. It is noteworthy to mention that DMSO concentration was constant on all the wells (i.e., (0.5% *v*/*v* or 1 μL/200 μL) per well), for the treated and the control ones. IC_50_ values were calculated using nonlinear regression of the dose–response curves using GraphPad Prism software 7.03.

## Figures and Tables

**Table 1:** Highest ranking docking-based Comparative Intermolecular Contacts Analysis (dbCICA) models, their corresponding parameters, and statistical criteria.

db-CICA Model	Docking Engine	Scoring	Ligands Ionization State	Explicit Water ^a^	Contacts Distance Threshold ^b^	Number of Positive Contacts ^c^	Number of Negative Contacts ^d^	r^2^ ^e^	r^2^_LOO_^f^	r^2^_5-fold_^g^	Regression Equation ^h^	F statis-tic ^i^
SB-1	Lib-Dock	PMF04	Ionized	Present	3.5	5	10	0.61	0.60	0.59	Y = 0.318X + (−4.218)	124.28
SB-2		Ligscore2		Absent	3.5	10	10	0.61	0.59	0.60	Y = 0.21486X + (−3.850)	121.84

^a^ Crystallographically explicit water of hydration. ^b^ Distance thresholds used to define ligand-binding site contacts. ^c^ Optimal number of combined (i.e., summed) bioactivity-enhancing ligand/binding site contacts. ^d^ Optimal number of combined bioactivity-disfavoring ligand/binding site contacts. ^e^ Non-cross-validated correlation coefficient for training compounds. ^f^ Cross-validation correlation coefficients determined by the leave-one-out technique. ^g^ Cross-validation correlation coefficients determined by the leave-20%-out technique repeated 5 times. ^h^ X and Y represent contact summation and predicted bioactivities. ^i^ F-statistic, which was calculated from the correlation connecting (−log(Ki)) and contacts sums of the corresponding dbCICA models.

**Table 2:** Critical Binding Site Contact Atoms Proposed by Optimal dbCICA Models.

dbCICA Model	Favored Contact Atoms (Positive Contacts) ^a,b^		Disfavored Contact Atoms (Negative Contacts) ^e^
	Amino Acids & Corresponding Atom Identities ^c^	Weights ^d^	
SB-1	ARG137:NH2	1	ALA160:CB, ALA213:O, ALA273:CB, ASN146:HD22, LEU194:CG, LEU210:HD21, LYS143:CB, LYS162:CD, PHE144:CE1, PHE144:HN
	LEU210:CG	3	
	VAL147:HG13	2	
	VAL182:N	3	
	TYR212:CZ	3	
SB-2	ARG137:HD2	3	ASP274:C, ASP274:HA, ASP274:OD2, GLN185:OE1, GLU181:OE2, LEU208:HD11, LYS143:C, LYS258:HD2, LYS258:HZ2, PHE144:HB1
	ARG137:NE	1	
	ARG220:CA	2	
	ARG220:HB1	1	
	ASN261:HA	3	
	GLY216:HA1	3	
	ILE184:HG12	2	
	LEU208:HD13	3	
	LEU215:C	3	
	LYS224:HZ3	3	

^a^ As in Table 1. ^b^ Bioactivity-proportional ligand/binding site contacts. ^c^ Binding site amino acids and their significant atomic contacts. Atom codes are as provided by the protein data bank file format ^d^ Degree of significance (weight) of corresponding contact atom, which points to times it emerges in the final dbCICA model (see point 7 in Section 4 Manual Generation of Pharmacophores Corresponding to Successful dbCICA Models). ^e^ Bioactivity disfavoring ligand/binding site contacts.

**Table 3:** The three-dimensional (3D) coordinates and feature tolerances of dbCICA-derived pharmacophore models.

Pharmacophoric Model ^a^	Definition	Chemical Features							
Hypo (SB-1) **^a^**			**HBA**		**RingArom**		**Hbic**	**RingArom**	
	Tolerances Coordinates		1.6	2.2	1.60	1.60	1.60	1.60	1.60
		X	0.324	0.163	6.568	3.769	8.939	3.417	1.745
		Y	13.041	16.035	6.83	6.541	5.327	11.244	11.825
		Z	3.095	3.202	10.643	11.683	12.459	3.855	6.277
Hypo (SB-2) **^b^**			**HBA**		**RingArom**		**Hbic**	**Hbic**	
	Tolerances Coordinates		1.6	2.2	1.60	1.60	1.60	1.6	
		X	−2.411	−1.979	1.744	−0.433	−1.303	−4.344	
		Y	12.334	14.938	10.567	12.045	9.181	14.013	
		Z	3.715	2.289	3.554	4.994	1.792	6.188	

**^a^** Hypo(SB-1): corresponds to the pharmacophore model generated by SB-1. The pharmacophore model includes 10 exclusion volumes of 1.2 Å tolerance spheres and at the following (X,Y,Z) coordinates: (7.878, 13.543, 9.28), (6.424, 12.095, 0.897), (8.194, 3.839, 7.994), (2.467, 7.261, 17.321), (11.495, 7.827, 6.688), (11.288, 8.067, 12.452), (−1.989, 3.309, 13.359), (6.186, 8.159, 15.172), (3.669, 4.225, 10.264), and (−1.091, 5.891, 10.773). **^b^** Hypo(SB-2): corresponds to the pharmacophore model generated by SB-2 The pharmacophore model includes 10 exclusion volumes of 1.2 Å tolerance spheres and at the following (X,Y,Z) coordinates: (10.063,−0.259,11.156), (8.978,1.223,12.225), (5.209,0.525,11.361), (13.663,3.897,12.785), (15.219,0.329,23.239), (9.255, 7.726, 18.79), (−0.454, 5.232, 12.634), (2.016, −4.85, 7.403), (1.366, −3.582, 9.269), and (0.397,7.202,9.312).

**Table 4:** ROC performances of dbCICA pharmacophores and the refined Hypo(SB-1) pharmacophore model using Testing Set lists.

List Used	Testing Set				ROC ^a^ Curve
Pharmacophore	ROC ^a^- AUC ^b^	ACC ^c^	TPR ^d^ (SE)	TNR ^e^ (SP)	
Hypo(SB-1)	0.83	0.64	0.72	0.61	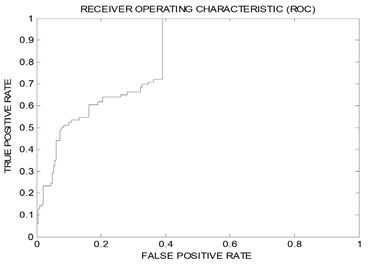
Hypo(SB-2)	0.80	0.56	0.93	0.44	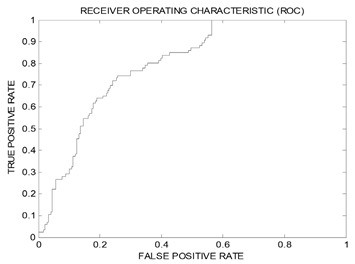
Refined-Hypo(SB-1)	0.91	0.73	0.24	0.90	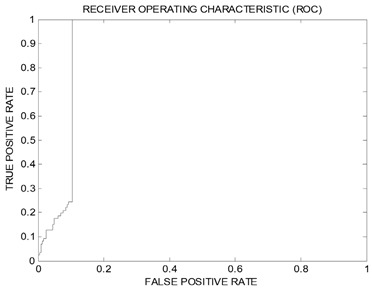

^a^ ROC: receiver operating characteristic. ^b^ AUC: area under the curve. ^c^ ACC: overall accuracy. ^d^ TPR: overall true positive rate, also called selectivity (SE). ^e^ TNR: overall true negative rate, also called specificity SP.

**Table 5:** The IC_50_ values of the five active hits that produced anti-Aurora-A kinase inhibition above 50% at 10 µM using Z′-LYTE^®^ kinase assay (Invitrogen, Carlsbad, CA, USA), sum of their critical contacts, and their predicted activity. These high-ranking hits captured by Refined-Hypo(SB-1) derived from Hypo(SB-1) pharmacophore were docked into Aurora-A Kinase binding pocket (3w2c) using the docking-scoring settings of (SB-1). Their docked poses were analyzed to identify their critical binding contacts (marked by dbCICA model Table 2) and were used to predict their Ki values or activities by substituting the sum of binding contacts in the respective dbCICA regression equations (Table 1). Steps of activity prediction were also employed using docking/scoring settings of (SB-2) dbCICA model to assess the similarity extent in “predicted activity” of both (SB-2) model and the better performing (SB-1) model.

Hit (NCI Code Number) ^a^		SB-1		SB-2		% Inhibition at 10 µM ^d^ (±SEM)	IC_50_ (µM) ^e^	Hill Slope ^e^
		Sum of Contact Atoms ^b^	Predicted Ki Values ^c^ (nM)	Sum of Contact Atoms ^b^	Predicted Ki Values ^c^ (nM)			
**85**	**(NCI 14040)**	13	1.20	13	11.41	56 ± (1.7)	4.3	1.70
**88**	**(NCI 1576)**	12	2.51	14	6.96	75 ± (0.2)	2.61	1.77
**112**	**(NCI 12415)**	10	10.87	14	6.96	55 ± (5.5)	ND ^f^	-
**130**	**(NCI 12849)**	9	22.62	13	11.41	86 ± (0.2)	5.9	7.6
**141**	**(NCI 12492)**	8	47.07	11	30.71	51 ± (0.5)	ND ^f^	-

**^a^** Group of the highest-ranking hits obtainable from NCI (numbered 80–154) summarized in Table S3 and Figure S2 under Supplementary Material for structures, sum of contacts, predicted activity and % Inhibition at 10 µM. **^b^** Contacts summations according to corresponding dbCICA model (Table 1 and Table 2). **^c^** Predicted Ki by substituting the sum of contacts of each docked hit in the regression equation of corresponding dbCICA model. **^d^** Percentage inhibition determined for duplicate measurements at 10 µM, standard error was calculated for duplicates at this concentration using functions of Microsoft excel software. **^e^** IC_50_ and Hill slope values analyzed using GraphPad Prism software (version 7.03). Multiple concentrations range (0.1–100 μM) used to determine IC_50_ values each were conducted in duplicates, the standard error for each concentration used in IC_50_ measurement are summarized in Supplementary Material Table S3. IC_50_ was calculated using nonlinear regression of log (concentration) vs. inhibition % values plotted in Figure 5. **^f^** Not Detected.

**Table 6:** The concentrations of the five active hits that prevented cell growth by 50% or (IC50) values and their anti-Aurora-A kinase inhibition % at 50 µM and 25 µM after 72 h against different cancer cell lines compared to the Aurora-A kinase standard inhibitor (VX-680).

Drug Number ^a^		141 (NCI 12492)	130 (NCI 12849)	112 (NCI 12415)	88 (NCI 1576)	85 (NCI 14040)	Tozasertib/ (VX-680) ^d^
		**Cell-line: MDA-MB-231**					
% Inhibition ^b^	At 50 µM	11.1 ± 0.05	45.5 ± 0.05	74.7 ± 0.012	31.3 ± 0.06	87.7 ± 0.001	87.0 ± 0.007
	At 25 µM	2.9 ± 0.03	22.9 ± 0.02	63.3 ± 0.021	21.1 ± 0.05	82.3 ± 0.006	81.0 ± 0.008
IC_50_^c^ (µM)		>50	>50	17.9 ± 1.4	>50	11.0 ± 0.43	7.7 ± 0.65
		**Cell-line: Panc1**					
% Inhibition ^b^	At 50 µM	15.7 ± 0.02	34.9 ± 0.017	22.5 ± 0.012	15.1 ± 0.006	94.1 ± 0.001	89.3 ± 0.015
	At 25 µM	11.4 ± 0.003	20.3 ± 0.012	16.0 ± 0.02	11.3 ± 0.01	92.97±0.004	80.9 ± 0.005
IC_50_^c^ (µM)		>50	>50	>50	>50	3.54 ± 0.11	7.6 ± 0.8
		**Cell-line: T-47d**					
% Inhibition ^b^	At 50 µM	25.04 ± 0.02	31.3 ± 0.008	48.11 ± 0.003	34.9 ± 0.013	86.0 ± 0.002	80.9 ± 0.01
	At 25 µM	21.11 ± 0.01	25.5 ± 0.016	34.7 ± 0.012	26.5 ± 0.01	84.6 ± 0.002	53.4 ± 0.02
IC_50_^c^ (µM)		>50	>50	>50	>50	8.8 ± 0.74	17.9 ± 1.64
		**Cell-line: PC-3**					
% Inhibition ^b^	At 50 µM	ND	ND	32.7	ND	87.3 ± 0.003	68.7 ± 0.011
	At 25 µM	ND	ND	19.2	ND	77.0 ± 0.007	49.5 ± 0.006
IC_50_^c^ (µM)		ND	ND	>50	ND	8.2 ± 1.4	21.4 ± 1.60
		**Cell-line: Fibroblasts**					
% Inhibition ^b^	At 50 µM	ND	ND	54.3 ± 0.003	ND	83.4 ± 0.01	89.0 ± 0.005
	At 25 µM	ND	ND	53.9 ± 0.018	ND	45.4 ± 0.05	85.0 ± 0.006
IC_50_^c^ (µM)		ND	ND	15.1 ± 3.0	ND	27.5 ± 1.5	9.2 ± 0.6

ND: Not Detected; limited availability from NCI, MDA-MB-231: triple negative breast adenocarcinoma, PANC1: pancreas adenocarcinoma, T-47D: ductal breast carcinoma, PC-3: prostate adenocarcinoma cells. ^a^ Group of the highest-ranking hits obtainable from NCI (numbered 80–154) summarized in Table S3 and Figure S2 under Supplementary Material, ^b^ % Inhibition was determined using the formula: outgrowth inhibition % = (1 − (mean (Ab treated)/mean (Ab control)) * 100%. Each concentration (in duplicates), ^c^ The (IC_50_) values and their data standard error of the mean (±SEM) were calculated using nonlinear regression of the dose-response curves using GraphPad Prism (version 7.03). Cells treated with increasing serial concentrations of the tested compound in triplicates, ^d^ Tozasertib/(VX-680): Aurora-A kinase standard inhibitor, chemical structure in Figure 1. Standard error for % Inhibition at 50 µM and 25 µM were calculated using Microsoft Excel software functions.

## Supplementary Materials

The following are available online, Table S1: The structures of the seventy-nine Aurora-A Kinase inhibitors collected from the (ChEMBL), utilized in modeling. Table S2: Refinement list for steric refinement of Hypo(SB-1). Table S3: The 75 high-ranking hits captured by Refined-Hypo(SB-1) 3D search query-obtained from NCI and their anti-Aurora-A kinase inhibition % at 10 µM using Z′-LYTE^®^ kinase assay (Invitrogen, Carlsbad, CA, USA), sum of their critical contacts and their predicted activity. Section S1: LibDock Docking Engine and Scoring Settings. Section S2: Scoring of Docked Ligand Poses. Section S3: Genetic Algorithm Implementation in dbCICA Modeling. Section S4: receiver operating characteristic (ROC) curve analysis. Section S5: Steric Refinement of pharmacophores. Figure S1: Three-dimensional plot showing three main principal components calculated for the Testing Set of for HIPHOP REFINE modeling. Figure S2: The chemical structures of the tested highest-ranking hits. Figures S3–S10: “NMR” and Mass Spectrum for Hit 88 (NCI 1576). Figure S11–S18: “NMR” and Mass Spectrum for Hit 130 (NCI 12849). Figures S19–S25: “NMR” and Mass Spectrum for Hit 85 (NCI 14040), Figures S26–S32: “NMR” and Mass Spectrum for Hit 112 (NCI 12415). Figures S33–S38: “NMR” and Mass Spectrum for Hit 141(NCI 12492).
