# Supplementary material for: Structure-Based Discovery and Bioactivity Evaluation of Novel Aurora-A Kinase Inhibitors as Anticancer Agents via Docking-Based Comparative Intermolecular Contacts Analysis (dbCICA)
Source: Molecules. 2020 Dec 18;25(24):6003. doi: 10.3390/molecules25246003 (PMC7766225; doi:10.3390/molecules25246003)
Supplement: Supplementary file 1 [file molecules-25-06003-s001.pdf]

Supplementary Materials

Structure-Based Discovery and Bioactivity Evaluation of Novel Aurora-A Kinase Inhibitors as Anti-Cancer Agents via Docking-based Comparative Intermolecular Contacts Analysis (dbCICA)

Majd S. Hijjawi [1], Reem Fawaz Abutayeh [2] and Mutasem O. Taha [3,*]

[1] Department of Pharmacology, Faculty of Medicine, The University of Jordan, Amman, Jordan; mjd8120578@fgs.ju.edu.jo; (M.H.)
[2] Department of Pharmaceutical Chemistry and Pharmacognosy, Faculty of Pharmacy, Applied Science Private University, Amman, Jordan; r_abutayeh@asu.edu.jo; (R.A.)
[3] Department of Pharmaceutical Sciences, Faculty of Pharmacy, University of Jordan, Amman, Jordan; mutasem@ju.edu.jo; (M.T.)
*Correspondence: mutasem@ju.edu.jo; Tel.: +962-6-5355000

Table of Contents	
Tables	• **Table S 1:** The structures of the (79) seventy-nine Aurora-A Kinase inhibitors collected from the (ChEMBL), utilized in modeling.<br>• **Table S 2**: Refinement list for steric refinement of Hypo(SB-1)<br>• **Table S 3:** The 75 high-ranking hits captured by Refined-Hypo(SB-1) 3D search query-obtained from NCI and their anti-Aurora-A kinase inhibition% at 10 μM using Z'-LYTE kinase assay, sum of their critical contacts and their predicted activity.
Sections	• **Section S 1:** LibDock Docking Engine and Scoring Settings<br>• **Section S 2:** Scoring of Docked Ligand Poses.<br>• **Section S 3:** Genetic Algorithm Implementation in dbCICA Modeling<br>• **Section S 4:** Receiver-operating characteristic (ROC) curve analysis<br>• **Section S 5:** Steric Refinement of pharmacophores
**Figures**	• **Figure S 1:** Three-dimensional plot showing three main principal components calculated for the Testing Set of for HIPHOP-REFINE modelling<br>• **Figure S 2:** The chemical structures of the tested highest-ranking hits<br>• **Figure S 3- S 10:** "NMR" & Mass Spectrum for Hit 88 (NCI 1576)<br>• **Figure S 11- S 18:** "NMR" & Mass Spectrum for Hit 130 (NCI 12849)<br>• **Figure S 19- S 25:** "NMR" & Mass Spectrum for Hit 85 (NCI 14040)<br>• **Figure S 26- S 32:** "NMR" & Mass Spectrum for Hit 112 (NCI 12415)<br>• **Figure S 33- S 38:** "NMR" & Mass Spectrum for Hit 141(NCI 12492)

- ***Table S1:*** *The structures of the (79) seventy-nine Aurora-A Kinase inhibitors collected from (ChEMBL), utilized in modeling.*

List for the structures of (79) Aurora-A Kinase inhibitors utilized in modeling and their reported Ki values (expressed in Nm-nanoMolar) collected from the European Bioinformatics Institute database (ChEMBL)

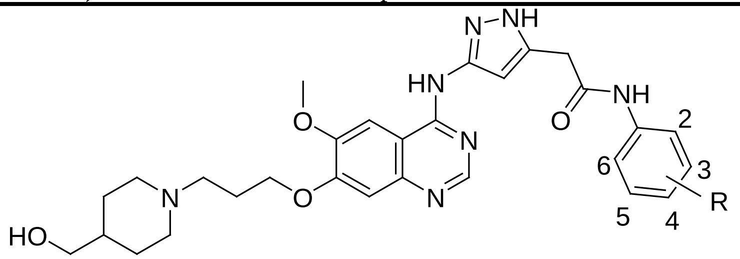

Compound	R	Ki (nM)	Reference
**1**	-H	1600	(Mortlock, et al., 2007)
**2**	2-F	1400	(Mortlock, et al., 2007)
**3**	3-F	450	(Mortlock, et al., 2007)
**4**	4-F	2000	(Mortlock, et al., 2007)
**5**	2,3-di-F	410	(Mortlock, et al., 2007)
**6**	3,5-di-F	230	(Mortlock, et al., 2007)
**7**	3-Cl	450	(Mortlock, et al., 2007)
**8**	3-CN	2600	(Mortlock, et al., 2007)
**9**	3-OH	4000	(Mortlock, et al., 2007)
**10**	3-OMe	1900	(Mortlock, et al., 2007)
**11**	3-CF_3	510	(Mortlock, et al., 2007)

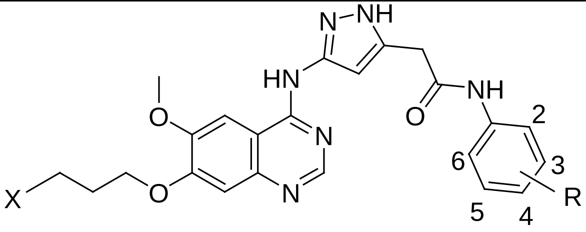

Compound	R	X	Ki (nM)	Reference
**12**	2,3-di-F	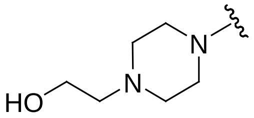	55	(Mortlock, et al., 2007)
**13**	2,3-di-F	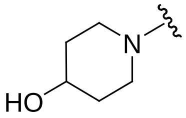	220	(Mortlock, et al., 2007)
**14**	2,3-di-F	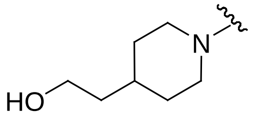	280	(Mortlock, et al., 2007)
**15**	3-F	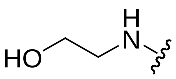	690	(Mortlock, et al., 2007)

Compound	R	X	Ki (nM)	Reference
**16**	3-F	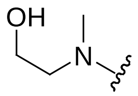	65	(Mortlock, et al., 2007)
**17**	3-F	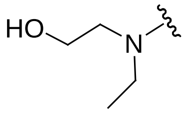	190	(Mortlock, et al., 2007)
**18**	3-F	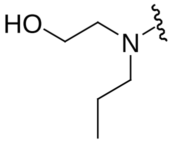	220	(Mortlock, et al., 2007)
**19**	2,3-di-F	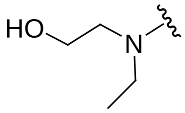	360	(Mortlock, et al., 2007)
**20**	2,3-di-F	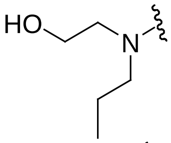	87	(Mortlock, et al., 2007)
**21**	2,3-di-F	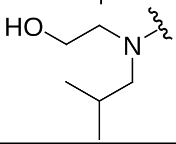	18	(Mortlock, et al., 2007)

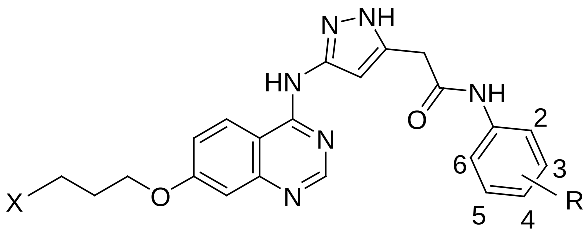

Compound	R	X	Ki (nM)	Reference
**22**	2,3-di-F	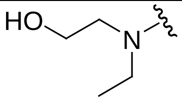	160	(Mortlock, et al., 2007)
**23**	2,3-di-F	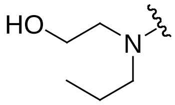	350	(Mortlock, et al., 2007)
**24**	3-F	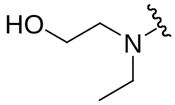	1400	(Mortlock, et al., 2007)
**25**	3-F	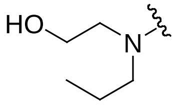	980	(Mortlock, et al., 2007)

Compound	Structure	Ki (nM)	Reference
**26**	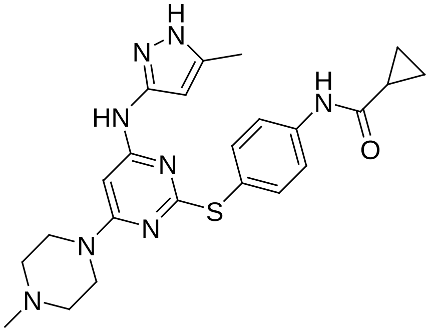	0.6	(Pollard and Mortimore, 2009)
**27**	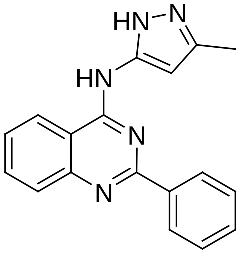	58	(Pollard and Mortimore, 2009)
**28**	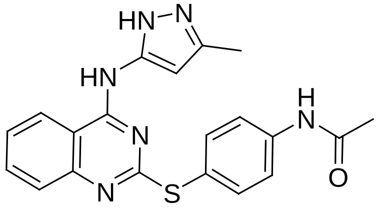	4	(Pollard and Mortimore, 2009)

Compound	Structure	Ki (nM)	Reference
**29**	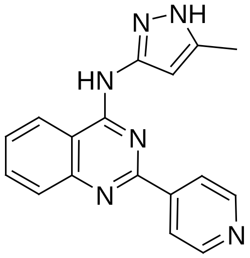	81	(Bebbington, et al., 2009)

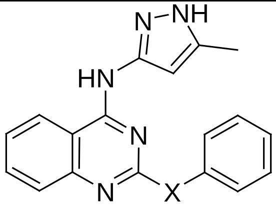

Compound	X	Ki (nM)	Reference
**30**	NH	24	(Bebbington, et al., 2009)
**31**	NMe	17	(Bebbington, et al., 2009)
**32**	O	36	(Bebbington, et al., 2009)
**33**	S	20	(Bebbington, et al., 2009)

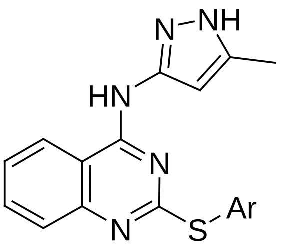

Compound	Ar	Ki (nM)	Reference
**34**	2-Cl-Ph	5	(Bebbington, et al., 2009)
**35**	3-Cl-Ph	4	(Bebbington, et al., 2009)
**36**	4-Cl-Ph	6	(Bebbington, et al., 2009)
**37**	2,3-Di-Cl-Ph	3	(Bebbington, et al., 2009)
**38**	2,4-Di-Cl-Ph	2	(Bebbington, et al., 2009)
**39**	2,6-Di-Cl-Ph	5	(Bebbington, et al., 2009)
**40**	3,4-Di-Cl-Ph	2	(Bebbington, et al., 2009)
**41**	2-OMe-Ph	24	(Bebbington, et al., 2009)
**42**	4-OMe-Ph	9	(Bebbington, et al., 2009)
**43**	3,4-Di-OMe-Ph	17	(Bebbington, et al., 2009)
**44**	2-Naphthyl	1	(Bebbington, et al., 2009)

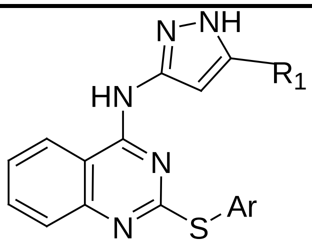

Compound	R1	Ar	Ki (nM)	Reference
**45**	Me	4-($NHSO_2Me$)-Ph	2	(Bebbington, et al., 2009)
**46**	Me	4-(NHC(O)OtBu)-Ph	9	(Bebbington, et al., 2009)
**47**	Me	4-(NHC(O)Et)-Ph	1	(Bebbington, et al., 2009)
**48**	H	4-(NHC(O)Me)-Ph	24	(Bebbington, et al., 2009)

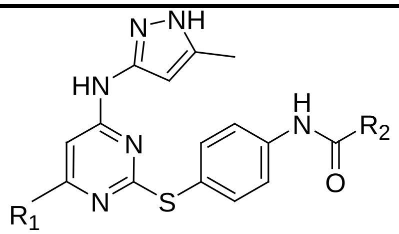

Compound	R1	R2	Ki (nM)	Reference
**49**	H	Me	86	(Bebbington, et al., 2009)
**50**	Me	Me	18	(Bebbington, et al., 2009)
**51**	Ph	Me	2.4	(Bebbington, et al., 2009)
**52**	Me	Et	5.1	(Bebbington, et al., 2009)
**53**	CyPr	Et	3.9	(Bebbington, et al., 2009)
**54**	tBu	Et	10	(Bebbington, et al., 2009)
**55**	3-Py	Et	3.9	(Bebbington, et al., 2009)
**56**	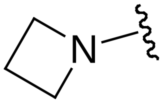	Et	2.4	(Bebbington, et al., 2009)
**57**	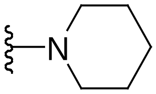	Et	1.6	(Bebbington, et al., 2009)
**58**	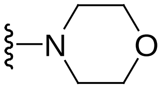	Et	1.7	(Bebbington, et al., 2009)
**59**	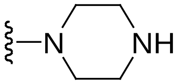	Et	3.7	(Bebbington, et al., 2009)
**60**	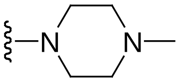	Et	1.6	(Bebbington, et al., 2009)
**61**	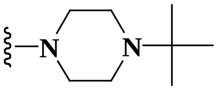	Et	1.3	(Bebbington, et al., 2009)

Compound	Structure	Ki (nM)	Reference
**62**	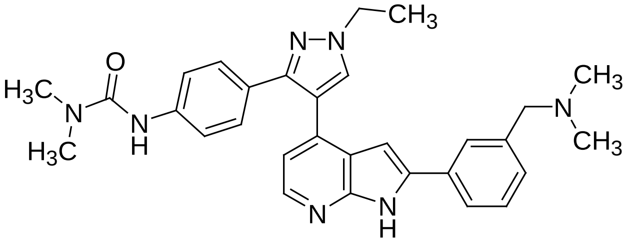	490	(Adams, et al., 2010)

**Compound**	**Structure**	**Ki (nM)**	**Reference**
**63**	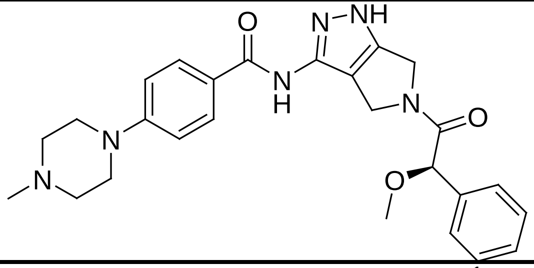	2.5	(Coumar, et al., 2008)

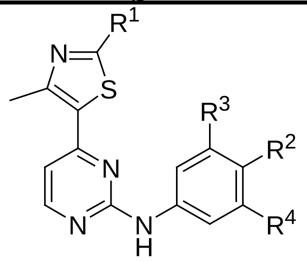

**Compound**	**R1**	**R2**	**R3**	**R4**	**Ki (nM)**	**Reference**
**64**	NH_2	H	NO_2	H	73.0	Wang, et al., 2010
**65**	Me	NMe_2	H	H	6.9	Wang, et al., 2010
**66**	Me	NHMe	H	H	18.0	Wang, et al., 2010
**67**	Me	NH_2	H	H	31.0	Wang, et al., 2010
**68**	Me	OMe	OMe	OMe	0.4	Wang, et al., 2010
**69**	NHMe	OMe	OMe	OMe	1.0	Wang, et al., 2010
**70**	NHEt	OMe	OMe	OMe	3.0	Wang, et al., 2010
**71**	NHEt	CH_2NHAc	H	H	5.0	Wang, et al., 2010
**72**	Me	morpholin-1-yl	H	H	4.0	Wang, et al., 2010
**73**	NH_2	morpholin-1-yl	H	H	8.0	Wang, et al., 2010
**74**	NHMe	morpholin-1-yl	H	H	19.0	Wang, et al., 2010
**75**	NHEt	morpholin-1-yl	H	H	14.0	Wang, et al., 2010
**76**	NH_2	4-acetylpiperazin-1-yl	H	H	7.6	Wang, et al., 2010
**77**	NHMe	4-acetylpiperazin-1-yl	H	H	12.0	Wang, et al., 2010
**78**	Me	4-acetylpiperazin-1-yl	H	H	8.2	Wang, et al., 2010
**79**	Me	4-(methylsulfonyl) piperazin-1-yl	H	H	9.2	Wang, et al., 2010

- ***Section S 1: LibDock Docking Engine and Scoring Settings***

LibDock enables rapid screening of combinatorial libraries where conformations of the ligands are aligned to polar and apolar receptor interactions sites (i.e., hotspots). Conformations can be either pre-calculated or generated on the fly. Since some of the output poses may have hydrogen atoms in close proximity to the receptor, a CHARMm minimization step can be optionally enabled to further optimize the docked poses.

LibDock docking follows the following steps: (1) Remove hydrogen atoms, (2) Rank ligand conformations and prune by solvent accessible surface area (SASA), (3) find hotspots using a grid that is placed into the binding site and using polar and non-polar probes. The numbers of hotspots are pruned by clustering to a user defined value, (4) Dock ligand poses by aligning to binding site hotspots. This is performed by using triplets (i.e., three ligand atoms are aligned to three receptor hotspots). (5) Poses which result in protein clashes are removed. (6) A final BFGS pose optimization stage is performed using a simple pair-wise score (similar to Piecewise Linear Potential). The top scoring ligand poses are retained (7) Hydrogen atoms are added back to the docked ligands. (8) Optionally, CHARMm minimization can be carried out to reduce steric clashes caused by added hydrogen atoms.

The following LibDock parameters were implemented in the presented project: (1) Prior to docking, the DiscoveryStudio 2.5.5 module CAT-CONFIRM was used to generate a maximum of 255 conformers (not exceeding an energy threshold of 20 kcal/mol from the most stable conformer) for each ligand employing "CAESAR" conformation generation option. (2) A binding site sphere of 12.2 Å radius surrounding the centre of the co-crystallized ligand was used to define the binding site. (3) The maximum number of binding site hotspots (polar and apolar) was set to 100. (4) The ligand-to-hotspots matching RMSD tolerance value was set to 0.25Å. (5) The maximum number of poses saved for each ligand during hotspots matching before final pose

minimization = 100. (6) Maximum number of poses to be saved for each ligand in the binding pocket = 100. (7) Minimum LibDock score (poses below this score are not reported) = 100. (8) Maximum number of rigid body minimization steps during the final pose optimization (using BFGS method) = 50. (9) Maximum number of steric clashes allowed before the pose-hotspot alignment is terminated (specified as a fraction of the heavy atom count) = 0.1.

(10) Maximum value for nonpolar solvent accessible surface area for a particular pose to be reported as successful = 15.0Å^2. (11) Maximum value for polar solvent accessible solvent area for a particular pose to be reported as successful = 5.0Å^2. (12) No final ligand minimization was implemented (i.e., in the binding pocket).

- ***Section S 2: Scoring of Docked Ligand Poses.***

Highest ranking docked conformers/poses generated by LibDock were scored using 7 scoring functions: Jain (Jain, 1996), LigScore1, LigScore2 (Venkatachalam, et al., 2003), PLP1, PLP2 (Gehlhaar, et al., 1999), PMF and PMF04 (Muegge and Martin 1999; Muegge, 2002).

LigScore1 and LigScore2 scores were calculated employing CFF force field (version 1.02) and using grid-based energies with a grid extension of 7.5 Å across the binding site. PMF scores were calculated employing cutoff distances of 12.0 Å for carbon-carbon interactions and other atomic interactions, while PMF04 scores were calculated employing cutoff values of 6.0 and 9.0Å for carbon-carbon interactions and other atomic interactions, respectively.

- ***Section S 3: Genetic Algorithm Implementation in dbCICA Modeling***

The GA toolbox within MATLAB (Version R2007a) was adapted by implementing the following four basic components: the creation function, cross-over function, mutation function, and fitness function. The creation function randomly generates a population of chromosomes of a

predefined size (number of summed contacts columns, as mentioned in steps 5 and 6 in the section of Docking-Based Comparative Molecular Contacts Analysis (dbCICA) under Materials and Methods) in which every chromosome encodes for certain possible column summation model. Chromosomes differ from one another by the set of summed columns and their weights.

Crossover children are the offspring created by selecting vector entries (i.e., genes) from a pair of individual chromosomes in the first generation and combining them to form two complementary children, while mutation children are those created via applying random changes to corresponding parents, i.e., each single parent chromosome is mutated to give a single child by randomly replacing selected gene in the parent chromosome with another from the chromosome population. Each chromosome is associated with a fitness value that reflects how good the summation of its encoded genes compares to other chromosomes. The fitness functions in dbCICA can be the correlation coefficient (r^2), leave-one-out r^2, or K-fold r^2.

In this project (dbCICA of Aurora-A Kinase) we implemented a 5-fold r^2 as fitness criterion. In this procedure, each chromosome is ranked as follows: The training set is divided into two subsets: fit and test subsets. The test subset is randomly selected to represent *ca.* 20% of the training compounds. This procedure is repeated over 5 cycles; accordingly, 5 test subsets with their complementary fit subsets are selected for each chromosome (i.e., column summation model).

The 5 test subsets should cover *ca.* 100% of the training compounds by avoiding selecting the same compound in more than one test subset. The fit sets are then utilized to generate 5 sub-models employing the same chromosome. The resulting sub-models are then utilized to predict the bioactivities of the corresponding testing subsets. Finally, the predicted values of all 5 test subsets are correlated with their experimental counterparts to determine corresponding 5-fold r^2 .

The best column-summation model is selected as representative db-CICA model. The fitness function in the current db-CICA modelling project was 5-fold r^2.

The following parameters were implemented for GA search of best models: (1) Size of chromosome population = 100, (2) Rate of mating (crossover fraction): 80% (3) Elite count = 1 (4) Maximum number of generations which is needed to exit from GA iteration cycles and completion of the algorithm = 1000.

Based on these settings, the numbers of each type of children in the offspring generation is as follows: There is 1 elite child (corresponding to the individual in the parents' generation with the best fitness value), and there are 199 individual children other than the elite child. The algorithm rounds 0.8 (crossover fraction) x 199 = 159.2 to 159 to get the number of crossover children and the remaining 40 (i.e., 199-159) are the mutation population. The elite child is passed to the offspring population without alteration.

- ***Section S 4: Receiver-operating characteristic (ROC) curve analysis***

The Testing Set: The classification power of the resulting pharmacophores was validated using receiver-operating characteristic (ROC) curve analysis by testing the ability of a particular pharmacophore to selectively capture diverse Aurora-A kinase inhibitors from large list of inactive compounds. The testing set implemented in this project was entirely composed of experimentally validated active and inactive Aurora-A kinase inhibitors extracted from the European Bioinformatics Institute database (ChEMBL, https://www.ebi.ac.uk/chembl). It included 86 experimentally-validated active compounds (anti-Aurora-A Kinase with Ki values ≤10 nM) & 248 less-active compounds (anti-Aurora-A Kinase with Ki values > 500 nM considered as decoy list). To insure that active testing compounds closely resemble the diversity

of less-active members we computed top three principal components based on 12 physicochemical descriptors (i.e., LogP, molecular weight, hydrogen bond donors and acceptors, rotatable bonds, rings, aromatic rings, fractional polar surface area surface area, polar surface area and number of fragments) for active testing compounds and compared them with corresponding principal components calculated for the inactive testing inhibitors in each case. Below figure shows three-dimensional plot of the principal components representing active and inactive testing compounds testing set.

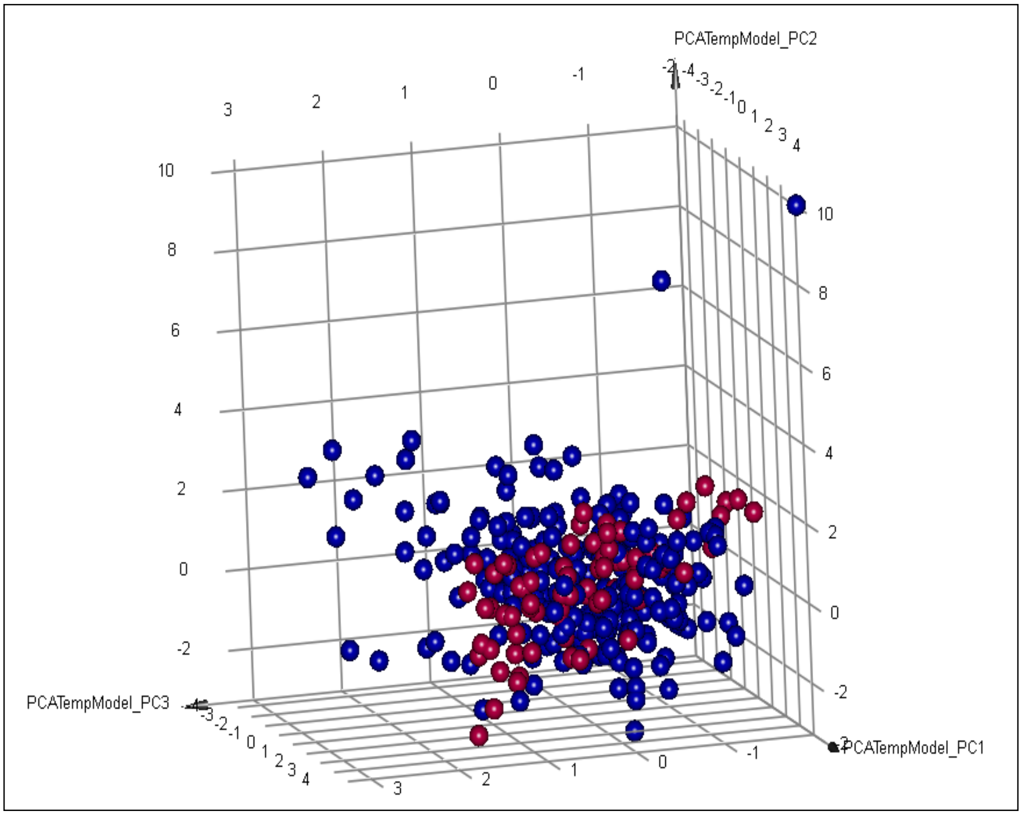

Figure S 1: Three-dimensional plot showing three main principal components calculated for the Testing Set (based on 12 physicochemical descriptors, see text). Amber spheres (●) represent active compounds (Ki ≤ 10) while blue spheres (●) represent inactive compounds (Ki > 500) as enlisted in ChEMBL database.

Conformational ensembles were generated for the testing set using "CESEAR" conformation generation option implemented in DiscoveryStudio 2.5.5. The results are presented in the form of ROC curves. A ROC curve is plotted by considering the highest score (fit value against the

tested pharmacophore) of an active molecule as the first threshold then counting the number of decoy compounds within this cut-off value.

The corresponding sensitivity (SE, also known as True Positive Rate) and specificity (SP, also known as True Negative Rate) are calculated using equation 1 and equation 2, respectively, and plotted. This process is repeated using the active molecule possessing the second highest score and so on, until the scores of all active compounds are considered as selection cut-off values (Triballeau, et al., 2006 and Kirchmair, et al., 2008).

$$Se = \frac{Number\ of\ Selected\ Actives}{Total\ Number\ of\ Actives} = \frac{TP}{TP + FN} \quad \ldots\ldots\ldots\ldots\ldots\ldots\ldots (1)$$

$$Sp = \frac{Number\ of\ Discarded\ Inactives}{Total\ Number\ of\ Inactives} = \frac{TN}{TN + FP} \quad \ldots\ldots\ldots\ldots\ldots (2)$$

Where, TP (true positive) is the number of active compounds that are captured by the pharmacophore under concern, FN (false negative) is the number of active compounds discarded from the hits list by the virtual screening method, TN (true negative) is the number of discarded decoys, while FP (false positive) is the number of captured decoys (presumably inactive) (Irwin and Shoichet, 2005; Triballeau et al., 2006; Kirchmair et al., 2008).

If all molecules scored by a virtual screening (VS) protocol with sufficient discriminatory power are ranked according to their score (i.e., fit values), starting with the best-scored molecule and ending with the molecule that got the lowest score, most of the actives will have a higher score than the decoys. Since some of the actives will be scored lower than decoys, an overlap between the distribution of active molecules and decoys will occur, which will lead to the prediction of

false positives and false negatives. (Irwin and Shoichet, 2005; Triballeau et al., 2006; Kirchmair et al., 2008). The selection of one score value as a threshold strongly influences the ratio of actives to decoys and therefore the validation of a VS method. The ROC curve method avoids the selection of a threshold by considering all SE and SP pairs for each score threshold (Irwin and Shoichet, 2005; Triballeau et al., 2006; Kirchmair et al., 2008). A ROC curve is plotted by setting the score of the active molecule as the first threshold. Afterwards, the number of decoys within this cutoff is counted and the corresponding SE and SP pair is calculated. This calculation is repeated for the active molecule with the second highest score and so forth, until the scores of all actives are considered as selection thresholds.

In practice, the ROC curve for a set of actives and inactive decoys with randomly distributed scores tends towards the SE = 1-SP line asymptotically with increasing number of actives and decoys (Triballeau, et al., 2005). The success of particular virtual screening workflow depending on ROC analysis evaluation can be provided as follow:

1) Area under the ROC curve (AUC): optimal ROC curve has a value of 1 and random distribution with 0.5. Any virtual screening that performs better than a random discrimination of actives and inactives get an AUC value between 0.5 and 1, whereas an AUC value lower than 0.5 represents the unfavourable case of a virtual screening method that has a higher probability to assign the best scores to decoys than to actives (Irwin and Shoichet, 2005; Triballeau, et al., 2006; Taha, 2012).
2) Overall accuracy (ACC): describes the percentage of compounds that were correctly classified by the screening protocol (equation 3). Testing compounds are assigned a binary score value of zero (compound not captured) or one (compound captured) (Triballeau, et al., 2006).

$$ACC = \frac{TP + TN}{N} = \frac{A}{N}.Se + \left(1 - \frac{A}{N}\right).Sp \quad ……….. (3)$$

Where, N is the number of all compounds in the testing database, A is the number of true actives in the testing database.

3) Overall true negative rate (TNR) or overall specificity (SP): that describes the fraction percentage of discarded inactive by the virtual screening tool. Discarded inactive test compounds are assigned a binary score value of zero (compound not captured) or one (compound captured) regardless to their individual fit values (Jacobsson et al., 2003; Irwin and Shoichet, 2005; Triballeau et al., 2006; Kirchmair et al., 2008, Taha, 2012).

4) Overall true positive rate (TPR) or overall sensitivity (SE): describes the fraction percentage of captured actives from the total number of actives. Active test compounds are assigned a binary score value of zero (compound not captured) or one (compound captured) regardless to their individual fit values (Jacobsson et al., 2003; Irwin and Shoichet, 2005; Triballeau et al., 2006; Kirchmair et al., 2008, Taha, 2012).

- ***Section S5: Steric Refinement of pharmacophores***

Based on ROC results **Hypo(SB-1)** model had better behavior over **Hypo(SB-2),** as detailed in results section. In order to improve the classification properties of **Hypo(SB-1)** model, it was complemented with exclusion spheres by employing HIPHOP-REFINE module of DiscoveryStudio 2.5.5 (Khanfar and Taha, 2013). HIPHOP-REFINE identifies spaces occupied by the conformations of inactive compounds and free from conformations of active ones. These areas are filled with exclusion volumes to represent the steric constrains of the binding pocket (Hahn, 1997; Taha, et al., 2011; Khanfar and Taha, 2013). A subset of 32 training compounds

was carefully selected from the molecules in shown below in **Table S 2** for HIPHOP-REFINE modelling or construction of appropriate exclusion regions around **Hypo(SB-1).**

The Principal and Maximum Omitted Features (MaxOmitFeat) parameters are used to define how many molecules fit the selected pharmacophore hypothesis (partially or completely) for steric refinement purposes. Active compounds are assigned MaxOmitFeat parameter of zero and Principal value of 2 to direct the software to fit all their chemical moieties against all the pharmacophoric features of the particular hypothesis. However, inactive compounds are allowed to miss one or two features by assigning them a MaxOmitFeat of 1 or 2, respectively. Moreover, inactives were assigned Principal value of zero to indicate their inferior bioactivities. However, intermediate active compounds are normally assigned a principal value of 1 and a MaxOmitFeat of zero or 1 in accordance with the number of features the compound loses, to indicate their intermediate status (Taha, et al., 2011).

In this project it was decided to consider the Ki value of 510 nM as an arbitrary activity/inactivity threshold, such that compounds with values equal to or more than 510 nM considered “inactives” with Principal value of zero, and were carefully evaluated to assess whether their lower potencies are attributable to missing one or more pharmacophoric features (MaxOmitFeat = 1 or 2), or only related to steric clashes within the binding pocket (MaxOmitFeat = 0).

However, compounds of Ki values ranging from 5 nM to less than 510 nM were considered moderately active and were assigned a principal value of 1 and MaxOmitFeat of 1 or zero (according to their number of missed feature). Compounds of Ki values less than 5.0 nM were considered active, and were assigned Principal value of 2, and MaxOmitFeat of zero. The conformational spaces of training lists were generated using "BEST" conformation generation option in DiscoveryStudio 2.5.5. The training compounds employed for steric refinement of the

generated pharmacophore **Hypo(SB-1),** and their corresponding Principal and MaxOmitFeat parameters are sown below in **Table S 2.**

HIPHOP-REFINE was configured to permit a maximum of 100 exclusion spheres to be added to pharmacophoric hypothesis of **Hypo(SB-1).** The HIPHOP-REFINE process resulted in adding 93 exclusion volumes to **Hypo(SB-1)**, and the sterically refined pharmacophore was named **Refined-Hypo (SB-1).**

Table S 2: Refinement list for steric refinement of **Hypo(SB-1)**

**Compound**[a]	**Ki (nM)**	**Principal value**	**MaxOmitFeat**[b]
**1**	1,600	0	2
**2**	1,400	0	2
**4**	2,000	0	2
**8**	2,600	0	2
**9**	4,000	0	0
**10**	1,900	0	2
**11**	510	0	2
**15**	690	0	2
**20**	87	1	1
**24**	1,400	0	2
**25**	980	0	2
**26**	0.6	2	0
**28**	4	2	0
**34**	5	1	1
**39**	5	1	1
**42**	9	1	0
**43**	17	1	0
**45**	2	2	0
**46**	9	1	0
**47**	1	2	0
**49**	86	1	0
**50**	18	1	0
**52**	5.1	1	0
**54**	10	1	0
**57**	1.6	2	0
**58**	1.7	2	0
**60**	1.6	2	0
**61**	1.3	2	0
**66**	18	1	1
**71**	5	1	1
**78**	8.2	1	1
**79**	9.2	1	1

[a]Compounds' numbers are as in **Table S3**
[b]MaxOmitFeat: Maximum omitted features.

Table S3: The 75 high-ranking hits and their anti-Aurora-A kinase inhibition% at 10 μM using Z'-LYTE kinase assay, sum of their critical contacts and their predicted activity. These high-ranking hits captured by **Refined-Hypo(SB-1)** -3D search query- derived from **Hypo(SB-1)** pharmacophore were docked into (3w2c) using the docking-scoring settings of **(SB-1)** and their docked poses were analyzed to identify their critical binding contacts (marked by dbCICA model **Table 2.**) were used to predict their K_i values by substituting the sum of binding contacts in the respective dbCICA-regression equations (**Table 1.**). Steps of activity prediction was also employed using docking/scoring settings of **(SB-2)** dbCICA model to assess the similarity extent in "predicted activity" of both **(SB-2)** model and the better performing **(SB-1)** model

**Hit**[a]	**NCI Code**	**SB-1**		**SB-2**		**% Inhibition at 10 μM**
		**Contact atoms Summation**[b]	**Predicted Ki (nM)**[c]	**Contact atoms Summation**[b]	**Predicted Ki (nM)**[c]	
**80**	**19024**	15	0.27	14	6.96	20
**81**	**34607**	15	0.27	14	6.96	3
**82**	**1987**	14	0.58	12	18.72	5
**83**	**22650**	14	0.58	13	11.41	7
**84**	**4293**	14	0.58	13	11.41	17
**85***	**14040***	13	1.20	13	11.41	**56**
**86**	**22651**	13	1.20	13	11.41	12
**87**	**24666**	13	1.20	11	30.71	-4
**88**	**1576**	12	2.51	14	6.96	**75**
**89**	**18100**	12	2.51	13	11.41	2
**90**	**23953**	12	2.51	13	11.41	-2
**91**	**33654**	12	2.51	13	11.41	-2
**92**	**34311**	12	2.51	12	18.72	3
**93**	**34595**	12	2.51	13	11.41	14
**94**	**35036**	12	2.51	10	50.37	-2
**95**	**4356**	12	2.51	12	18.72	30
**96**	**4721**	12	2.51	11	30.71	0
**97**	**7506**	12	2.51	12	18.72	-4
**98**	**10637**	11	5.22	9	82.61	-5
**99**	**11196**	11	5.22	12	18.72	5

[a]Hits are as in **Figure S 3**. [b]Contacts summations according to corresponding dbCICA model (Tables 1 and 2). [c]Predicted Ki (nM) by substituting the number of contacts of each docked compound in the regression equation of the corresponding dbCICA model. *Underlined hits with highest Inhibition%

Table S3: The 75 high-ranking hits and their anti-Aurora-A kinase inhibition% at 10 µM using Z'-LYTE kinase assay, sum of their critical contacts and their predicted activity. These high-ranking hits captured by **Refined-Hypo(SB-1)** -3D search query- derived from **Hypo(SB-1)** pharmacophore were docked into (3w2c) using the docking-scoring settings of **(SB-1)** and their docked poses were analyzed to identify their critical binding contacts (marked by dbCICA model **Table 2.**) were used to predict their K_i values by substituting the sum of binding contacts in the respective dbCICA-regression equations (**Table 1.**). Steps of activity prediction was also employed using docking/scoring settings of **(SB-2)** dbCICA model to assess the similarity extent in "predicted activity" of both **(SB-2)** model and the better performing **(SB-1)** model

**Hit**[a]	**NCI Code**	**SB-1**		**SB-2**		**% Inhibition at 10 µM**
		**Contact atoms Summation**[b]	**Predicted Ki (nM)**[c]	**Contac atoms Summation**[b]	**Predicted Ki (nM)**[c]	
**100**	12847	11	5.22	11	30.71	7
**101**	17288	11	5.22	13	11.41	6
**102**	18099	11	5.22	12	18.72	6
**103**	22645	11	5.22	12	18.72	12
**104**	23413	11	5.22	12	18.72	23
**105**	33564	11	5.22	13	11.41	1
**106**	34873	11	5.22	12	18.72	6
**107**	4354	11	5.22	12	18.72	25
**108**	6919	11	5.22	11	30.71	-7
**109**	7501	11	5.22	12	18.72	-142
**110**	9293	11	5.22	10	50.37	3
**111**	10515	10	10.87	13	11.41	1
**112**	12415	10	10.87	14	6.96	**55**
**113**	14341	10	10.87	12	18.72	8
**114**	22676	10	10.87	10	50.37	4
**115**	22677	10	10.87	11	30.71	-1
**116**	23825	10	10.87	13	11.41	21
**117**	26690	10	10.87	15	4.24	7

[a]Hits are as in **Figure S 3**. [b]Contacts summations according to corresponding dbCICA model (Tables 1 and 2). [c]Predicted Ki (nM) by substituting the number of contacts of each docked compound in the regression equation of the corresponding dbCICA model. *Underlined hits with highest Inhibition%

Table S3: The 75 high-ranking hits and their anti-Aurora-A kinase inhibition% at 10 µM using Z'-LYTE kinase assay, sum of their critical contacts and their predicted activity. These high-ranking hits captured by **Refined-Hypo(SB-1)** -3D search query- derived from **Hypo(SB-1)** pharmacophore were docked into (3w2c) using the docking-scoring settings of **(SB-1)** and their docked poses were analyzed to identify their critical binding contacts (marked by dbCICA model **Table 2.**) were used to predict their K_i values by substituting the sum of binding contacts in the respective dbCICA-regression equations (**Table 1.**). Steps of activity prediction was also employed using docking/scoring settings of **(SB-2)** dbCICA model to assess the similarity extent in "predicted activity" of both **(SB-2)** model and the better performing **(SB-1)** model

Hit[a]	NCI Code	SB-1		SB-2		% Inhibition at 10 µM
		Contact atoms Summation[b]	Predicted Ki (nM)[c]	Contact atoms Summation[b]	Predicted Ki (nM)[c]	
**118**	29057	10	10.87	14	6.96	7
**119**	31475	10	10.87	13	11.41	-3
**120**	31937	10	10.87	10	50.37	-2
**121**	3343	10	10.87	11	30.71	-2
**122**	33550	10	10.87	13	11.41	-1
**123**	34304	10	10.87	13	11.41	4
**124**	34692	10	10.87	14	6.96	-2
**125**	6807	10	10.87	14	6.96	-1
**126**	6848	10	10.87	12	18.72	-2
**127**	10868	9	22.62	13	11.41	0
**128**	11191	9	22.62	13	11.41	-5
**129**	12840	9	22.62	13	11.41	3
**130**	**12849**	9	22.62	13	11.41	**86**
**131**	23575	9	22.62	12	18.72	3
**132**	26084	9	22.62	13	11.41	22
**133**	28316	9	22.62	12	18.72	3
**134**	31011	9	22.62	13	11.41	8
**135**	3289	9	22.62	13	11.41	7
**136**	5769	9	22.62	13	11.41	5
**137**	6888	9	22.62	12	18.72	-30

[a]Hits are as in **Figure S 3**. [b]Contacts summations according to corresponding dbCICA model (Tables 1 and 2). [c]Predicted Ki (nM) by substituting the number of contacts of each docked compound in the regression equation of the corresponding dbCICA model. *Underlined hits with highest Inhibition%

Table S3: The 75 high-ranking hits and their anti-Aurora-A kinase inhibition% at 10 µM using Z'-LYTE kinase assay, sum of their critical contacts and their predicted activity. These high-ranking hits captured by **Refined-Hypo(SB-1)** -3D search query- derived from **Hypo(SB-1)** pharmacophore were docked into (3w2c) using the docking-scoring settings of **(SB-1)** and their docked poses were analyzed to identify their critical binding contacts (marked by dbCICA model **Table 2.**) were used to predict their K_i values by substituting the sum of binding contacts in the respective dbCICA-regression equations (**Table 1.**). Steps of activity prediction was also employed using docking/scoring settings of **(SB-2)** dbCICA model to assess the similarity extent in "predicted activity" of both **(SB-2)** model and the better performing **(SB-1)** model

Hit[a]	NCI Code	SB-1		SB-2		% Inhibition at 10 µM
		Contact atoms Summation[b]	Predicted Ki (nM)[c]	Contact atoms Summation[b]	Predicted Ki (nM)[c]	
**138**	6924	8	47.07	13	11.41	-1
**139**	10188	8	47.07	13	11.41	6
**140**	11193	8	47.07	12	18.72	-7
**141**	**<u>12492</u>**	<u>8</u>	<u>47.07</u>	<u>11</u>	<u>30.71</u>	**<u>51</u>**
**142**	12990	8	47.07	13	11.41	-3
**<u>143</u>**	26037	8	47.07	11	30.71	-72
**144**	32263	8	47.07	13	11.41	0
**145**	33974	8	47.07	12	18.72	0
**146**	34870	8	47.07	13	11.41	4
**147**	4355	8	47.07	12	18.72	14
**148**	7959	8	47.07	13	11.41	-3
**149**	7960	8	47.07	13	11.41	-1
**150**	8793	8	47.07	13	11.41	-3
**151**	11155	7	97.94	13	11.41	-7
**152**	21194	7	97.94	10	50.37	1
**153**	3021	7	97.94	13	11.41	-6
**154**	34688	7	97.94	13	11.41	-2

[a]Hits are as in **Figure S 3**. [b]Contacts summations according to corresponding dbCICA model (Tables 1 and 2). [c]Predicted Ki (nM) by substituting the number of contacts of each docked compound in the regression equation of the corresponding dbCICA model. *Underlined hits with highest Inhibition%

Figure S 2. The chemical structures of the tested highest-ranking hits.

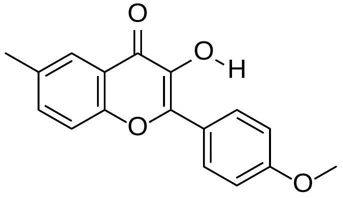	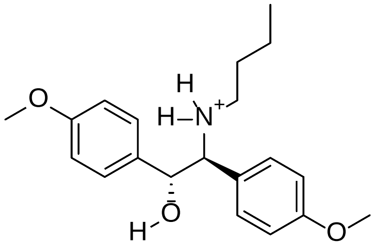	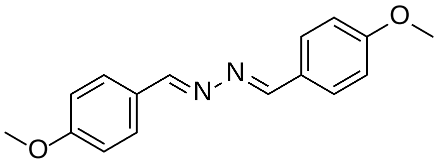
**80 (NCI 19024)**	**81 (NCI 34607)**	**82 (NCI 1987)**
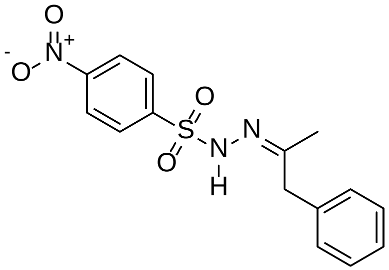	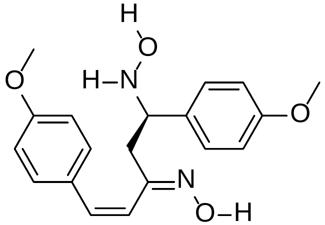	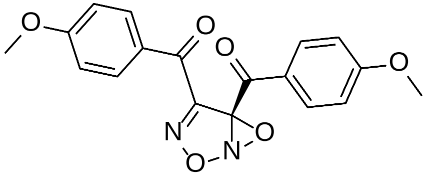
**83 (NCI 22650)**	**84 (NCI 4293)**	**85 (NCI 14040)**
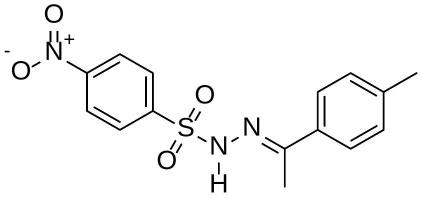	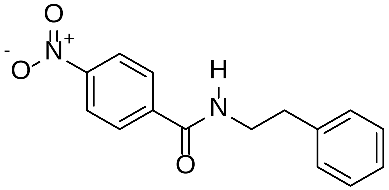	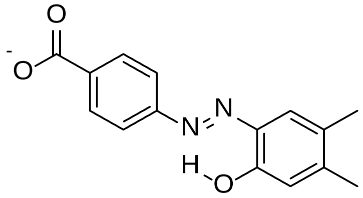
**86 (NCI 22651)**	**87 (NCI 24666)**	**88 (NCI 1576)**

Figure S 2.*(Continued)* - The chemical structures of the tested highest-ranking hits.

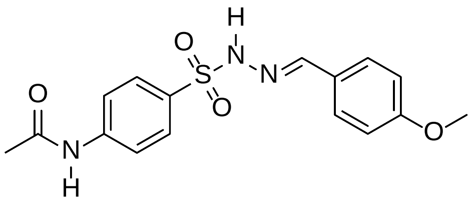	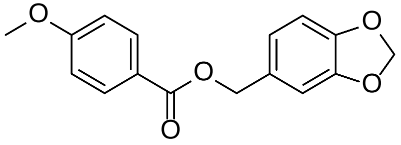	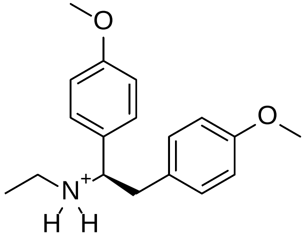
**89 (NCI 18100)**	**90 (NCI 23953)**	**91 (NCI 33654)**
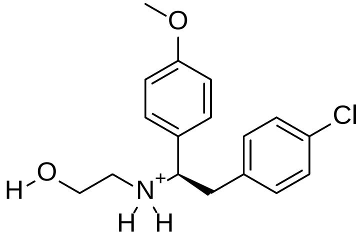	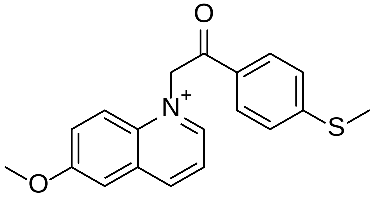	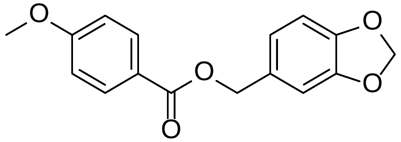
**92 (NCI 34311)**	**93 (NCI 34595)**	**94 (NCI 35036)**
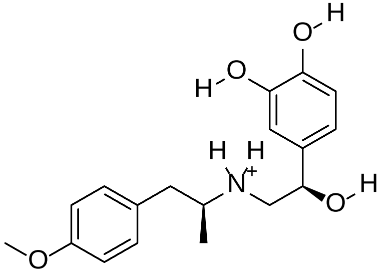	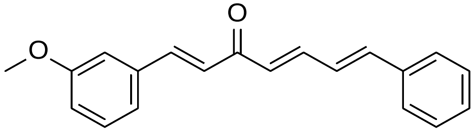	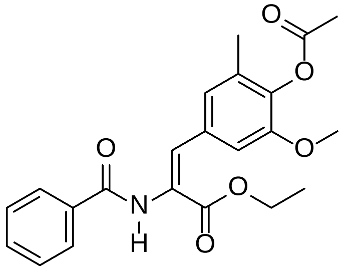
**95 (NCI 4356)**	**96 (NCI 4721)**	**97 (NCI 7506)**

Figure S 2. ***(Continued)*****-** The chemical structures of the tested highest-ranking hits.

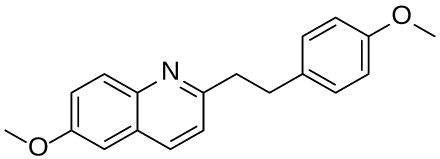

98 (NCI 10637)

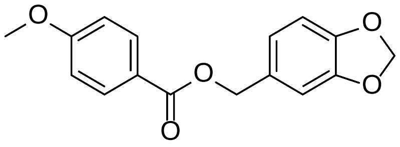

99 (NCI 11196)

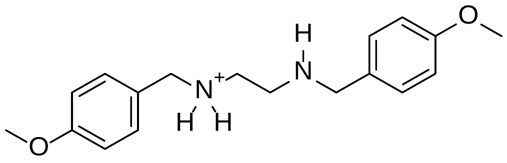

100 (NCI 12847)

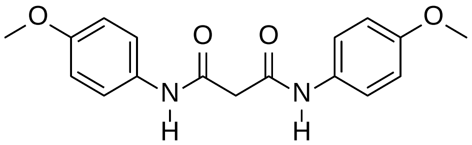

101 (NCI 17288)

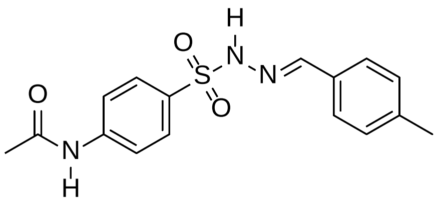

102 (NCI 18099)

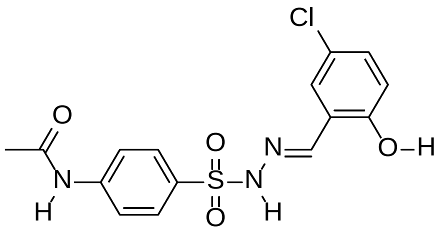

103(NCI 22645)

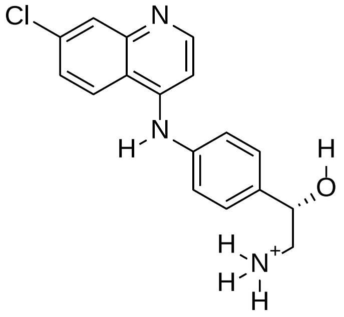

104(NCI 23413)

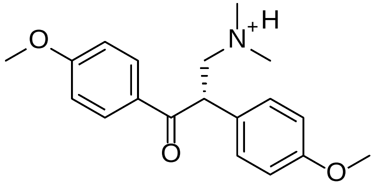

105 (NCI 33564)

Figure S 2. ***(Continued)*- The chemical structures of the tested highest-ranking hits.**

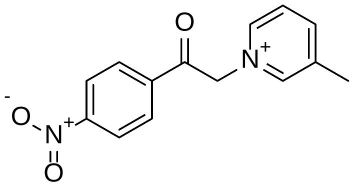

106 (NCI 34873)

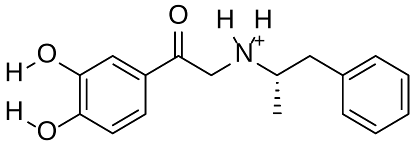

107 (NCI 4354)

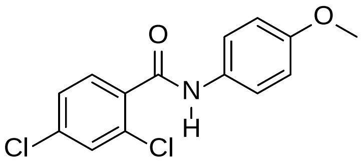

108 (NCI 6919)

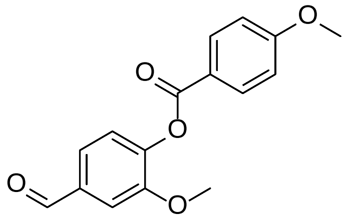

109 (NCI 7501)

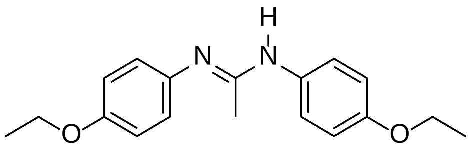

110 (NCI 9293)

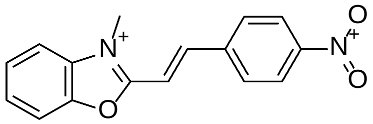

111 (NCI 10515)

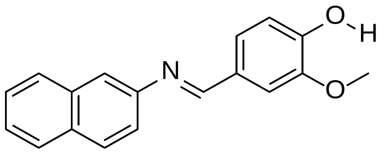

112 (NCI 12415)

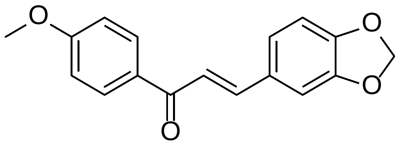

113 (NCI 14341)

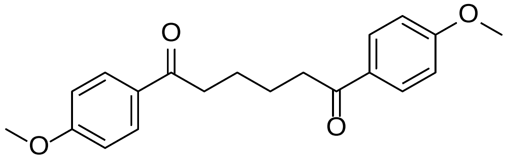

114 (NCI 22676)

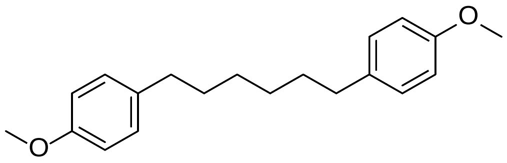

115 (NCI 22677)

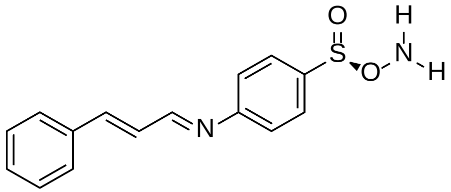

116 (NCI 23825)

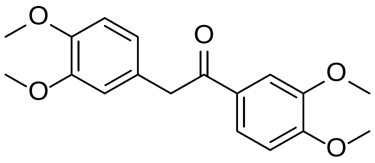

117 (NCI 26690)

Figure S 2. ***(Continued)*****- The chemical structures of the tested highest-ranking hits.**

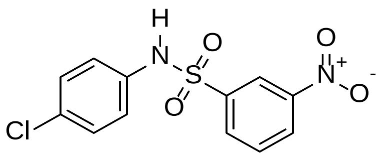

118 (NCI 29057)

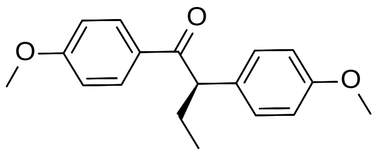

119 (NCI 31475)

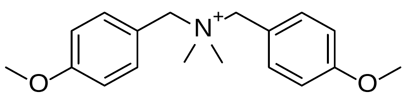

120 (NCI 31937)

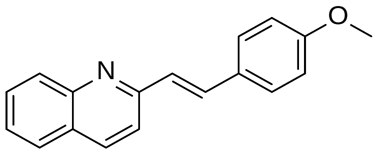

121 (NCI 3343)

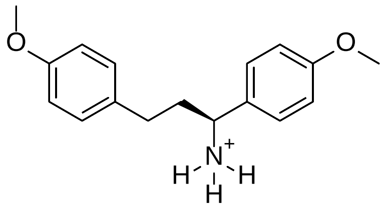

122 (NCI 33550)

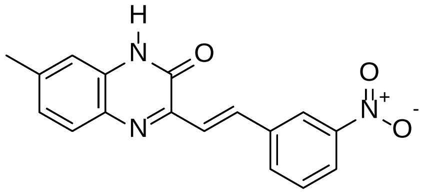

123 (NCI 34304)

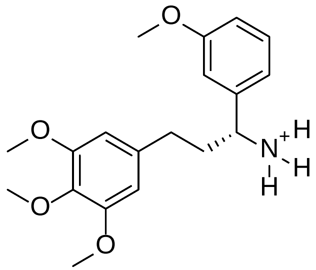

124 (NCI 34692)

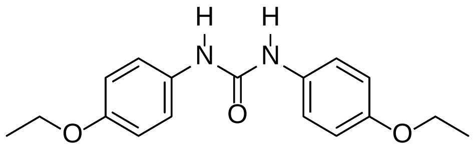

125 (NCI 6807)

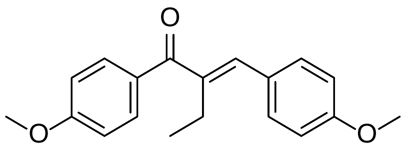

126 (NCI 6848)

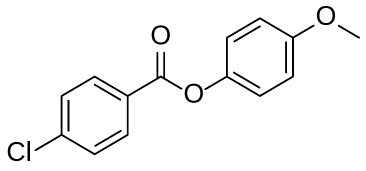

127 (NCI 10868)

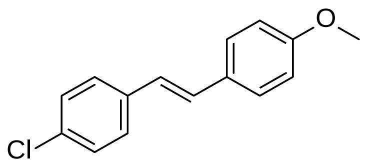

128 (NCI 11191)

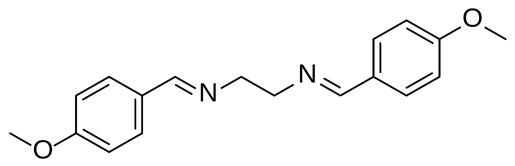

129 (NCI 12840)

Figure S 2. ***(Continued)-*** **The chemical structures of the tested highest-ranking hits.**

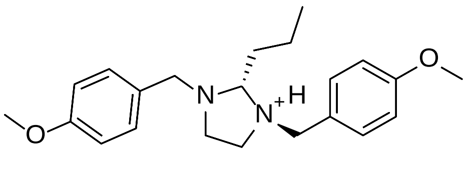

130 (NCI 12849)

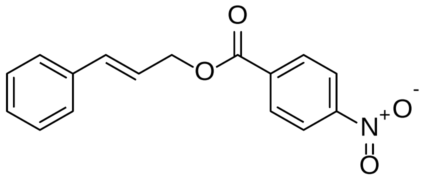

131 (NCI 23575)

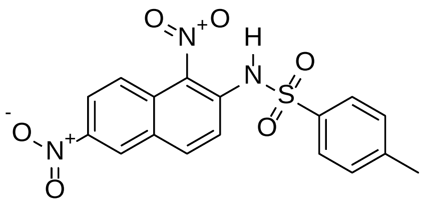

132 (NCI 26084)

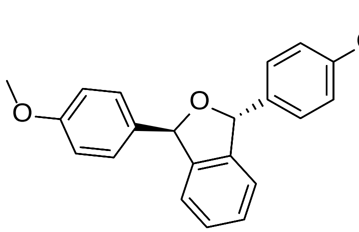

133 (NCI 28316)

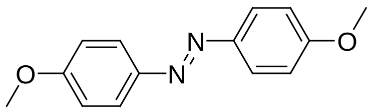

134 (NCI 31011)

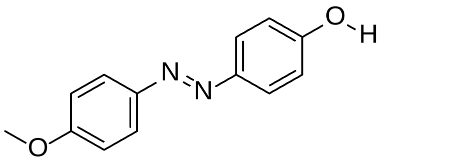

135 (NCI 3289)

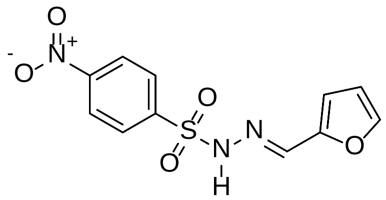

136 (NCI 5769)

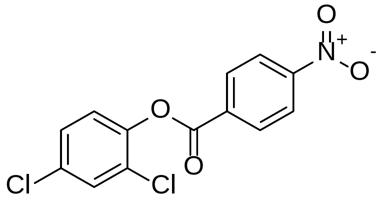

137 (NCI 6888)

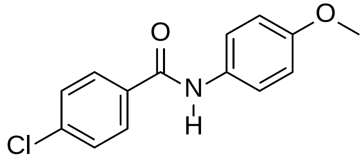

138 (NCI 6924)

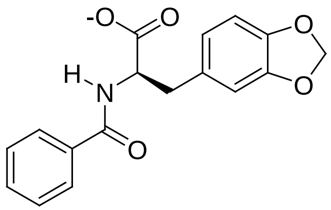

139 (NCI 10188)

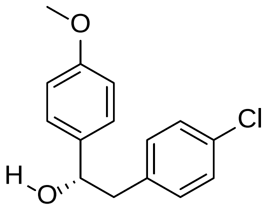

140 (NCI 11193)

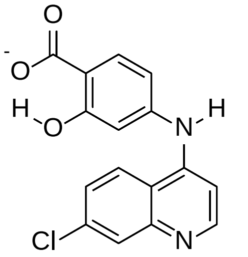

141 (NCI 12492)

Figure S 2. ***(Continued)*****- The chemical structures of the tested highest-ranking hits.**

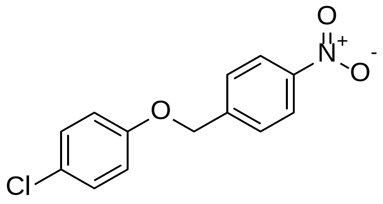

142 (NCI 12990)

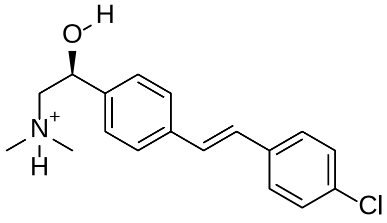

143 (NCI 26037)

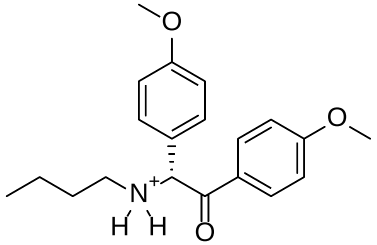

144 (NCI 32263)

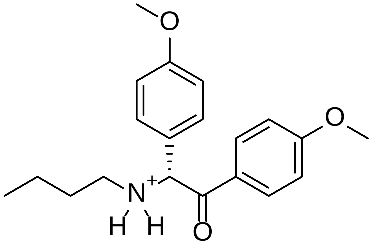

144 (NCI 32263)

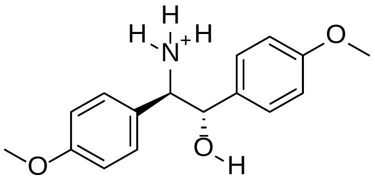

145 (NCI 33974)

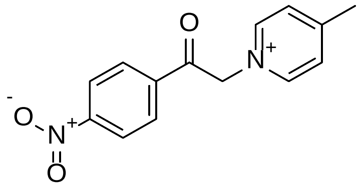

146 (NCI 34870)

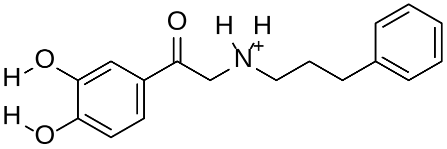

147 (NCI 4355)

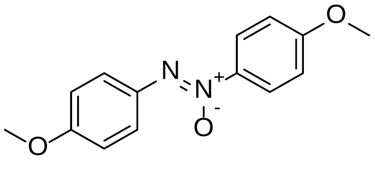

148 (NCI 7959)

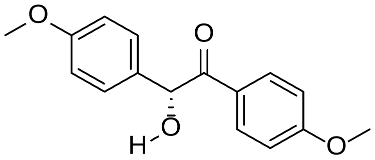

149 (NCI 7960)

Figure S 2. ***(Continued)*- The chemical structures of the tested highest-ranking hits.**

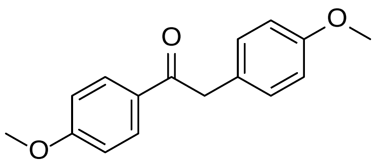

150 (NCI 8793)

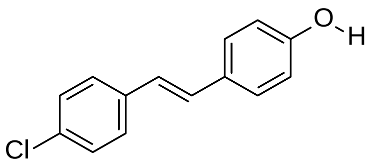

151 (NCI 11155)

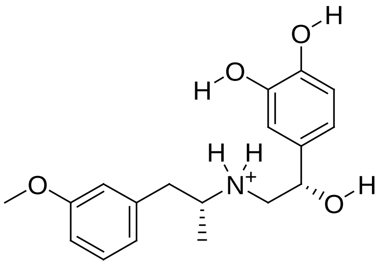

152 (NCI 21194)

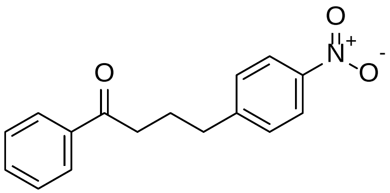

153 (NCI 3021)

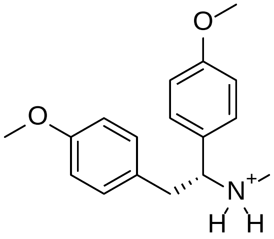

154 (NCI 34688)

"NMR" Charts &Mass Spectrum

"NMR" Charts &Mass Spectrum For
Hit 88 (NCI 1576)

Figure S 3: ^{1}H-NMR Charts for hit 88 (NCI 1576)

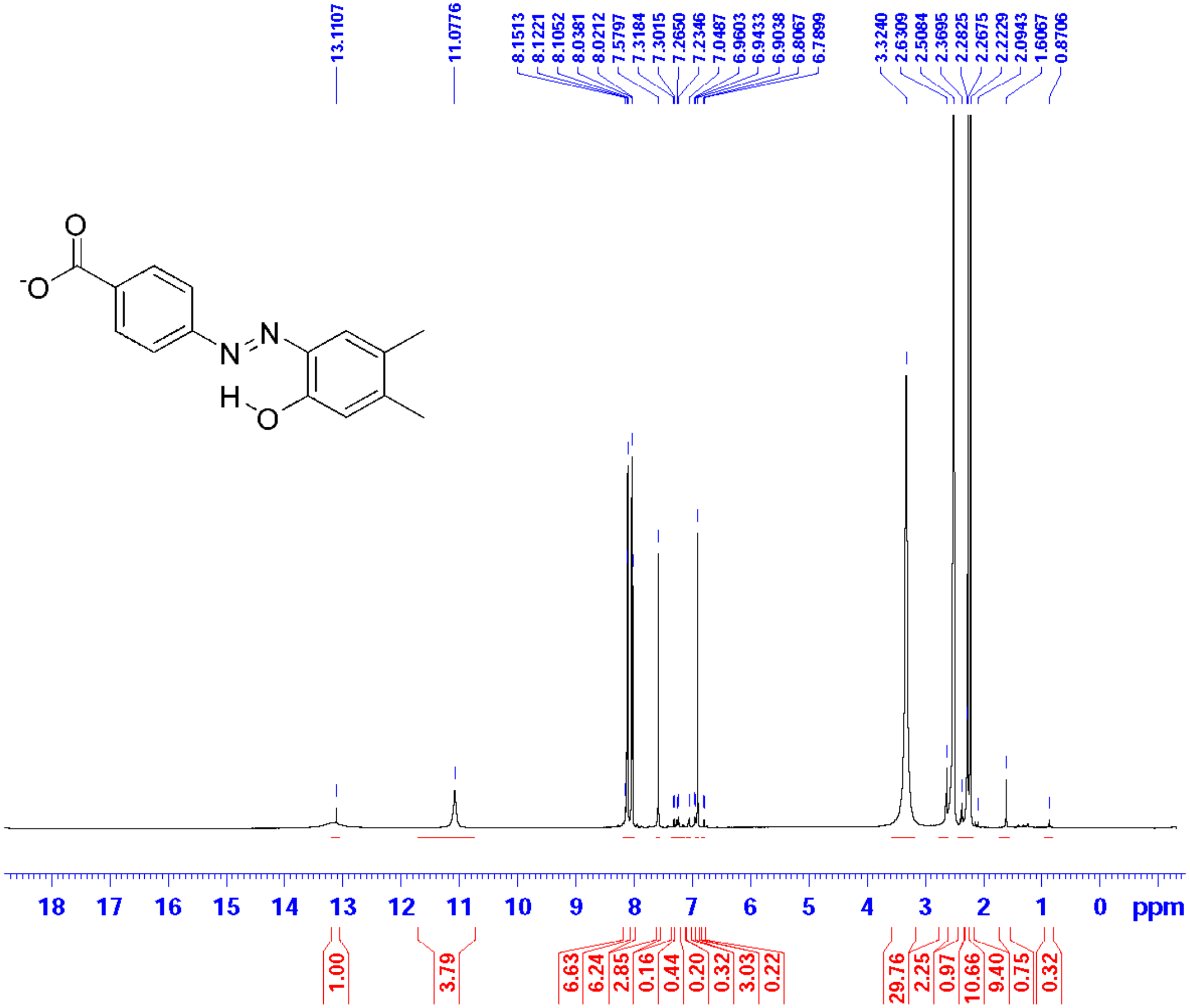

The University of Jordan
Faculty of Science
Department of Chemistry

Instrument Model:
Bruker 500 MHz–Avance III

Operator: Rola Hassouneh
nmr500@ju.edu.jo

```
Current Data Parameters
NAME          16oct15jalal
EXPNO                 1581
PROCNO                   1

F2 - Acquisition Parameters
Date_             20161026
Time                 12.49
INSTRUM              spect
PROBHD   5 mm PABBO BB/
PULPROG                 zg
TD                   65536
SOLVENT               DMSO
NS                      16
DS                       0
SWH            10135.135 Hz
FIDRES          0.154650 Hz
AQ             3.2331092 sec
RG                   64.29
DW                  49.333 usec
DE                    6.50 usec
TE                   300.5 K
D1            2.00000000 sec
TD0                      1

======== CHANNEL f1 ========
SFO1         500.1344108 MHz
NUC1                    1H
P1                   10.60 usec
PLW1           17.39999962 W

F2 - Processing parameters
SI                  131072
SF           500.1300000 MHz
WDW                     EM
SSB      0
LB                    1.00 Hz
GB       0
PC                    2.00
```

Figure S 4: ^{1}H-NMR Charts for hit 88 (NCI 1576)

1
H1 NMR

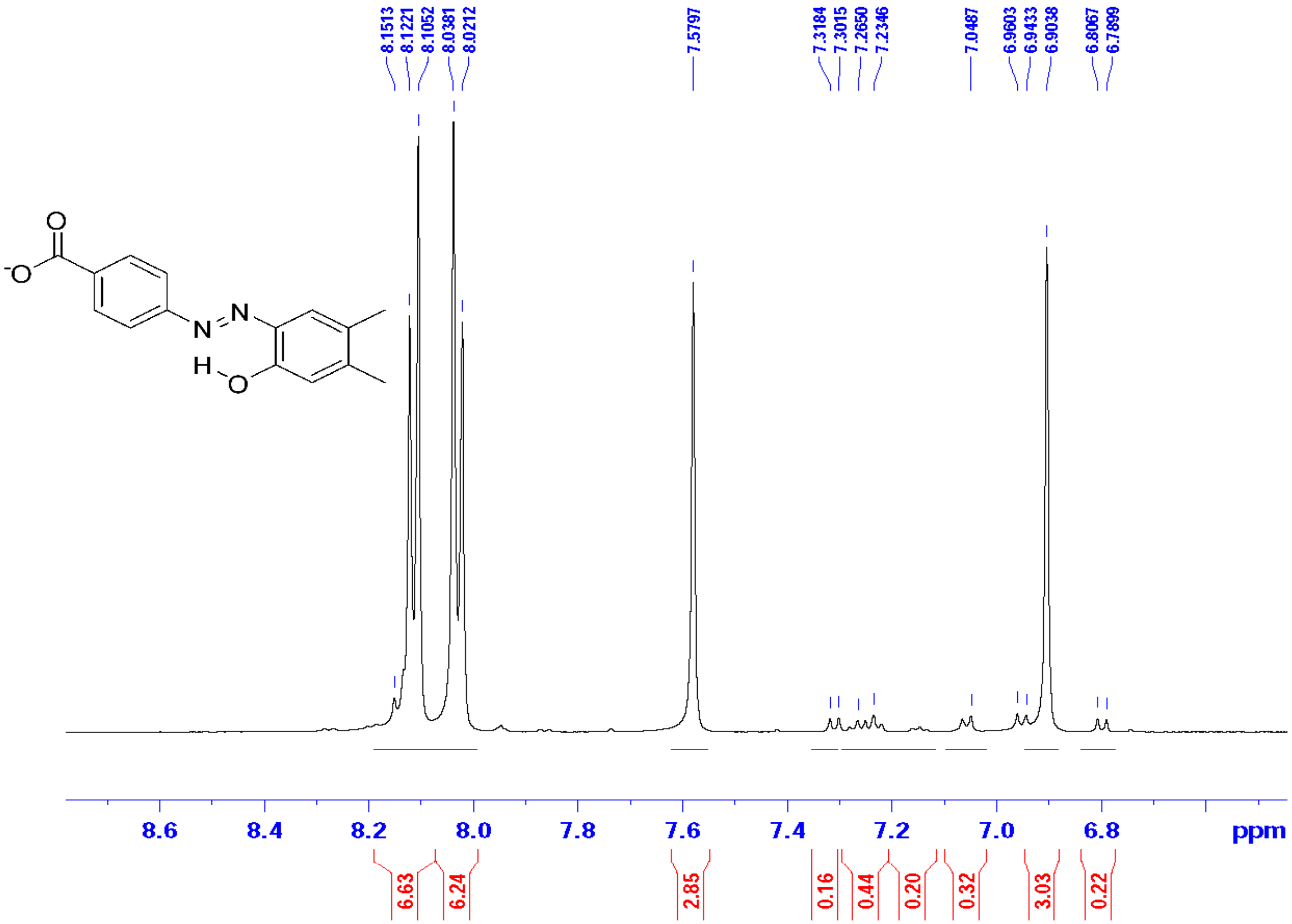

The University of Jordan
Faculty of Science
Department of Chemistry

Instrument Model:
Bruker 500 MHz–Avance III

Operator: Rola Hassouneh
nmr500@ju.edu.jo

```
Current Data Parameters
NAME          16oct15jalal
EXPNO                 1581
PROCNO                   1

F2 - Acquisition Parameters
Date_             20161026
Time                 12.49
INSTRUM              spect
PROBHD     5 mm PABBO BB/
PULPROG                 zg
TD                   65536
SOLVENT               DMSO
NS                      16
DS                       0
SWH              10135.135 Hz
FIDRES            0.154650 Hz
AQ               3.2331092 sec
RG                   64.29
DW                  49.333 usec
DE                    6.50 usec
TE                   300.5 K
D1              2.00000000 sec
TD0                      1

======== CHANNEL f1 ========
SFO1           500.1344108 MHz
NUC1                    1H
P1                   10.60 usec
PLW1           17.39999962 W

F2 - Processing parameters
SI                  131072
SF             500.1300000 MHz
WDW                     EM
SSB        0
LB                    1.00 Hz
GB         0
PC                    2.00
```

Figure S 5: ^{1}H-NMR Charts for Hit 88 (NCI 1576)

1
H1 NMR

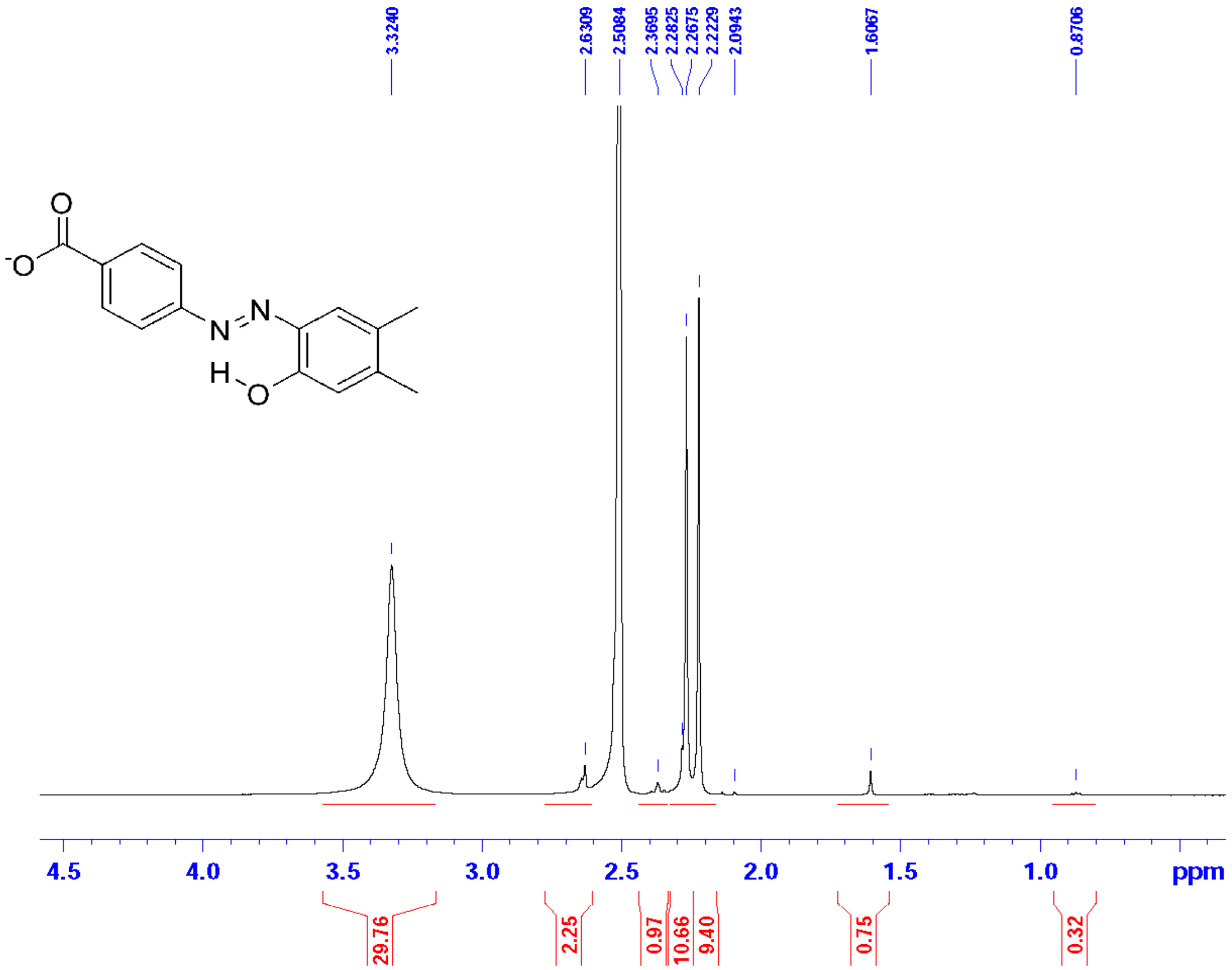

The University of Jordan
Faculty of Science
Department of Chemistry

Instrument Model:
Bruker 500 MHz–Avance III

Operator: Rola Hassouneh
nmr500@ju.edu.jo


```
Current Data Parameters
NAME          16oct15jalal
EXPNO                 1581
PROCNO                   1

F2 - Acquisition Parameters
Date_             20161026
Time                 12.49
INSTRUM              spect
PROBHD   5 mm PABBO BB/
PULPROG                 zg
TD                   65536
SOLVENT               DMSO
NS                      16
DS                       0
SWH             10135.135 Hz
FIDRES           0.154650 Hz
AQ              3.2331092 sec
RG                   64.29
DW                  49.333 usec
DE                    6.50 usec
TE                   300.5 K
D1              2.00000000 sec
TD0                      1

======== CHANNEL f1 ========
SFO1         500.1344108 MHz
NUC1                    1H
P1                   10.60 usec
PLW1           17.39999962 W

F2 - Processing parameters
SI                  131072
SF           500.1300000 MHz
WDW                     EM
SSB      0
LB                    1.00 Hz
GB       0
PC                    2.00
```

Figure S 6: ^{13}C-NMR Charts for Hit 88 (NCI 1576)

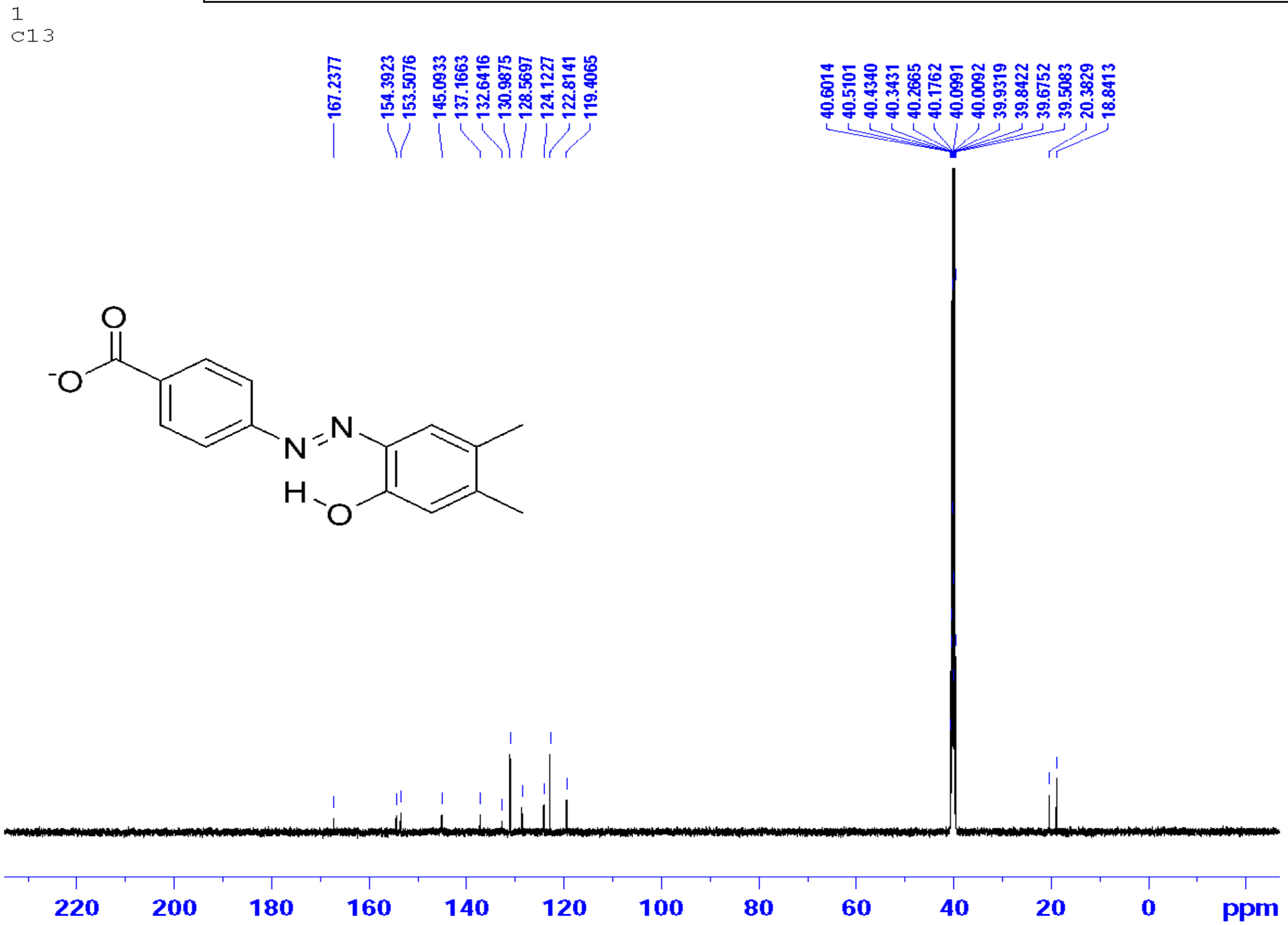

The University of Jordan
Faculty of Science
Department of Chemistry

Instrument Model:
Bruker 500 MHz–Avance III

Operator: Rola Hassouneh
nmr500@ju.edu.jo

```
Current Data Parameters
NAME          16oct15jalal
EXPNO                 1582
PROCNO                   1

F2 - Acquisition Parameters
Date_             20161026
Time                 12.57
INSTRUM              spect
PROBHD     5 mm PABBO BB/
PULPROG            zgpg30
TD                   65536
SOLVENT               DMSO
NS                     503
DS                       4
SWH             32894.738 Hz
FIDRES           0.501934 Hz
AQ              0.9961472 sec
RG                  202.06
DW                  15.200 usec
DE                    6.50 usec
TE                   300.5 K
D1              2.00000000 sec
D11             0.03000000 sec
TD0                      1

======== CHANNEL f1 ========
SFO1         125.7708561 MHz
NUC1                   13C
P1                   10.00 usec
PLW1           93.32499695 W

======== CHANNEL f2 ========
SFO2         500.1320005 MHz
NUC2                    1H
CPDPRG[2           waltz16
PCPD2                80.00 usec
PLW2           17.39999962 W
PLW12           0.31127000 W
PLW13           0.19921000 W

F2 - Processing parameters
SI                   32768
SF            125.7577890 MHz
WDW                     EM
SSB        0
LB                    1.00 Hz
GB         0
PC                    1.00
```

Figure S 7: ^{13}C-NMR Charts for Hit 88 (NCI 1576)

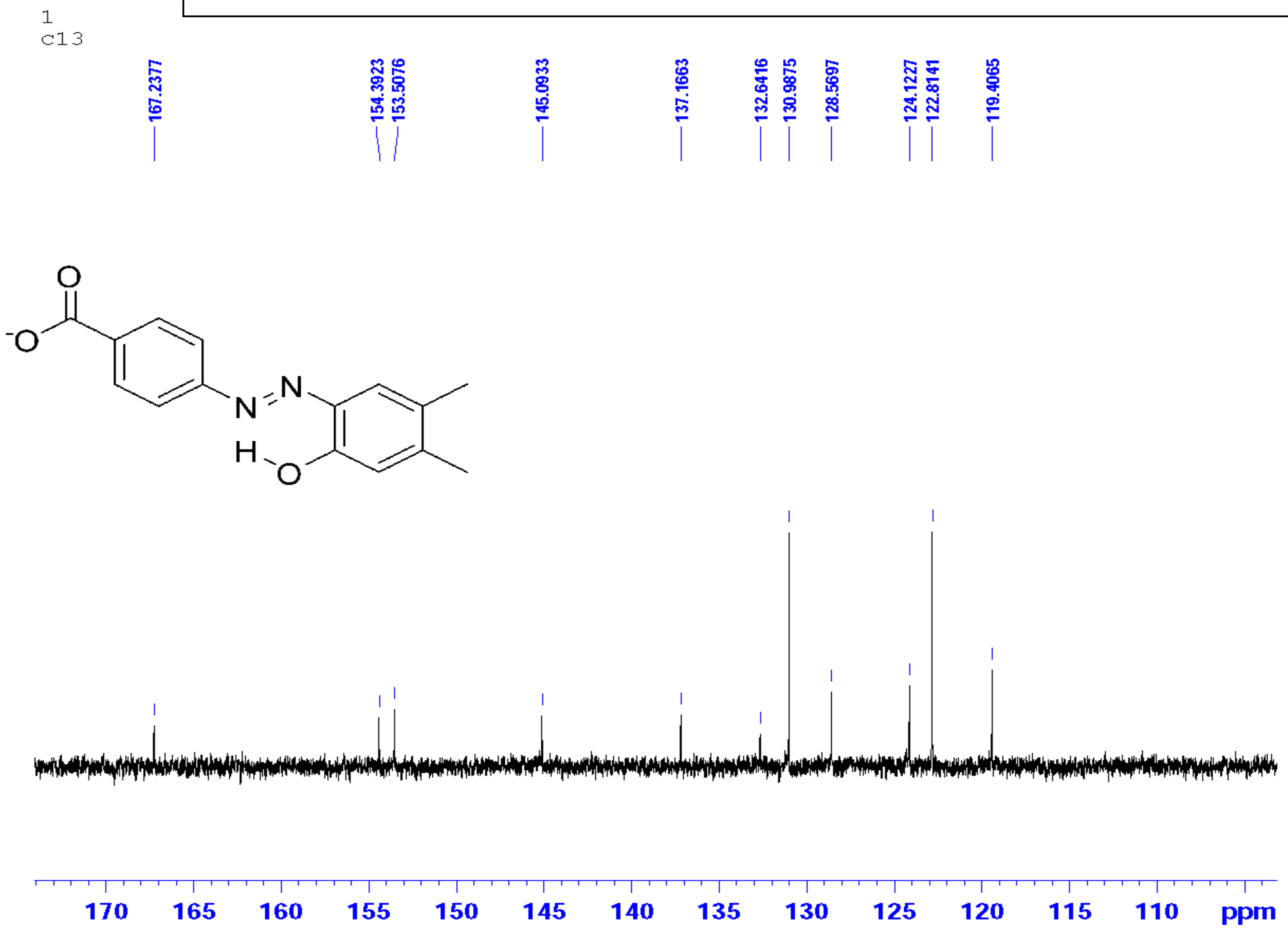

The University of Jordan
Faculty of Science
Department of Chemistry

Instrument Model:
Bruker 500 MHz–Avance III

Operator: Rola Hassouneh
nmr500@ju.edu.jo

```
Current Data Parameters
NAME           16oct15jalal
EXPNO                  1582
PROCNO                    1

F2 - Acquisition Parameters
Date_              20161026
Time                  12.57
INSTRUM               spect
PROBHD    5 mm PABBO BB/
PULPROG             zgpg30
TD                    65536
SOLVENT                DMSO
NS                      503
DS                        4
SWH              32894.738 Hz
FIDRES            0.501934 Hz
AQ               0.9961472 sec
RG                  202.06
DW                  15.200 usec
DE                    6.50 usec
TE                   300.5 K
D1              2.00000000 sec
D11             0.03000000 sec
TD0                       1

======== CHANNEL f1 ========
SFO1           125.7708561 MHz
NUC1                    13C
P1                    10.00 usec
PLW1            93.32499695 W

======== CHANNEL f2 ========
SFO2           500.1320005 MHz
NUC2                     1H
CPDPRG[2             waltz16
PCPD2                 80.00 usec
PLW2            17.39999962 W
PLW12            0.31127000 W
PLW13            0.19921000 W

F2 - Processing parameters
SI                    32768
SF             125.7577890 MHz
WDW                      EM
SSB      0
LB                     1.00 Hz
GB       0
PC                     1.00
```

The University of Jordan

Faculty of science

Department of Chemistry

Mass Spectrum Molecular Formula Report

Analysis Info

Analysis Name	F:\Data\2017MAY03\AREEJ_000051.d	Acquisition Date	5/16/2017 8:56:13 AM
Method	ESI-NEG-2017	Operator	Bruker_PC
Sample Name	1(1576)	Instrument	apex-IV
Comment	MEOH		

Acquisition Parameter

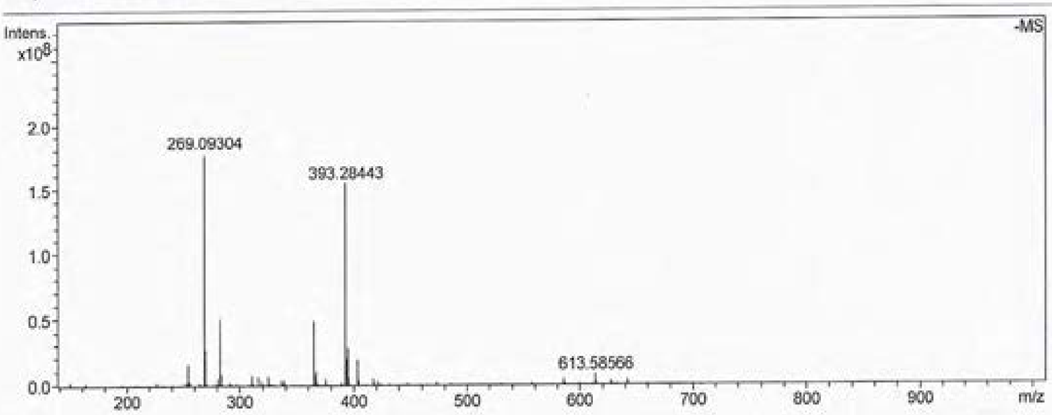

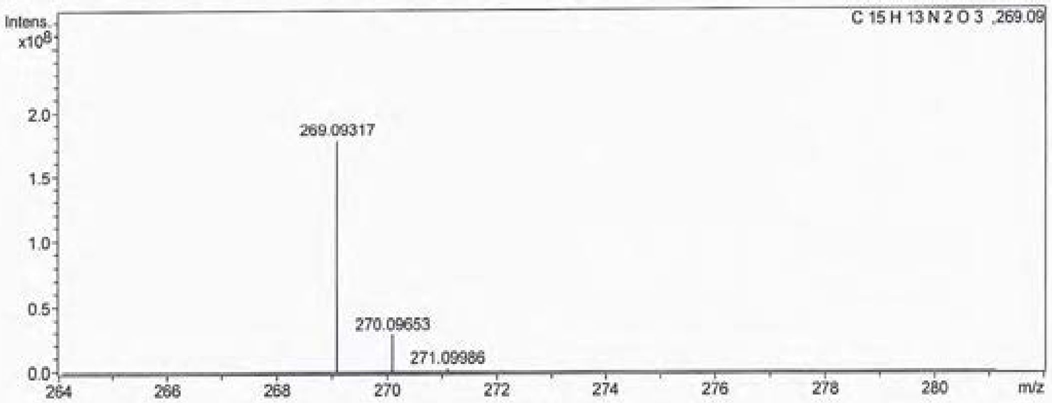

Sum Formula	Sigma	m/z	Err [ppm]	Mean Err [ppm]	Err [mDa]	rdb	N Rule	e⁻
C 15 H 13 N 2 O 3	0.015	269.09317	0.46	0.28	0.12	10.50	ok	even

Bruker Daltonics DataAnalysis 3.4 printed: 5/16/2017 9:01:08 AM Page 1 of 1

Figure S 8: Mass Spectrum for Hit 88 (NCI 1576)

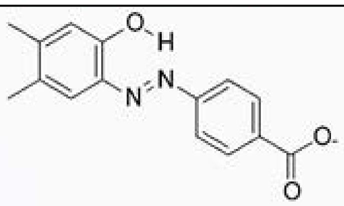

Mass Spectrum List Report

Analysis Info

Analysis Name	F:\Data\2017MAY03\AREEJ_000051.d	Acquisition Date	5/16/2017 8:56:13 AM
Method	ESI-NEG-2017	Operator	Bruker_PC
Sample Name	1(1576)	Instrument	apex-IV
Comment	MEOH		

Acquisition Parameter

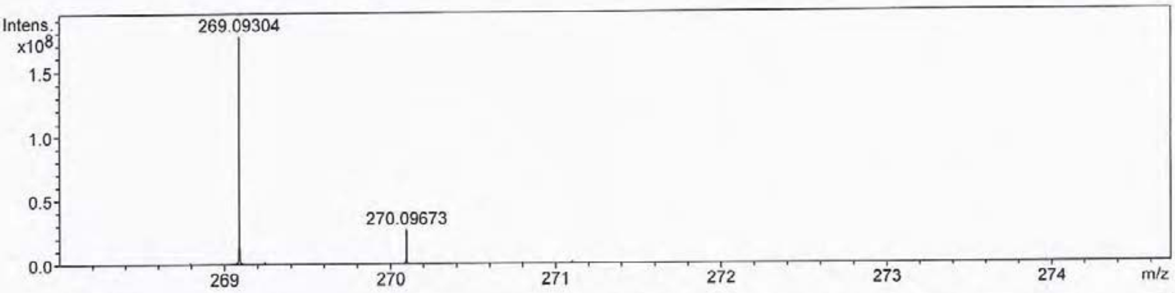

#	m/z	I	I %
1	255.23241	16350947	9.2
2	269.08727	6869651	3.9
3	269.08932	13541907	7.6
4	269.09304	178274451	100.0
5	269.09679	12893459	7.2
6	269.09882	5517075	3.1
7	270.09673	27050912	15.2
8	281.24958	5382943	3.0
9	283.26506	50370482	28.3
10	284.26871	8608940	4.8
11	311.17166	7326088	4.1
12	316.60782	7053225	4.0
13	325.18831	6509615	3.7
14	365.24650	6894570	3.9
15	365.25328	49110762	27.5
16	366.25728	7952919	4.5
17	367.25103	10625123	6.0
18	393.27150	5540606	3.1
19	393.28443	154574078	86.7
20	393.29229	12825982	7.2
21	393.29667	5952574	3.3
22	394.28443	17948995	10.1
23	394.29161	19576451	11.0
24	395.27783	25243272	14.2
25	395.28491	28261768	15.9
26	396.28209	6217933	3.5
27	396.28947	5956237	3.3
28	403.31044	13720481	7.7
29	403.31791	19158689	10.7
30	613.58566	8087291	4.5

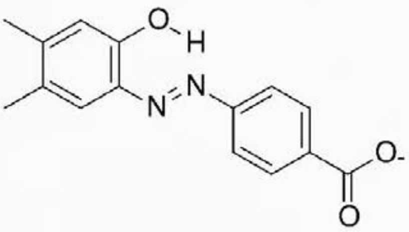

Figure S 9: Mass Spectrum for Hit 88 (NCI 1576)

Mass Spectrum List Report

Analysis Info

Analysis Name F:\Data\2017MAY03\AREEJ_000051.d
Method ESI-NEG-2017
Sample Name 1(1576)
Comment MEOH

Acquisition Date 5/16/2017 8:56:13 AM
Operator Bruker_PC
Instrument apex-IV

Acquisition Parameter

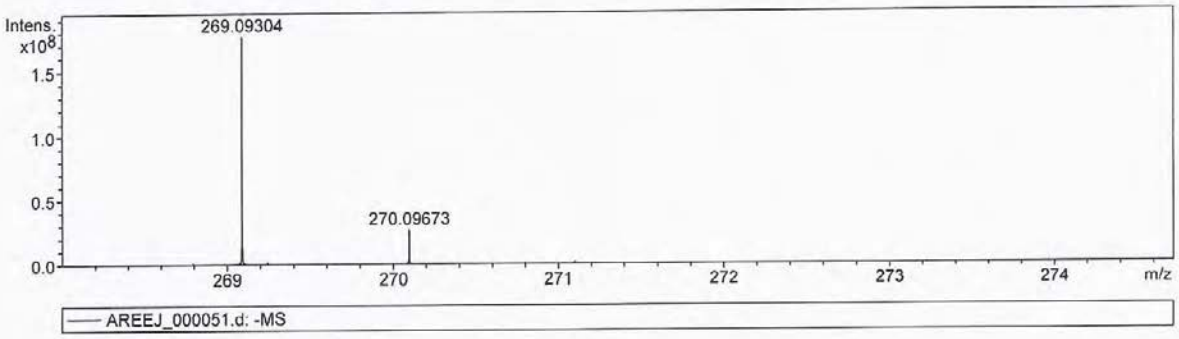

#	m/z	I	I %
1	255.23241	16350947	9.2
2	269.08727	6869651	3.9
3	269.08932	13541907	7.6
4	269.09304	178274451	100.0
5	269.09679	12893459	7.2
6	269.09882	5517075	3.1
7	270.09673	27050912	15.2
8	281.24958	5382943	3.0
9	283.26506	50370482	28.3
10	284.26871	8608940	4.8
11	311.17166	7326088	4.1
12	316.60782	7053225	4.0
13	325.18831	6509615	3.7
14	365.24650	6894570	3.9
15	365.25328	49110762	27.5
16	366.25728	7952919	4.5
17	367.25103	10625123	6.0
18	393.27150	5540606	3.1
19	393.28443	154574078	86.7
20	393.29229	12825982	7.2
21	393.29667	5952574	3.3
22	394.28443	17948995	10.1
23	394.29161	19576451	11.0
24	395.27783	25243272	14.2
25	395.28491	28261768	15.9
26	396.28209	6217933	3.5
27	396.28947	5956237	3.3
28	403.31044	13720481	7.7
29	403.31791	19158689	10.7
30	613.58566	8087291	4.5

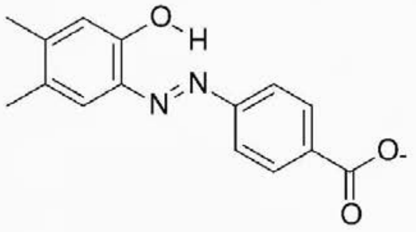

Figure S 10: Mass Spectrum for Hit 88 (NCI 1576)

"NMR" Charts &Mass Spectrum For Hit 130 (NCI 12849)

Figure S 11: ^{1}H-NMR Charts for hit 130 (NCI 12849)

16
H1 NMR

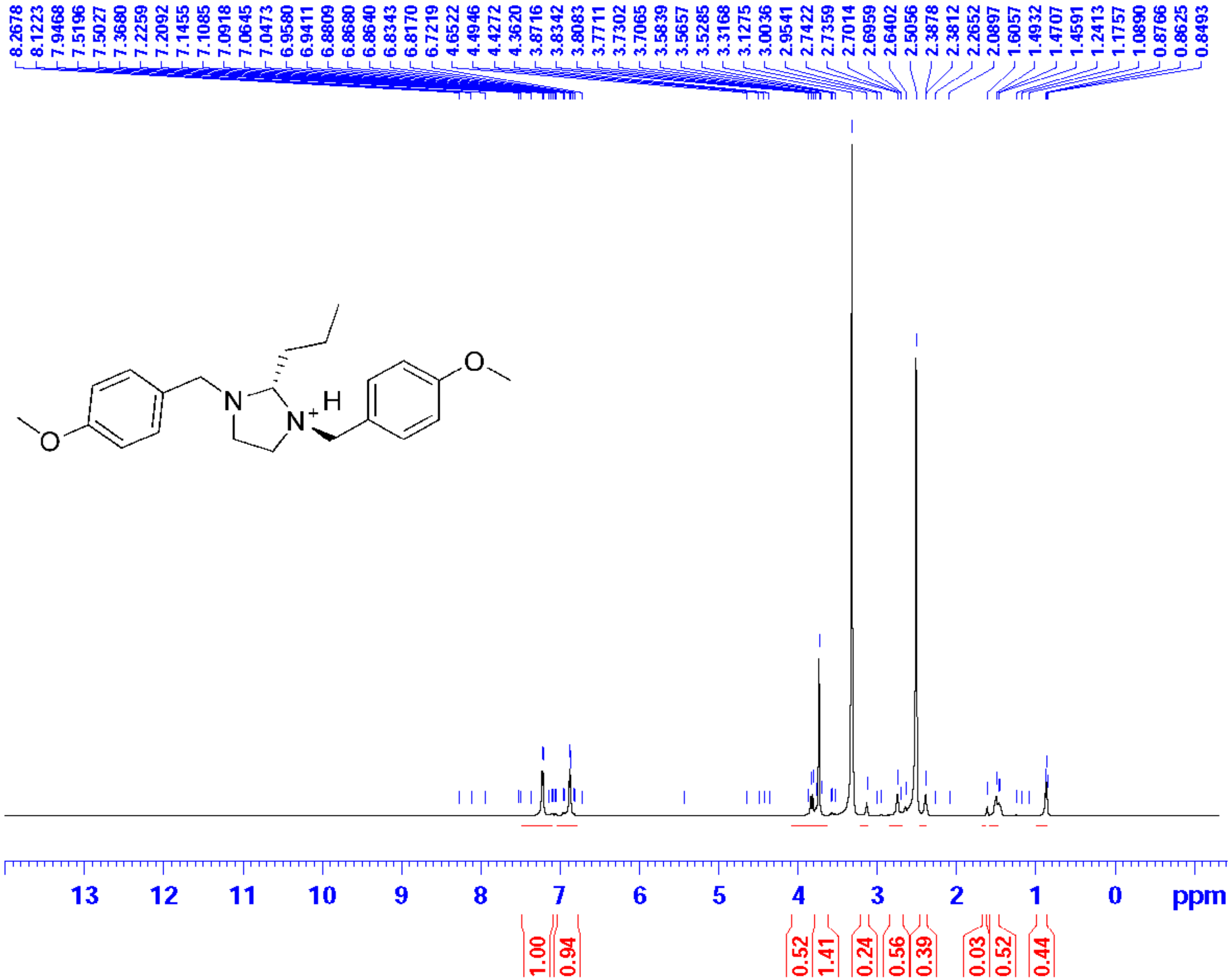

The University of Jordan
Faculty of Science
Department of Chemistry

Instrument Model:
Bruker 500 MHz–Avance III

Operator: Rola Hassouneh
nmr500@ju.edu.jo

Current Data Parameters
NAME 16oct15jalal
EXPNO 2031
PROCNO 1

F2 - Acquisition Parameters
Date_ 20161027
Time 7.59
INSTRUM spect
PROBHD 5 mm PABBO BB/
PULPROG zg
TD 65536
SOLVENT DMSO
NS 16
DS 0
SWH 10135.135 Hz
FIDRES 0.154650 Hz
AQ 3.2331092 sec
RG 64.29
DW 49.333 usec
DE 6.50 usec
TE 300.4 K
D1 2.00000000 sec
TD0 1

======== CHANNEL f1 ========
SFO1 500.1344108 MHz
NUC1 1H
P1 10.60 usec
PLW1 17.39999962 W

F2 - Processing parameters
SI 131072
SF 500.1300000 MHz
WDW EM
SSB 0
LB 1.00 Hz
GB 0
PC 2.00

16
H1 NMR

Figure S 12: ^{1}H-NMR Charts for hit 130 (NCI 12849)

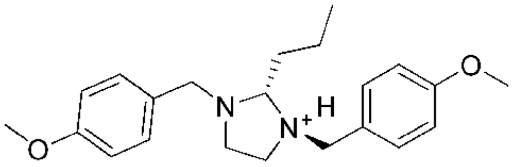

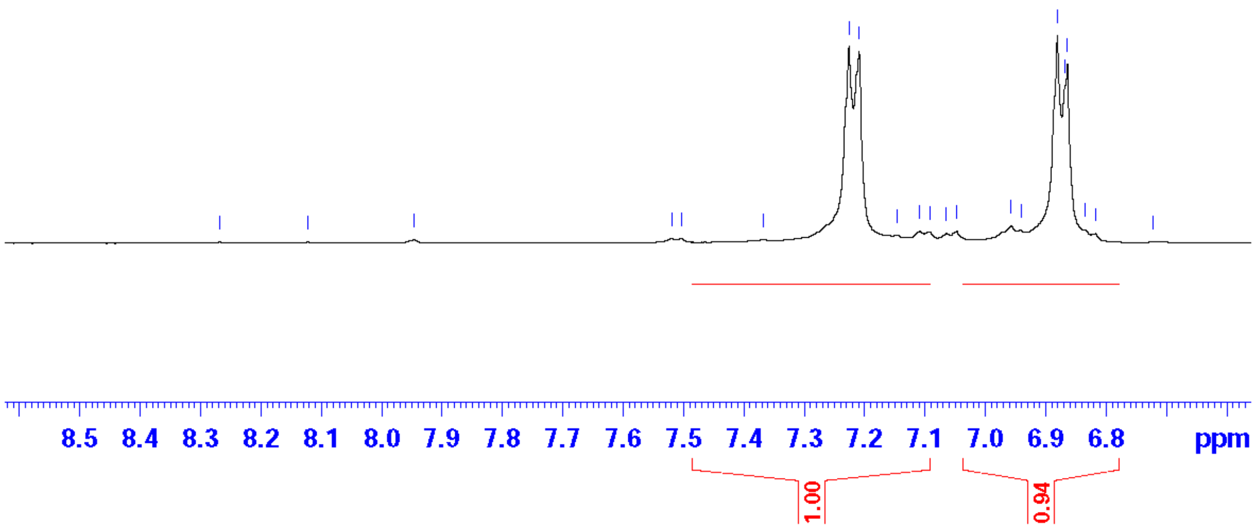

The University of Jordan
Faculty of Science
Department of Chemistry

Instrument Model:
Bruker 500 MHz–Avance III

Operator: Rola Hassouneh
nmr500@ju.edu.jo


```
Current Data Parameters
NAME          16oct15jalal
EXPNO                 2031
PROCNO                   1

F2 - Acquisition Parameters
Date_             20161027
Time                  7.59
INSTRUM              spect
PROBHD   5 mm PABBO BB/
PULPROG                 zg
TD                   65536
SOLVENT               DMSO
NS                      16
DS                       0
SWH            10135.135 Hz
FIDRES          0.154650 Hz
AQ             3.2331092 sec
RG                   64.29
DW                  49.333 usec
DE                    6.50 usec
TE                   300.4 K
D1            2.00000000 sec
TD0                      1

======== CHANNEL f1 ========
SFO1        500.1344108 MHz
NUC1                    1H
P1                   10.60 usec
PLW1           17.39999962 W

F2 - Processing parameters
SI                  131072
SF          500.1300000 MHz
WDW                     EM
SSB        0
LB                    1.00 Hz
GB         0
PC                    2.00
```

16
H1 NMR

Figure S 13: ^{1}H-NMR Charts for hit 130 (NCI 12849)

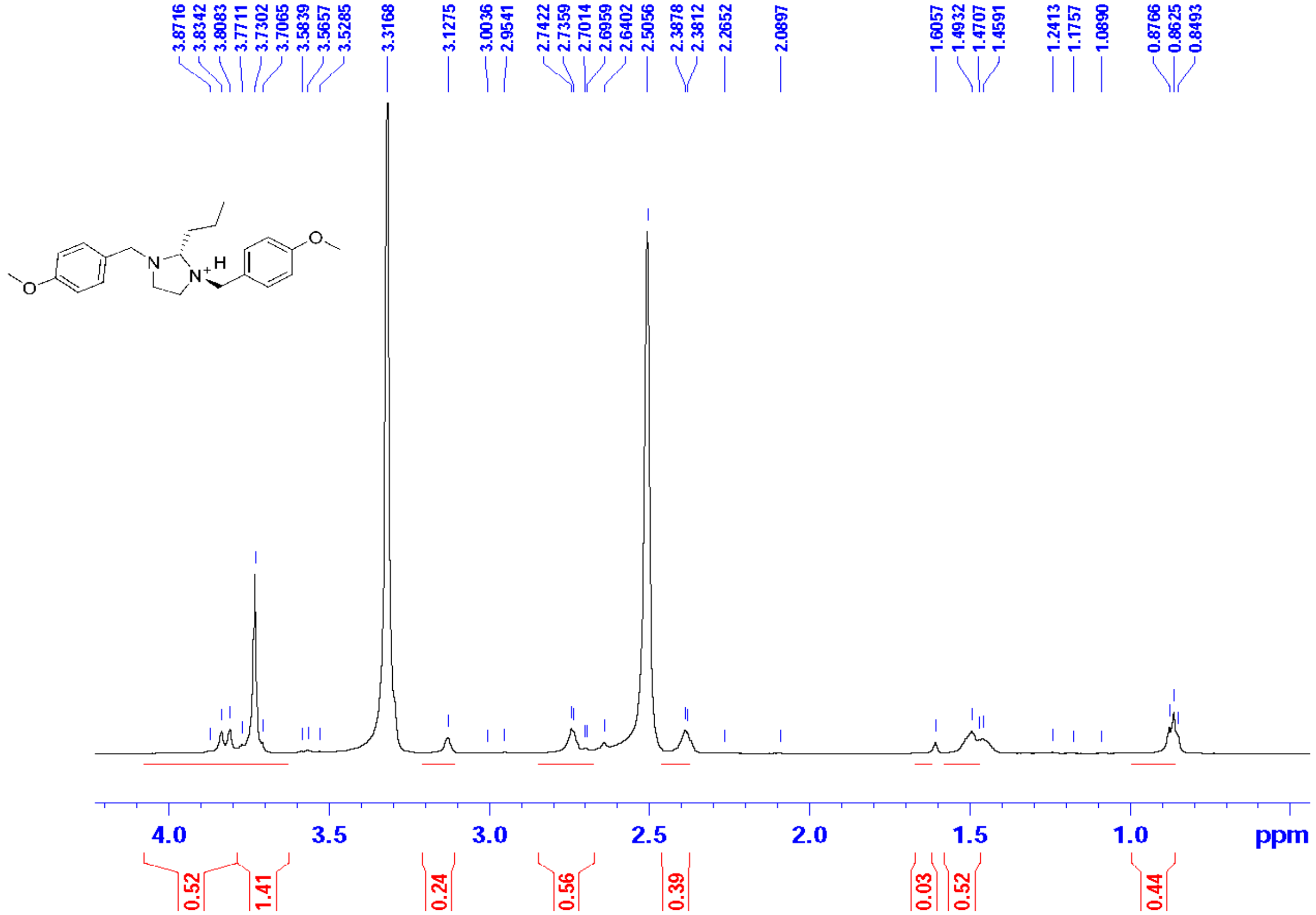

The University of Jordan
Faculty of Science
Department of Chemistry

Instrument Model:
Bruker 500 MHz–Avance III

Operator: Rola Hassouneh
nmr500@ju.edu.jo


```
Current Data Parameters
NAME          16oct15jalal
EXPNO                 2031
PROCNO                   1

F2 - Acquisition Parameters
Date_             20161027
Time                  7.59
INSTRUM              spect
PROBHD    5 mm PABBO BB/
PULPROG                 zg
TD                   65536
SOLVENT               DMSO
NS                      16
DS                       0
SWH             10135.135 Hz
FIDRES           0.154650 Hz
AQ              3.2331092 sec
RG                   64.29
DW                  49.333 usec
DE                    6.50 usec
TE                   300.4 K
D1              2.00000000 sec
TD0                      1

======== CHANNEL f1 ========
SFO1         500.1344108 MHz
NUC1                  1H
P1                 10.60 usec
PLW1         17.39999962 W

F2 - Processing parameters
SI                131072
SF           500.1300000 MHz
WDW                   EM
SSB      0
LB                  1.00 Hz
GB       0
PC                  2.00
```

16
c13

Figure S 14: ^{13}C-NMR Charts for hit 130 (NCI 12849)

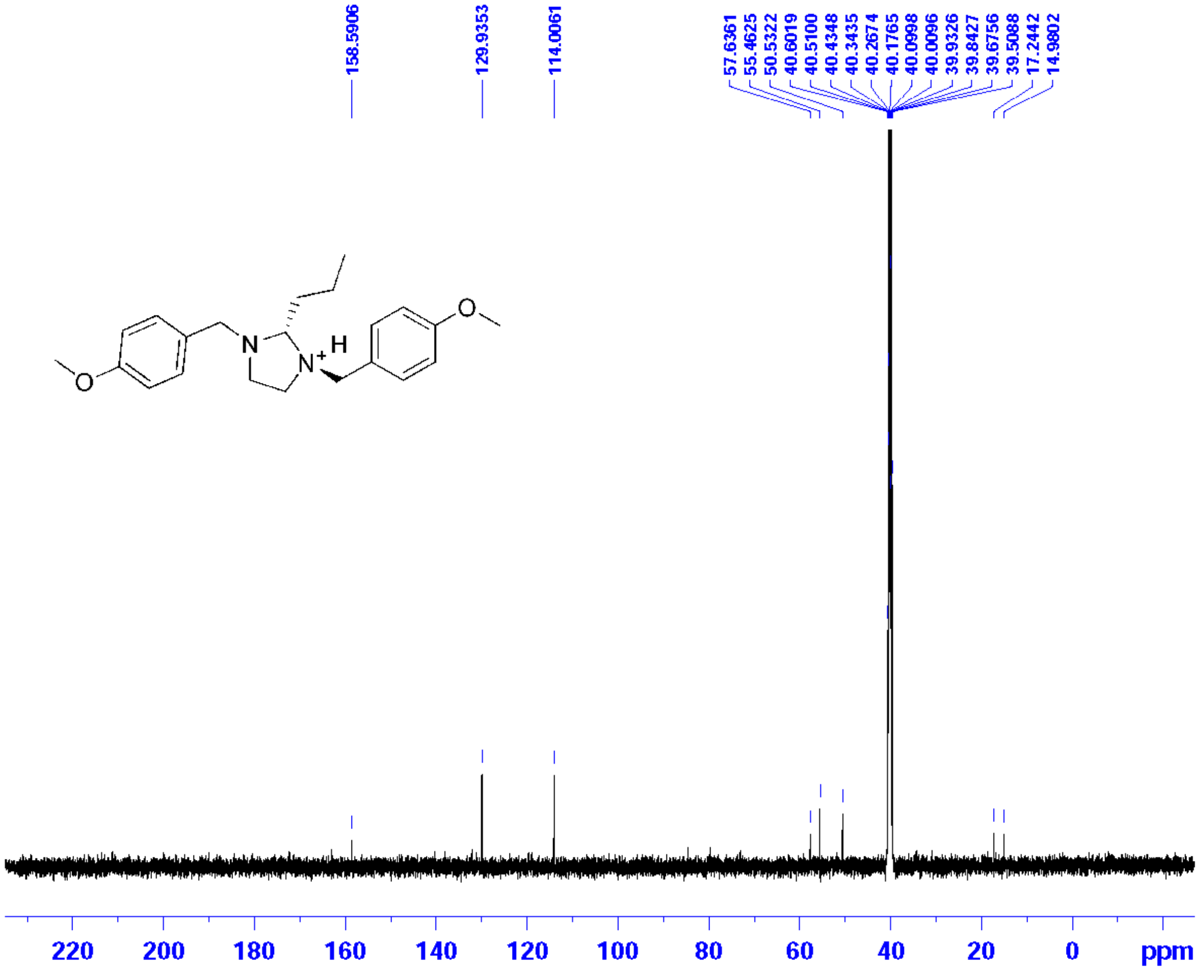

The University of Jordan
Faculty of Science
Department of Chemistry

Instrument Model:
Bruker 500 MHz–Avance III

Operator: Rola Hassouneh
nmr500@ju.edu.jo


```
Current Data Parameters
NAME        16oct15jalal
EXPNO              2032
PROCNO                1

F2 - Acquisition Parameters
Date_          20161027
Time               8.09
INSTRUM           spect
PROBHD   5 mm PABBO BB/
PULPROG         zgpg30
TD                65536
SOLVENT            DMSO
NS                  521
DS                    4
SWH           32894.738 Hz
FIDRES         0.501934 Hz
AQ            0.9961472 sec
RG               202.06
DW               15.200 usec
DE                 6.50 usec
TE                300.5 K
D1           2.00000000 sec
D11          0.03000000 sec
TD0                   1

======== CHANNEL f1 ========
SFO1        125.7708561 MHz
NUC1                13C
P1                10.00 usec
PLW1        93.32499695 W

======== CHANNEL f2 ========
SFO2        500.1320005 MHz
NUC2                 1H
CPDPRG[2        waltz16
PCPD2             80.00 usec
PLW2        17.39999962 W
PLW12        0.31127000 W
PLW13        0.19921000 W

F2 - Processing parameters
SI                32768
SF          125.7577890 MHz
WDW                  EM
SSB      0
LB                 1.00 Hz
GB       0
PC                 1.50
```

16
c13

Figure S 15: ^{13}C-NMR Charts for hit 130 (NCI 12849)

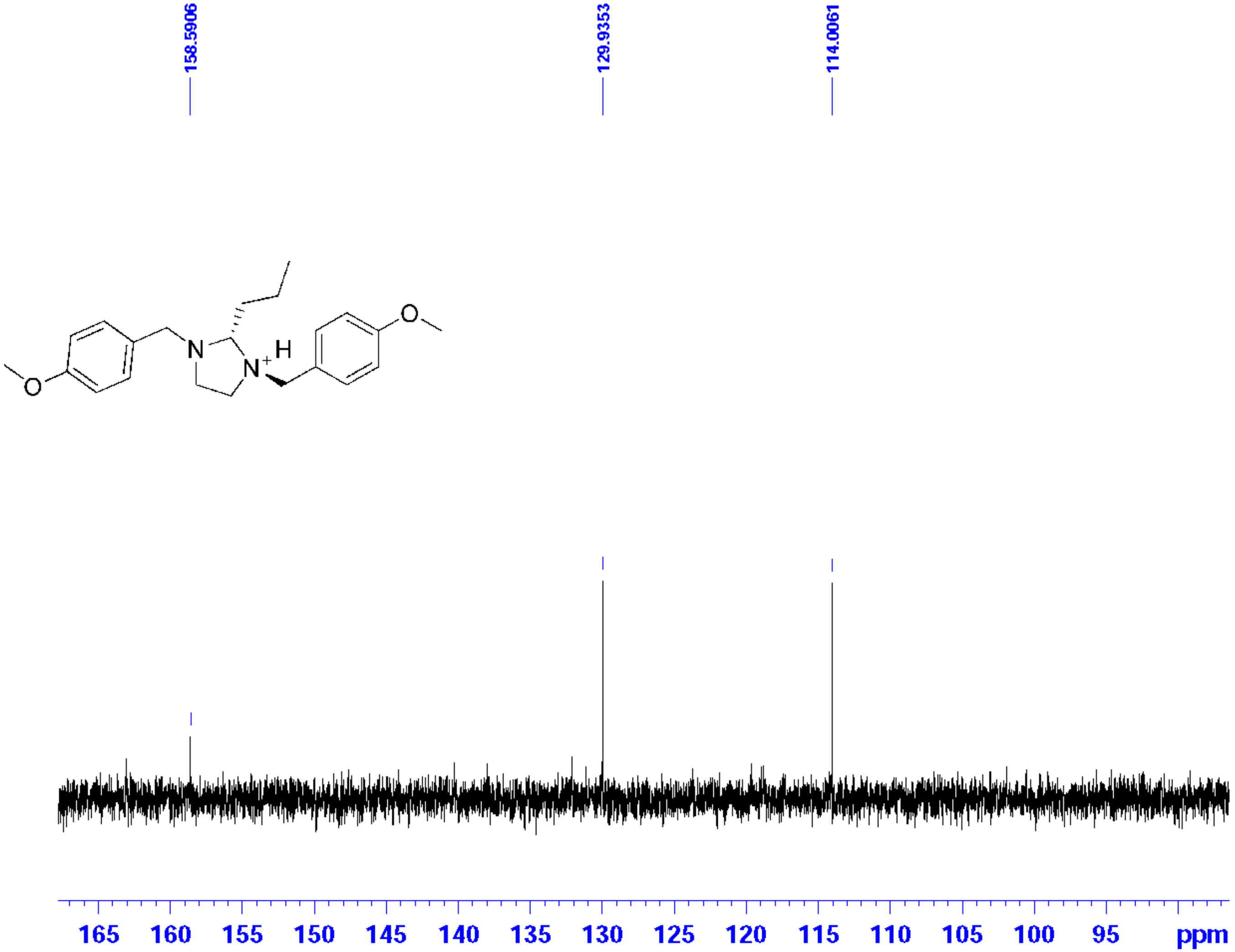

The University of Jordan
Faculty of Science
Department of Chemistry

Instrument Model:
Bruker 500 MHz–Avance III

Operator: Rola Hassouneh
nmr500@ju.edu.jo


```
Current Data Parameters
NAME         16oct15jalal
EXPNO                2032
PROCNO                  1

F2 - Acquisition Parameters
Date_            20161027
Time                 8.09
INSTRUM             spect
PROBHD    5 mm PABBO BB/
PULPROG            zgpg30
TD                  65536
SOLVENT              DMSO
NS                    521
DS                      4
SWH             32894.738 Hz
FIDRES           0.501934 Hz
AQ              0.9961472 sec
RG                 202.06
DW                 15.200 usec
DE                   6.50 usec
TE                  300.5 K
D1             2.00000000 sec
D11            0.03000000 sec
TD0                     1

======== CHANNEL f1 ========
SFO1          125.7708561 MHz
NUC1                  13C
P1                  10.00 usec
PLW1          93.32499695 W

======== CHANNEL f2 ========
SFO2          500.1320005 MHz
NUC2                   1H
CPDPRG[2          waltz16
PCPD2               80.00 usec
PLW2          17.39999962 W
PLW12          0.31127000 W
PLW13          0.19921000 W

F2 - Processing parameters
SI                  32768
SF            125.7577890 MHz
WDW                    EM
SSB      0
LB                   1.00 Hz
GB       0
PC                   1.50
```

Figure S 16: ^{13}C-NMR Charts for hit 130 (NCI 12849)

16
c13

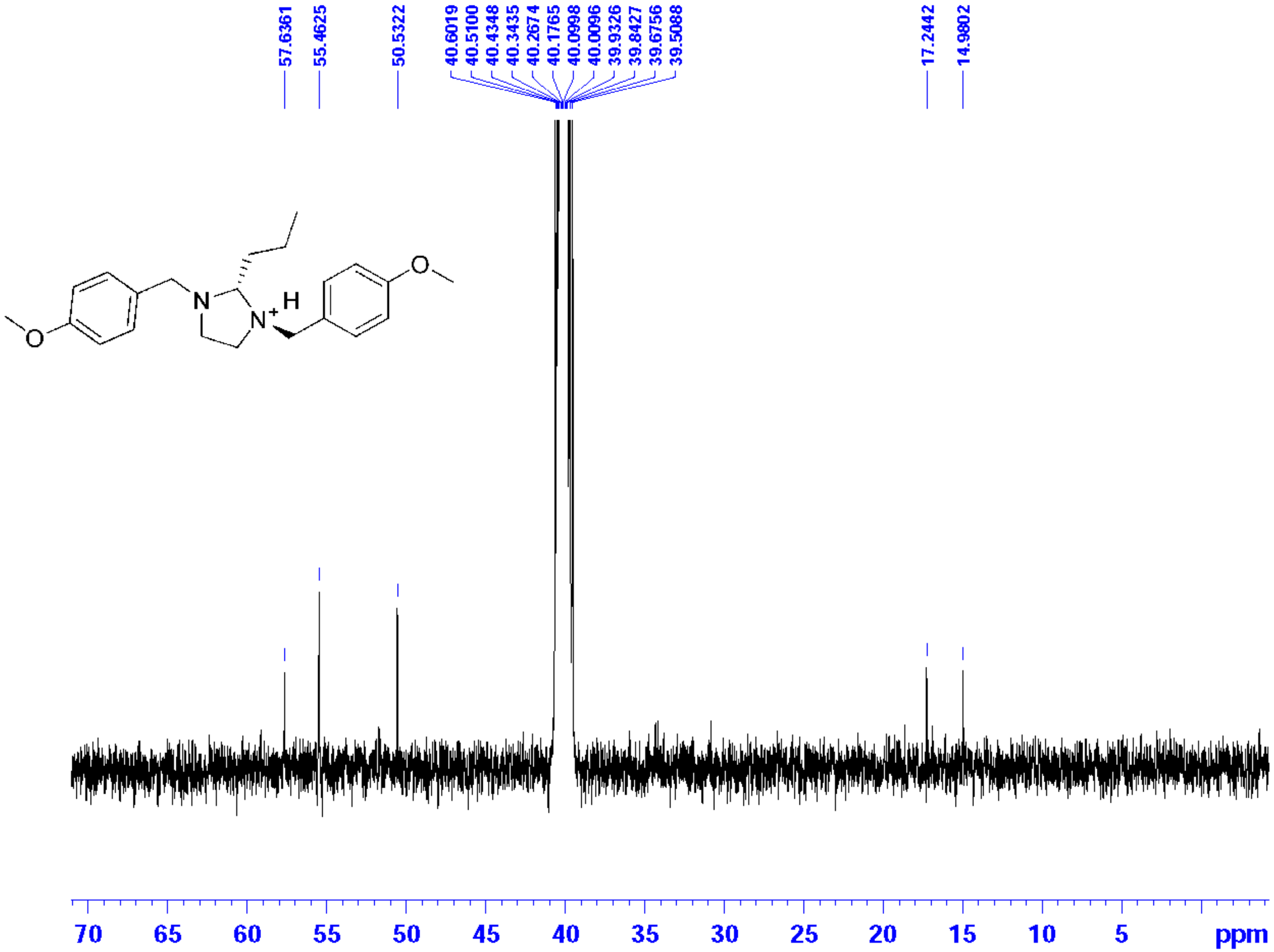

The University of Jordan
Faculty of Science
Department of Chemistry

Instrument Model:
Bruker 500 MHz–Avance III

Operator: Rola Hassouneh
nmr500@ju.edu.jo


```
Current Data Parameters
NAME          16oct15jalal
EXPNO                 2032
PROCNO                   1

F2 - Acquisition Parameters
Date_             20161027
Time                  8.09
INSTRUM              spect
PROBHD     5 mm PABBO BB/
PULPROG            zgpg30
TD                   65536
SOLVENT               DMSO
NS                     521
DS                       4
SWH             32894.738 Hz
FIDRES           0.501934 Hz
AQ              0.9961472 sec
RG                  202.06
DW                  15.200 usec
DE                    6.50 usec
TE                   300.5 K
D1             2.00000000 sec
D11            0.03000000 sec
TD0                      1

======== CHANNEL f1 ========
SFO1          125.7708561 MHz
NUC1                   13C
P1                   10.00 usec
PLW1           93.32499695 W

======== CHANNEL f2 ========
SFO2          500.1320005 MHz
NUC2                    1H
CPDPRG[2           waltz16
PCPD2                80.00 usec
PLW2           17.39999962 W
PLW12           0.31127000 W
PLW13           0.19921000 W

F2 - Processing parameters
SI                   32768
SF            125.7577890 MHz
WDW                     EM
SSB        0
LB                    1.00 Hz
GB         0
PC                    1.50
```

The University of Jordan
Faculty of science
Department of Chemistry

Mass Spectrum Molecular Formula Report

Analysis Info

Analysis Name	F:\Data\2017MAY03\AREEJ_000052.d	Acquisition Date	5/16/2017 9:03:40 AM
Method	ESI-POS-2017	Operator	Bruker_PC
Sample Name	16(12849)	Instrument	apex-IV
Comment	MEOH		

Acquisition Parameter

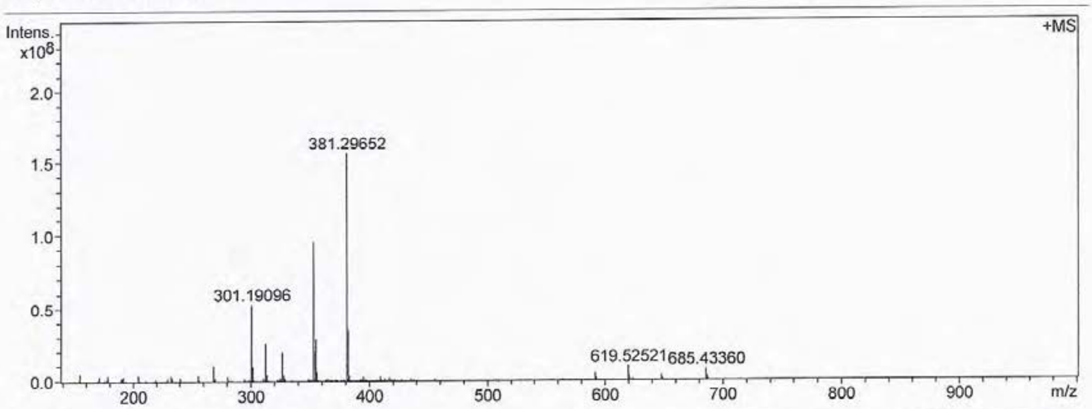

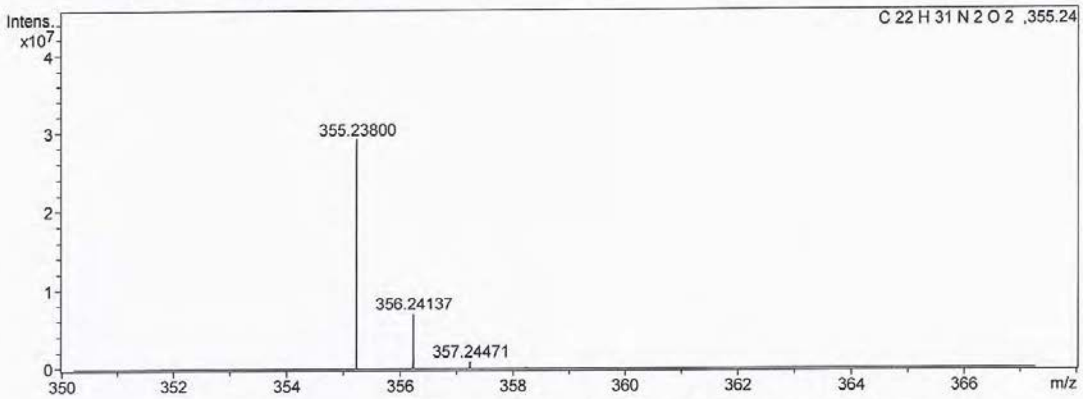

Sum Formula	Sigma	m/z	Err [ppm]	Mean Err [ppm]	Err [mDa]	rdb	N Rule	e⁻
C 22 H 31 N 2 O 2	0.017	355.23800	1.15	0.97	0.41	8.50	ok	even

Figure S 17: Mass Spectrum for hit 130 (NCI 12849)

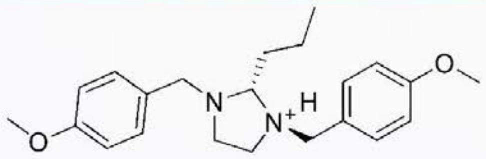

Mass Spectrum List Report

Analysis Info

Analysis Name	F:\Data\2017MAY03\AREEJ_000052.d
Method	ESI-POS-2017
Sample Name	16(12849)
Comment	MEOH
Acquisition Date	5/16/2017 9:03:40 AM
Operator	Bruker_PC
Instrument	apex-IV

Acquisition Parameter

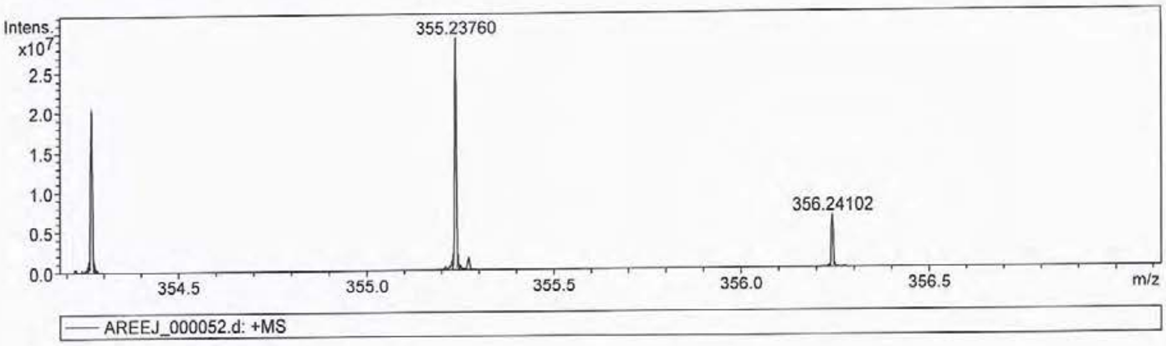

#	m/z	I	I %
1	155.31123	5656210	3.6
2	179.11814	3951963	2.5
3	205.13381	4377639	2.8
4	256.13362	3995775	2.6
5	269.16512	10425320	6.7
6	301.14136	3768547	2.4
7	301.19096	53069171	34.0
8	302.19447	10001374	6.4
9	313.19103	25804915	16.5
10	314.19456	4719054	3.0
11	327.20656	19996683	12.8
12	328.21000	4095797	2.6
13	353.25543	3851968	2.5
14	353.25916	6983936	4.5
15	353.26566	96340992	61.6
16	353.27218	6727936	4.3
17	354.26923	20478344	13.1
18	355.23760	29100815	18.6
19	356.24102	6540407	4.2
20	381.28456	6382676	4.1
21	381.28901	11449492	7.3
22	381.29652	156310164	100.0
23	381.30414	10663060	6.8
24	381.30812	3875028	2.5
25	382.30019	35555223	22.7
26	383.30325	3833897	2.5
27	591.49351	5448197	3.5
28	619.52521	9776843	6.3
29	647.55685	3885048	2.5
30	685.43360	7750803	5.0

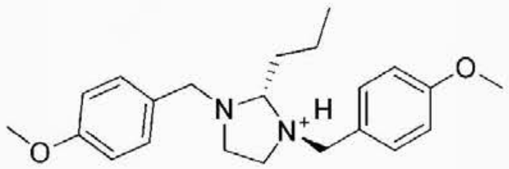

Figure S 18: Mass Spectrum for hit 130 (NCI 12849)

"NMR" Charts &Mass Spectrum For Hit 85 (NCI 14040)

Figure S 19: ^{1}H-NMR Charts for 85 (NCI 14040)

17
H1 NMR

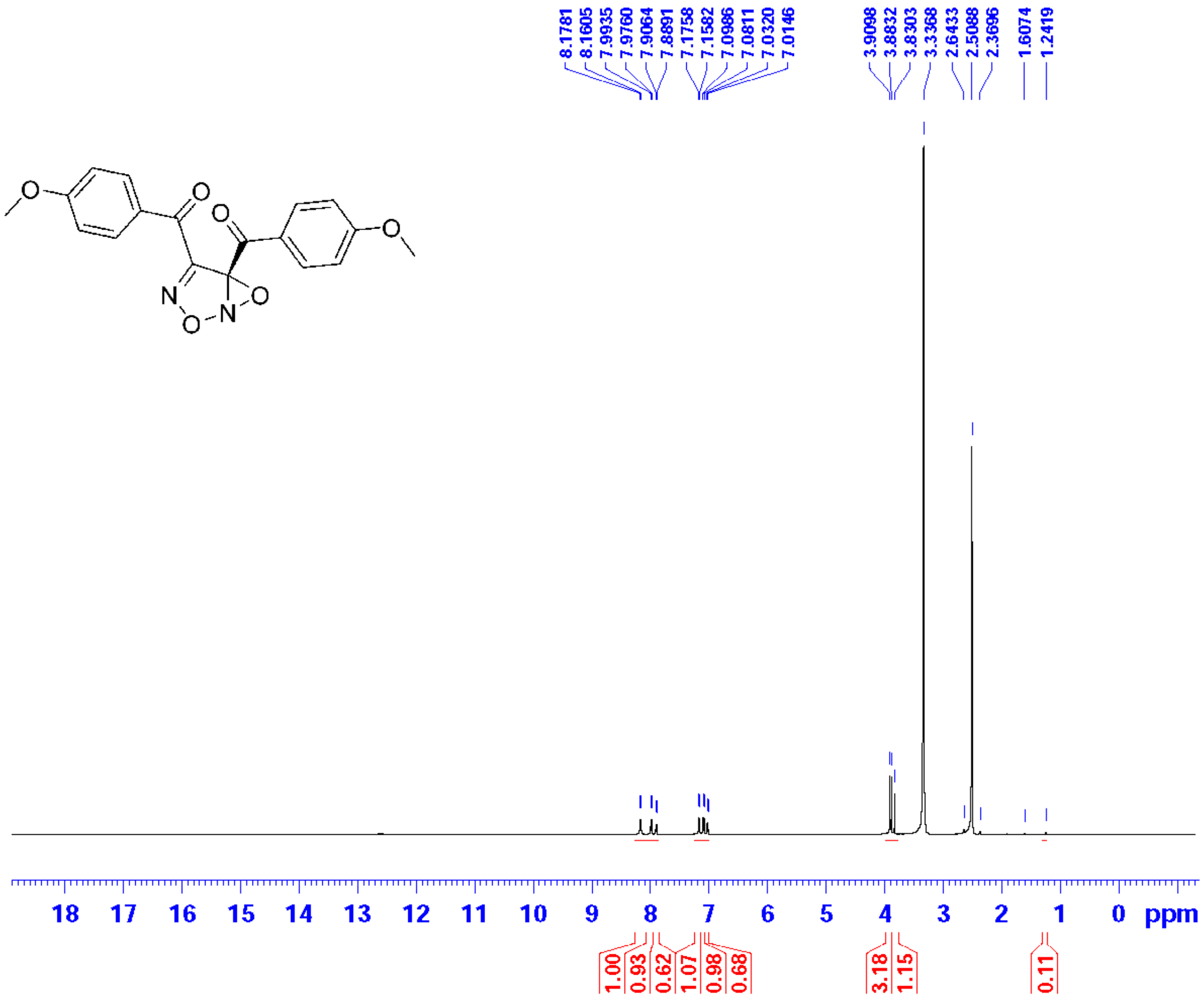

The University of Jordan
Faculty of Science
Department of Chemistry

Instrument Model:
Bruker 500 MHz–Avance III

Operator: Rola Hassouneh
nmr500@ju.edu.jo

```
Current Data Parameters
NAME          16nov15jalal
EXPNO                 1411
PROCNO                   1

F2 - Acquisition Parameters
Date_             20161122
Time                 10.25
INSTRUM              spect
PROBHD     5 mm PABBO BB/
PULPROG                 zg
TD                   65536
SOLVENT               DMSO
NS                       4
DS                       0
SWH             10135.135 Hz
FIDRES           0.154650 Hz
AQ              3.2331092 sec
RG                    3.15
DW                  49.333 usec
DE                    6.50 usec
TE                   300.5 K
D1              2.00000000 sec
TD0                      1

======== CHANNEL f1 ========
SFO1         500.1344108 MHz
NUC1                    1H
P1                   10.60 usec
PLW1           17.39999962 W

F2 - Processing parameters
SI                  131072
SF           500.1300000 MHz
WDW                     EM
SSB         0
LB                    1.00 Hz
GB          0
PC                    6.00
```

Figure S 20: ^{1}H-NMR Charts for hit 85 (NCI 14040)

17
H1 NMR

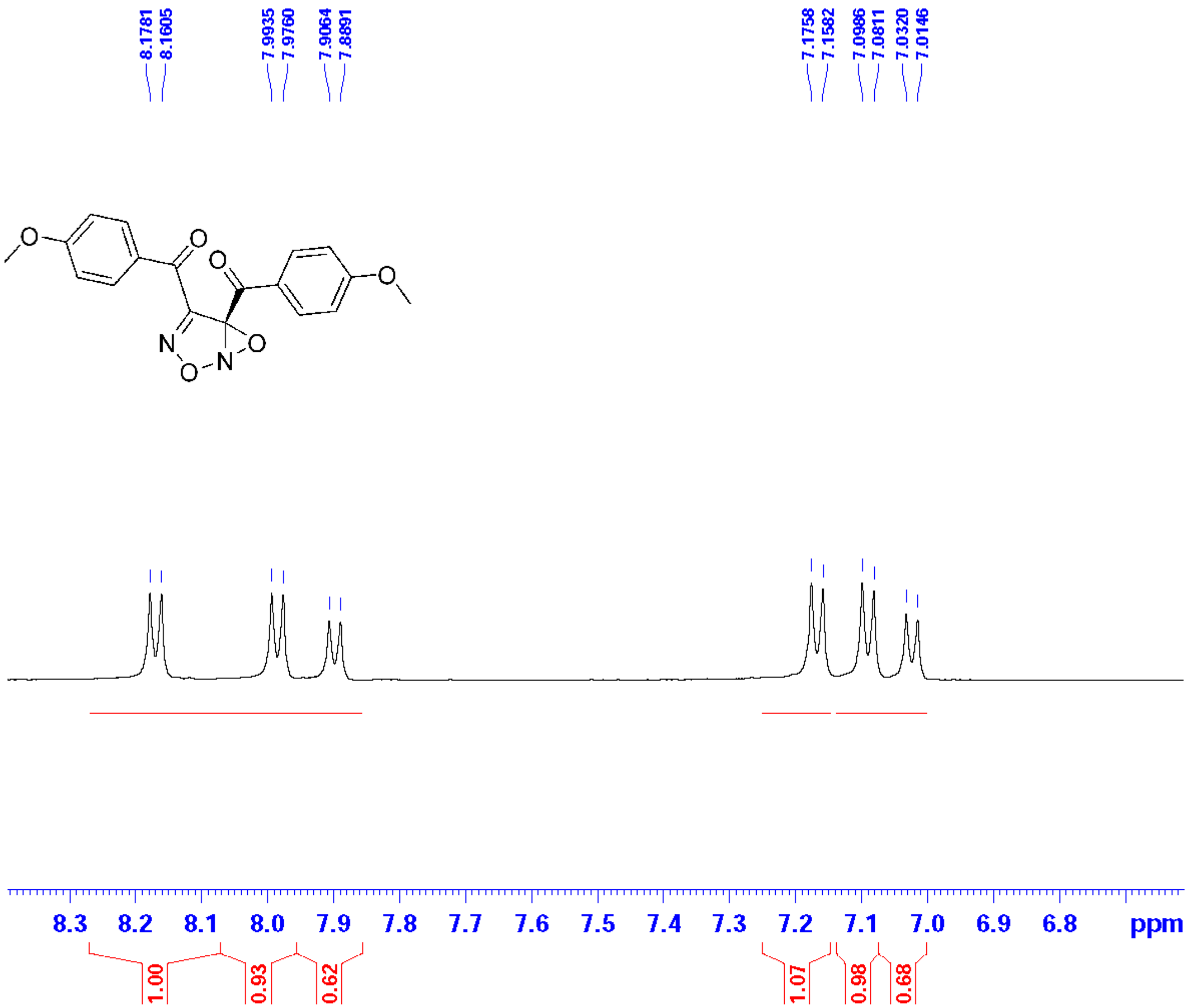

The University of Jordan
Faculty of Science
Department of Chemistry

Instrument Model:
Bruker 500 MHz–Avance III

Operator: Rola Hassouneh
nmr500@ju.edu.jo


```
Current Data Parameters
NAME          16nov15jalal
EXPNO                 1411
PROCNO                   1

F2 - Acquisition Parameters
Date_             20161122
Time                 10.25
INSTRUM              spect
PROBHD    5 mm PABBO BB/
PULPROG                 zg
TD                   65536
SOLVENT               DMSO
NS                       4
DS                       0
SWH              10135.135 Hz
FIDRES            0.154650 Hz
AQ               3.2331092 sec
RG                    3.15
DW                  49.333 usec
DE                    6.50 usec
TE                   300.5 K
D1              2.00000000 sec
TD0                      1

======== CHANNEL f1 ========
SFO1           500.1344108 MHz
NUC1                    1H
P1                   10.60 usec
PLW1           17.39999962 W

F2 - Processing parameters
SI                  131072
SF             500.1300000 MHz
WDW                     EM
SSB       0
LB                    1.00 Hz
GB        0
PC                    6.00
```

Figure S 21: ¹H-NMR Charts for Hit 85 (NCI 14040)

17
H1 NMR

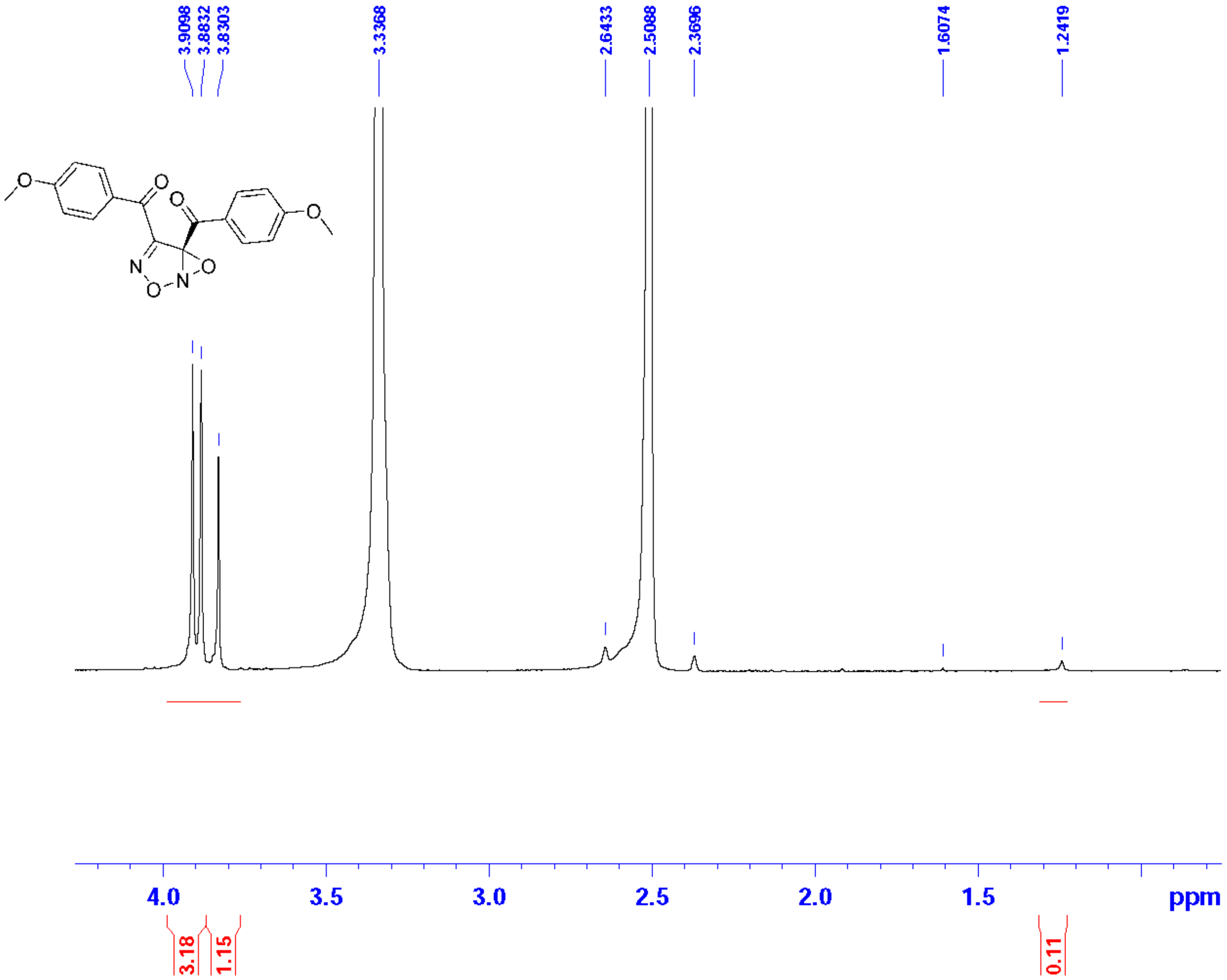

The University of Jordan
Faculty of Science
Department of Chemistry

Instrument Model:
Bruker 500 MHz–Avance III

Operator: Rola Hassouneh
nmr500@ju.edu.jo

Current Data Parameters

NAME	16nov15jalal
EXPNO	1411
PROCNO	1

F2 - Acquisition Parameters

Date_	20161122
Time	10.25
INSTRUM	spect
PROBHD	5 mm PABBO BB/
PULPROG	zg
TD	65536
SOLVENT	DMSO
NS	4
DS	0
SWH	10135.135 Hz
FIDRES	0.154650 Hz
AQ	3.2331092 sec
RG	3.15
DW	49.333 usec
DE	6.50 usec
TE	300.5 K
D1	2.00000000 sec
TD0	1

======== CHANNEL f1 ========

SFO1	500.1344108 MHz
NUC1	1H
P1	10.60 usec
PLW1	17.39999962 W

F2 - Processing parameters

SI	131072
SF	500.1300000 MHz
WDW	EM
SSB	0
LB	1.00 Hz
GB	0
PC	6.00

Figure S 22: ^{13}C-NMR Charts for Hit 85 (NCI 14040)

17
c13

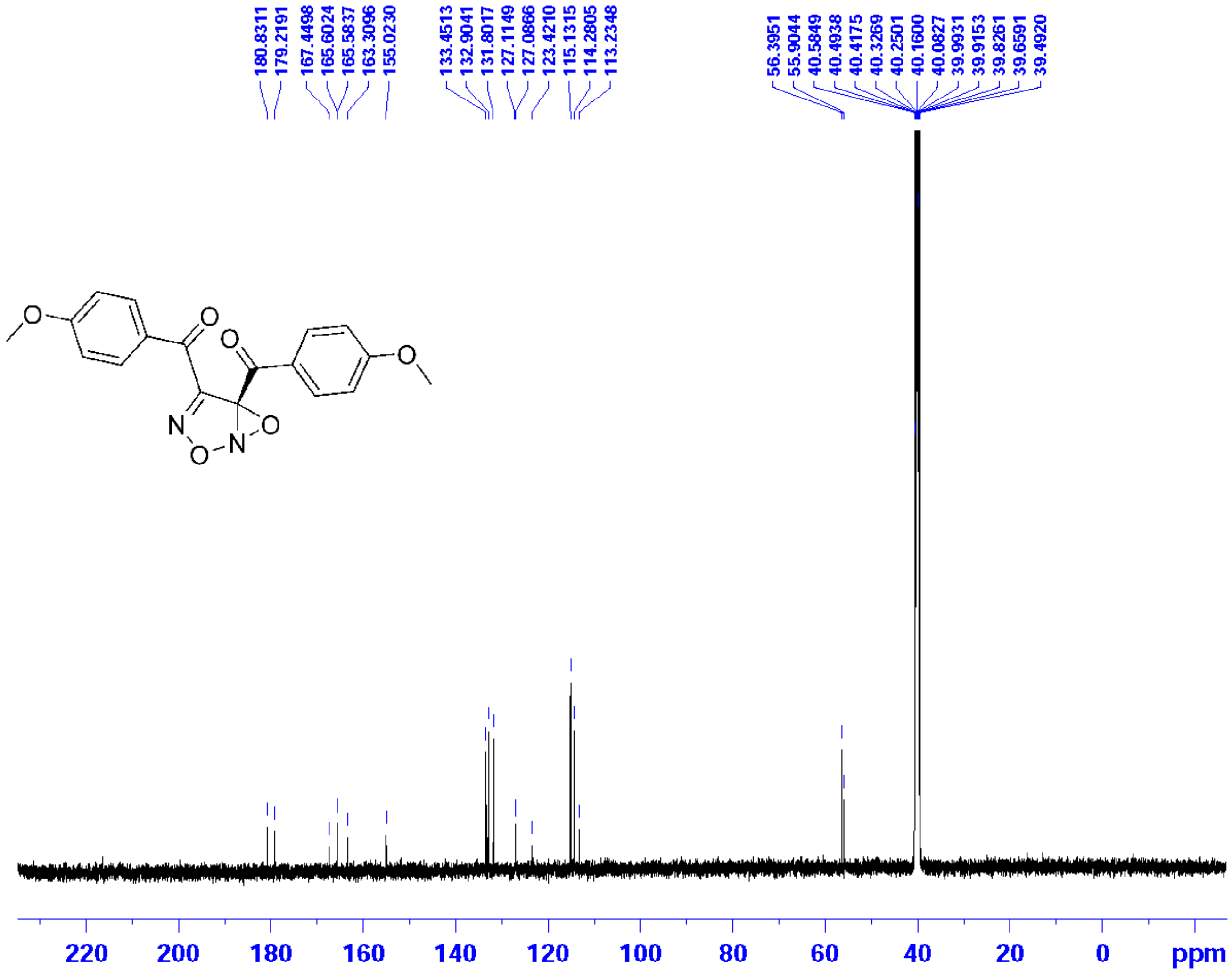

The University of Jordan
Faculty of Science
Department of Chemistry

Instrument Model:
Bruker 500 MHz–Avance III

Operator: Rola Hassouneh
nmr500@ju.edu.jo

Current Data Parameters
NAME 16nov15jalal
EXPNO 1412
PROCNO 1

F2 - Acquisition Parameters
Date_ 20161122
Time 10.25
INSTRUM spect
PROBHD 5 mm PABBO BB/
PULPROG zgpg30
TD 65536
SOLVENT DMSO
NS 1948
DS 4
SWH 32894.738 Hz
FIDRES 0.501934 Hz
AQ 0.9961472 sec
RG 202.06
DW 15.200 usec
DE 6.50 usec
TE 300.6 K
D1 2.00000000 sec
D11 0.03000000 sec
TD0 1

======== CHANNEL f1 ========
SFO1 125.7708561 MHz
NUC1 13C
P1 10.00 usec
PLW1 93.32499695 W

======== CHANNEL f2 ========
SFO2 500.1320005 MHz
NUC2 1H
CPDPRG[2 waltz16
PCPD2 80.00 usec
PLW2 17.39999962 W
PLW12 0.31127000 W
PLW13 0.19921000 W

F2 - Processing parameters
SI 32768
SF 125.7577890 MHz
WDW EM
SSB 0
LB 1.00 Hz
GB 0
PC 1.80

Figure S 23: ^{13}C-NMR Charts for Hit 85 (NCI 14040)

17
c13

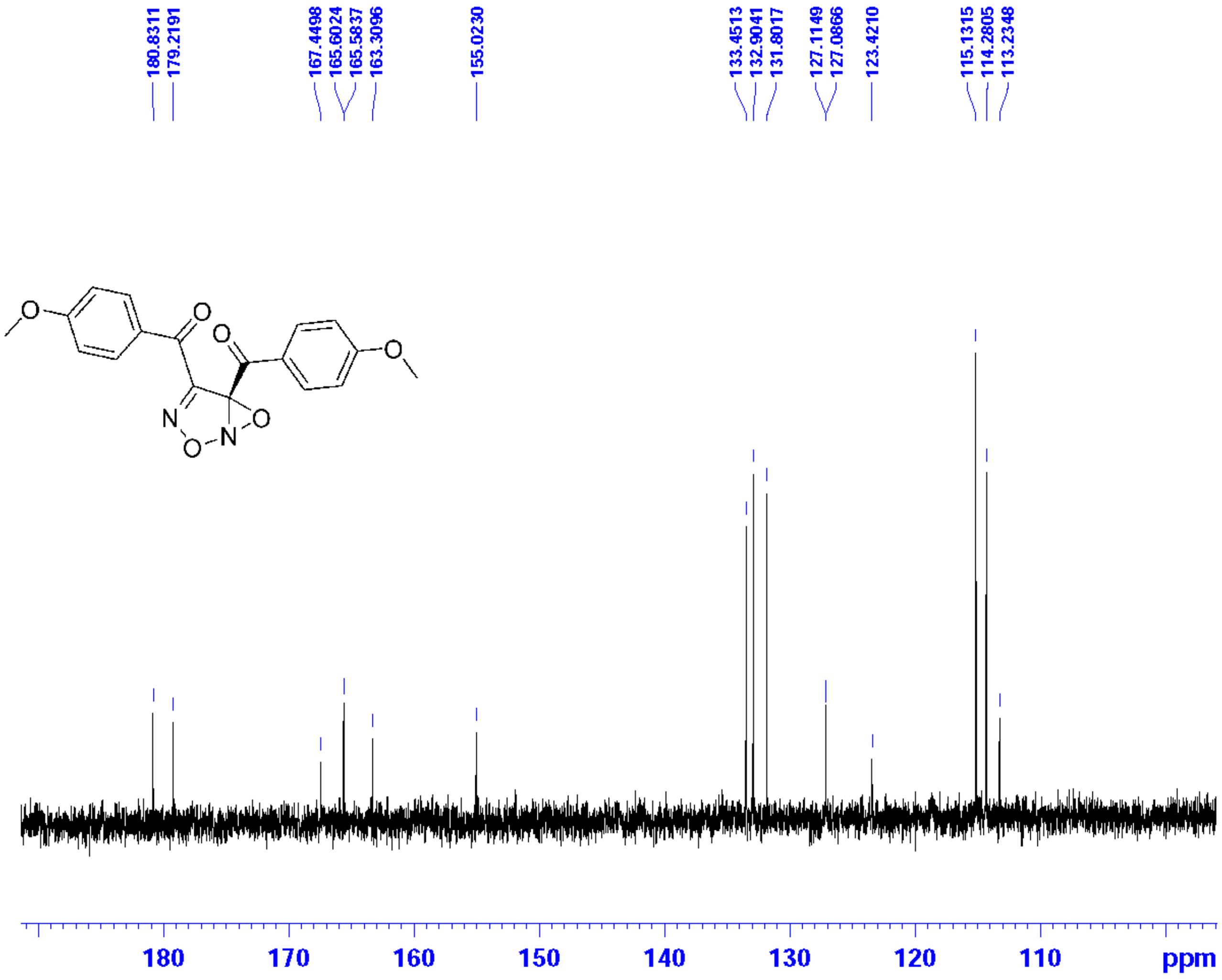

The University of Jordan
Faculty of Science
Department of Chemistry

Instrument Model:
Bruker 500 MHz–Avance III

Operator: Rola Hassouneh
nmr500@ju.edu.jo


```
Current Data Parameters
NAME        16nov15jalal
EXPNO               1412
PROCNO                 1

F2 - Acquisition Parameters
Date_           20161122
Time               10.25
INSTRUM            spect
PROBHD   5 mm PABBO BB/
PULPROG          zgpg30
TD                 65536
SOLVENT             DMSO
NS                  1948
DS                     4
SWH           32894.738 Hz
FIDRES         0.501934 Hz
AQ            0.9961472 sec
RG                202.06
DW                15.200 usec
DE                  6.50 usec
TE                 300.6 K
D1            2.00000000 sec
D11           0.03000000 sec
TD0                    1

======== CHANNEL f1 ========
SFO1         125.7708561 MHz
NUC1                 13C
P1                 10.00 usec
PLW1         93.32499695 W

======== CHANNEL f2 ========
SFO2         500.1320005 MHz
NUC2                  1H
CPDPRG[2          waltz16
PCPD2              80.00 usec
PLW2         17.39999962 W
PLW12         0.31127000 W
PLW13         0.19921000 W

F2 - Processing parameters
SI                 32768
SF           125.7577890 MHz
WDW                   EM
SSB         0
LB                  1.00 Hz
GB          0
PC                  1.80
```

The University of Jordan
Faculty of science
Department of Chemistry

Mass Spectrum Molecular Formula Report

Analysis Info

Analysis Name	F:\Data\2017JUN14\AREEJ_000046.d	Acquisition Date	6/19/2017 8:41:05 AM
Method	ESI-POS-2017	Operator	Bruker_PC
Sample Name	17(14040)	Instrument	apex-IV
Comment	MEOH		

Acquisition Parameter

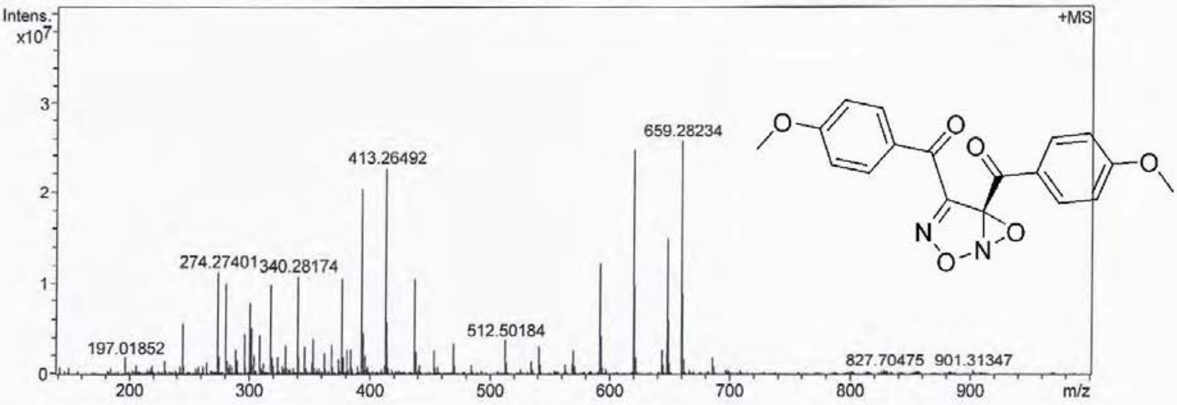

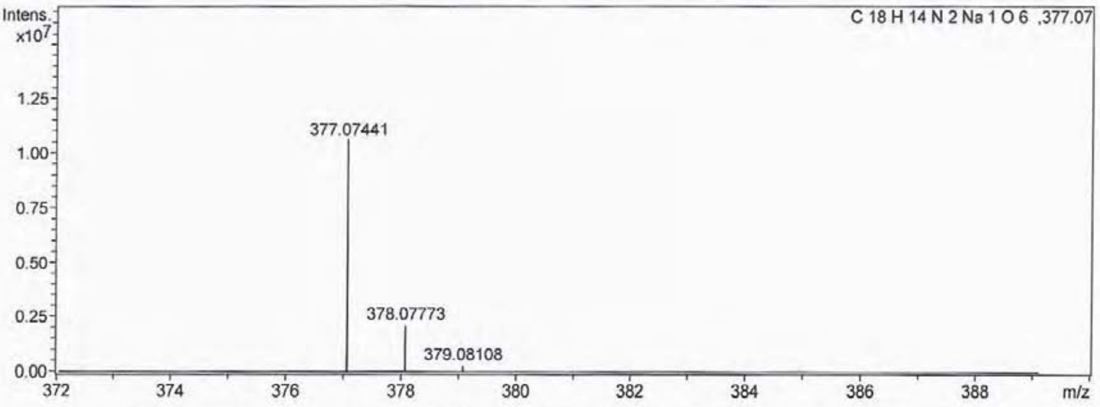

Sum Formula	Sigma	m/z	Err [ppm]	Mean Err [ppm]	Err [mDa]	rdb	N Rule	e⁻
C 18 H 14 N 2 Na 1 O 6	0.020	377.07441	2.12	1.80	0.80	12.50	ok	even

Bruker Daltonics DataAnalysis 3.4 printed: 6/19/2017 8:45:07 AM Page 1 of 1

Figure S 24: Mass Spectrum for Hit 85 (NCI 14040)

Mass Spectrum List Report

Analysis Info

Analysis Name	F:\Data\2017JUN14\AREEJ_000046.d	Acquisition Date	6/19/2017 8:41:05 AM
Method	ESI-POS-2017	Operator	Bruker_PC
Sample Name	17(14040)	Instrument	apex-IV
Comment	MEOH		

Acquisition Parameter

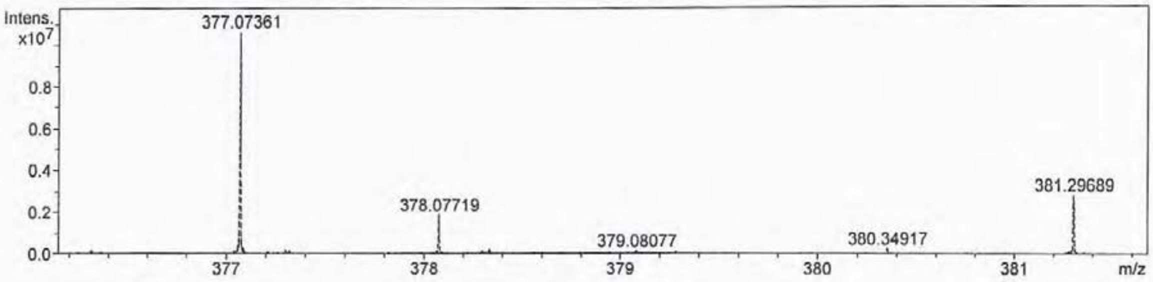

#	m/z	I	I %
1	245.07847	5639665	21.9
2	274.27401	11337571	44.1
3	281.17226	10020214	38.9
4	296.25599	4517782	17.6
5	301.14091	7825119	30.4
6	302.30531	5175217	20.1
7	309.07324	4391450	17.1
8	318.30000	9869178	38.4
9	330.33640	3185209	12.4
10	340.28174	10875133	42.3
11	346.33124	3132938	12.2
12	353.26580	3936987	15.3
13	368.31298	3302057	12.8
14	377.07361	10633389	41.3
15	393.29645	20415654	79.3
16	394.30015	4620799	18.0
17	413.26492	22648875	88.0
18	414.26860	5666709	22.0
19	437.19212	10639115	41.3
20	469.32719	3456702	13.4
21	512.50184	3858115	15.0
22	540.53277	3130015	12.2
23	591.49253	12214447	47.5
24	592.49649	4226130	16.4
25	619.52366	24976187	97.1
26	620.52695	9802814	38.1
27	647.55487	15009656	58.3
28	648.55785	6052829	23.5
29	659.28234	25731827	100.0
30	660.28696	9023160	35.1

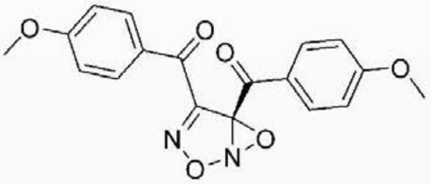

Figure S 25: Mass Spectrum for Hit 85 (NCI 14040)

"NMR" Charts &Mass Spectrum For Hit 112 (NCI 12415)

Figure S 26: ^{1}H-NMR Charts For Hit 112 (NCI 12415)

13
H1 NMR

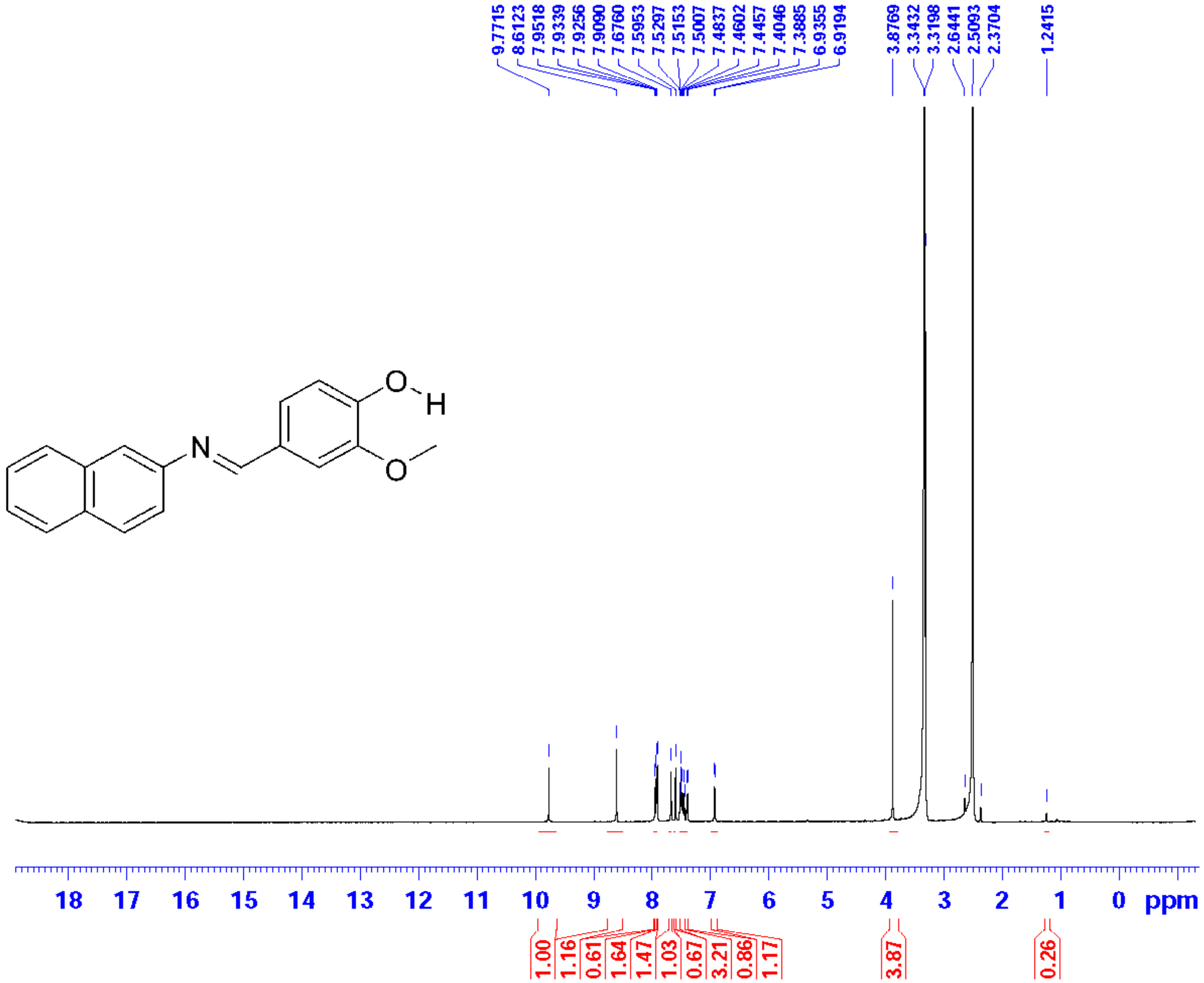

The University of Jordan
Faculty of Science
Department of Chemistry

Instrument Model:
Bruker 500 MHz–Avance III

Operator: Rola Hassouneh
nmr500@ju.edu.jo


```
Current Data Parameters
NAME          16nov15jalal
EXPNO                 1391
PROCNO                   1

F2 - Acquisition Parameters
Date_             20161122
Time                  8.42
INSTRUM              spect
PROBHD     5 mm PABBO BB/
PULPROG                 zg
TD                   65536
SOLVENT               DMSO
NS                       4
DS                       0
SWH              10135.135 Hz
FIDRES            0.154650 Hz
AQ               3.2331092 sec
RG                    3.15
DW                  49.333 usec
DE                    6.50 usec
TE                   300.4 K
D1              2.00000000 sec
TD0                      1

======== CHANNEL f1 ========
SFO1           500.1344108 MHz
NUC1                    1H
P1                   10.60 usec
PLW1           17.39999962 W

F2 - Processing parameters
SI                  131072
SF             500.1300000 MHz
WDW                     EM
SSB          0
LB                    1.00 Hz
GB           0
PC                    2.00
```

Figure S 27: ^{1}H-NMR Charts For Hit 112 (NCI 12415)

13
H1 NMR

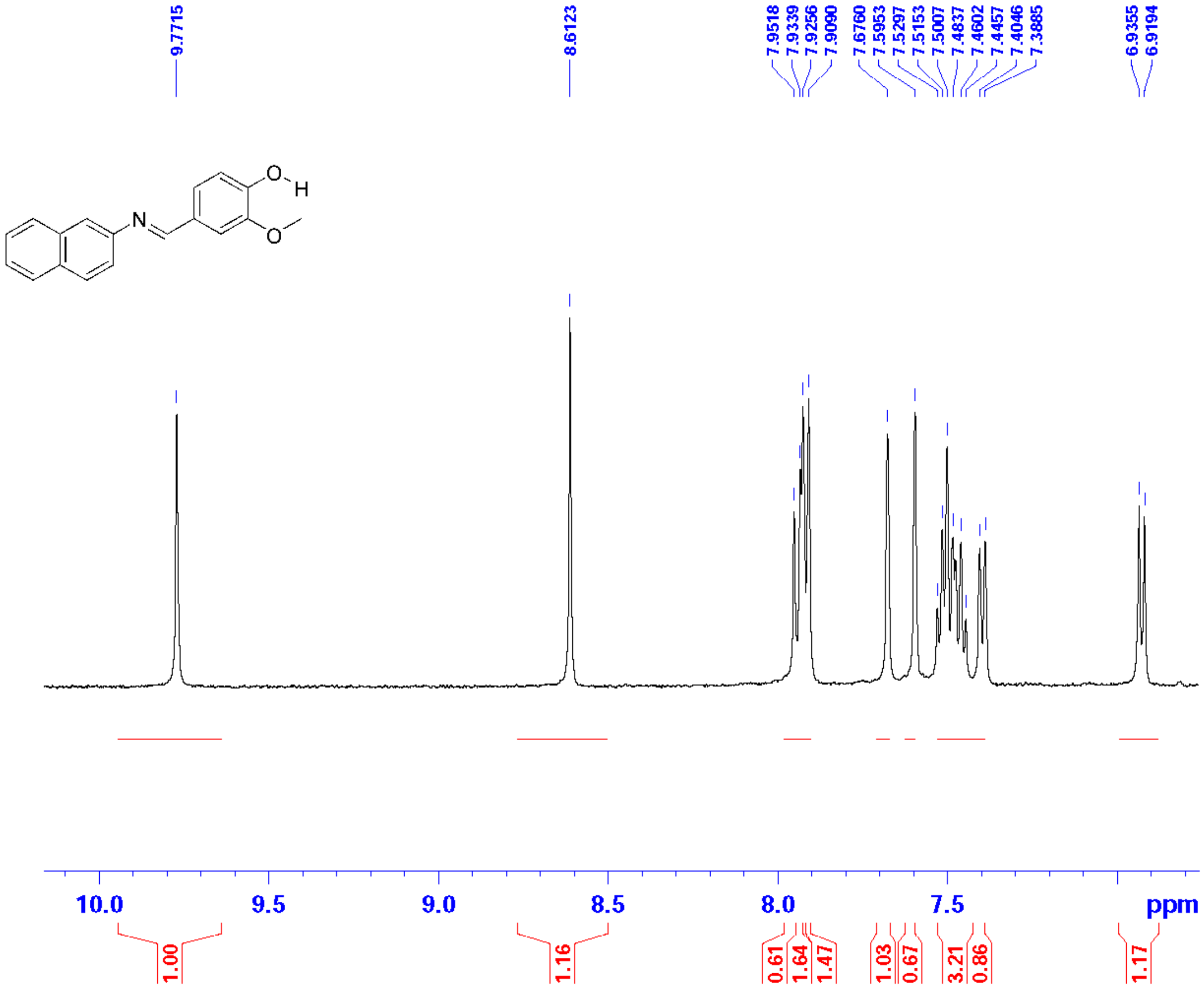

The University of Jordan
Faculty of Science
Department of Chemistry

Instrument Model:
Bruker 500 MHz–Avance III

Operator: Rola Hassouneh
nmr500@ju.edu.jo


```
Current Data Parameters
NAME          16nov15jalal
EXPNO                 1391
PROCNO                   1

F2 - Acquisition Parameters
Date_             20161122
Time                  8.42
INSTRUM              spect
PROBHD     5 mm PABBO BB/
PULPROG                 zg
TD                   65536
SOLVENT               DMSO
NS                       4
DS                       0
SWH             10135.135 Hz
FIDRES           0.154650 Hz
AQ              3.2331092 sec
RG                    3.15
DW                  49.333 usec
DE                    6.50 usec
TE                   300.4 K
D1              2.00000000 sec
TD0                      1

======== CHANNEL f1 ========
SFO1           500.1344108 MHz
NUC1                    1H
P1                   10.60 usec
PLW1           17.39999962 W

F2 - Processing parameters
SI                  131072
SF             500.1300000 MHz
WDW                     EM
SSB      0
LB                    1.00 Hz
GB       0
PC                    2.00
```

Figure S 28: ^{1}H-NMR Charts For Hit 112 (NCI 12415)

13
H1 NMR

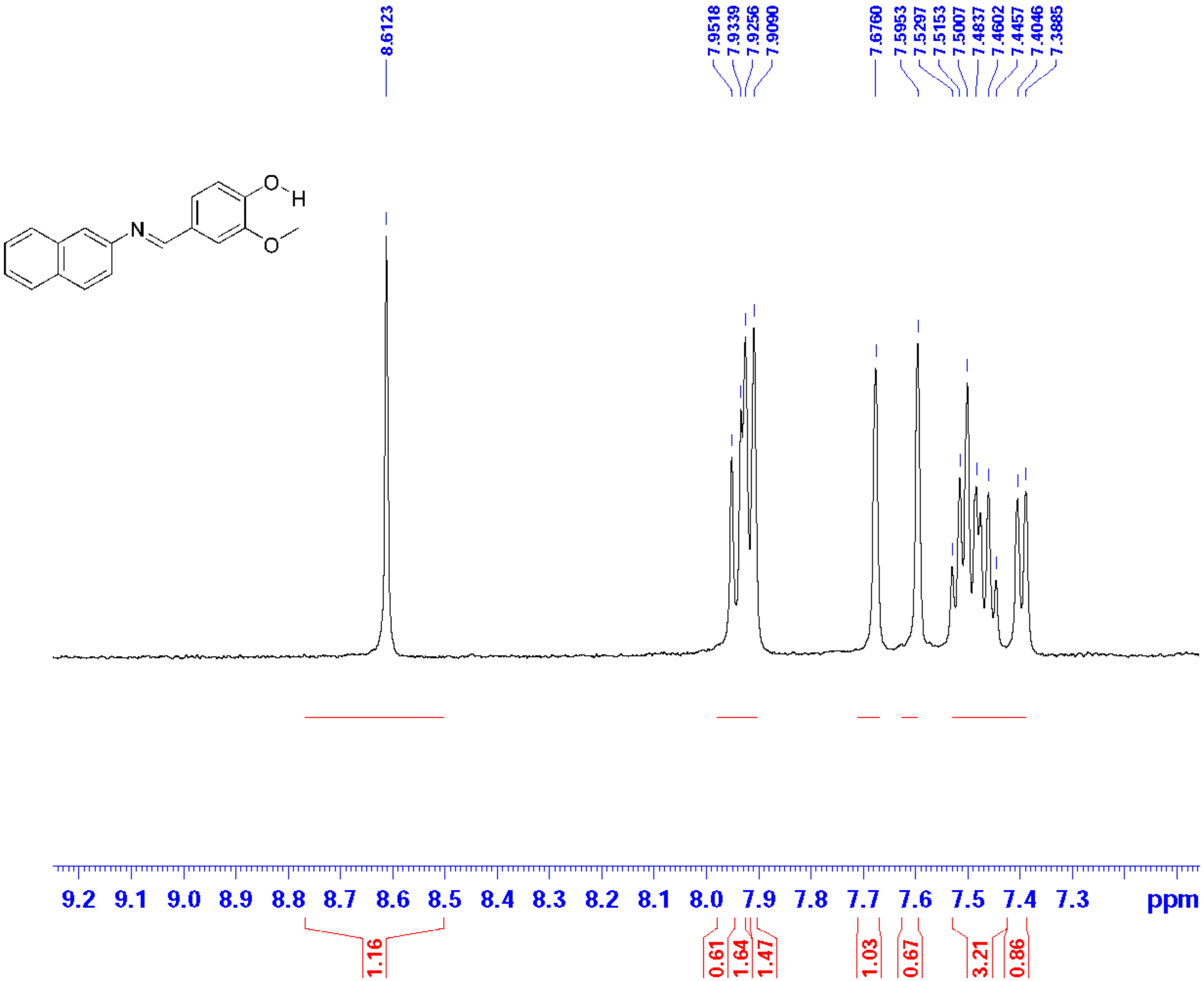

The University of Jordan
Faculty of Science
Department of Chemistry

Instrument Model:
Bruker 500 MHz–Avance III

Operator: Rola Hassouneh
nmr500@ju.edu.jo

```
Current Data Parameters
NAME          16nov15jalal
EXPNO                 1391
PROCNO                   1

F2 - Acquisition Parameters
Date_             20161122
Time                  8.42
INSTRUM              spect
PROBHD    5 mm PABBO BB/
PULPROG                 zg
TD                   65536
SOLVENT               DMSO
NS                       4
DS                       0
SWH            10135.135 Hz
FIDRES          0.154650 Hz
AQ             3.2331092 sec
RG                    3.15
DW                  49.333 usec
DE                    6.50 usec
TE                   300.4 K
D1            2.00000000 sec
TD0                      1

======== CHANNEL f1 ========
SFO1         500.1344108 MHz
NUC1                    1H
P1                   10.60 usec
PLW1           17.39999962 W

F2 - Processing parameters
SI                  131072
SF           500.1300000 MHz
WDW                     EM
SSB       0
LB                    1.00 Hz
GB        0
PC                    2.00
```

13
c13

Figure S 29: ^{13}C-NMR Charts for Hit 112 (NCI 12415)

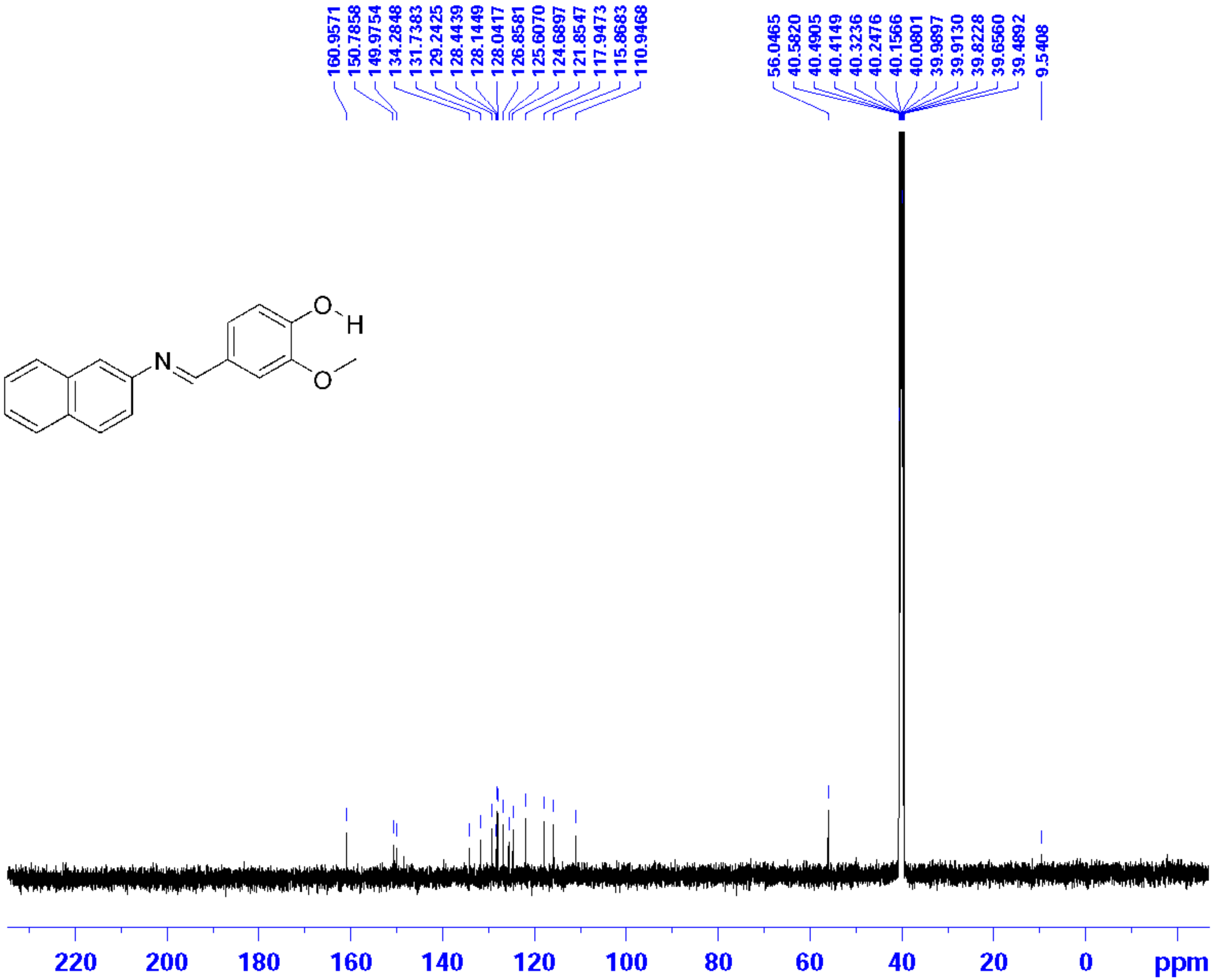

The University of Jordan
Faculty of Science
Department of Chemistry

Instrument Model:
Bruker 500 MHz–Avance III

Operator: Rola Hassouneh
nmr500@ju.edu.jo

```
Current Data Parameters
NAME          16nov15jalal
EXPNO                 1392
PROCNO                   1

F2 - Acquisition Parameters
Date_             20161122
Time                  9.43
INSTRUM              spect
PROBHD     5 mm PABBO BB/
PULPROG            zgpg30
TD                   65536
SOLVENT               DMSO
NS                    1176
DS                       4
SWH              32894.738 Hz
FIDRES            0.501934 Hz
AQ               0.9961472 sec
RG                  202.06
DW                  15.200 usec
DE                    6.50 usec
TE                   300.5 K
D1              2.00000000 sec
D11             0.03000000 sec
TD0                      1

======== CHANNEL f1 ========
SFO1           125.7708561 MHz
NUC1                   13C
P1                   10.00 usec
PLW1           93.32499695 W

======== CHANNEL f2 ========
SFO2           500.1320005 MHz
NUC2                    1H
CPDPRG[2           waltz16
PCPD2                80.00 usec
PLW2           17.39999962 W
PLW12           0.31127000 W
PLW13           0.19921000 W

F2 - Processing parameters
SI                   32768
SF             125.7577890 MHz
WDW                     EM
SSB      0
LB                    1.00 Hz
GB       0
PC                    1.20
```

Figure S 30: ^{13}C-NMR Charts for Hit 112 (NCI 12415)

13
c13

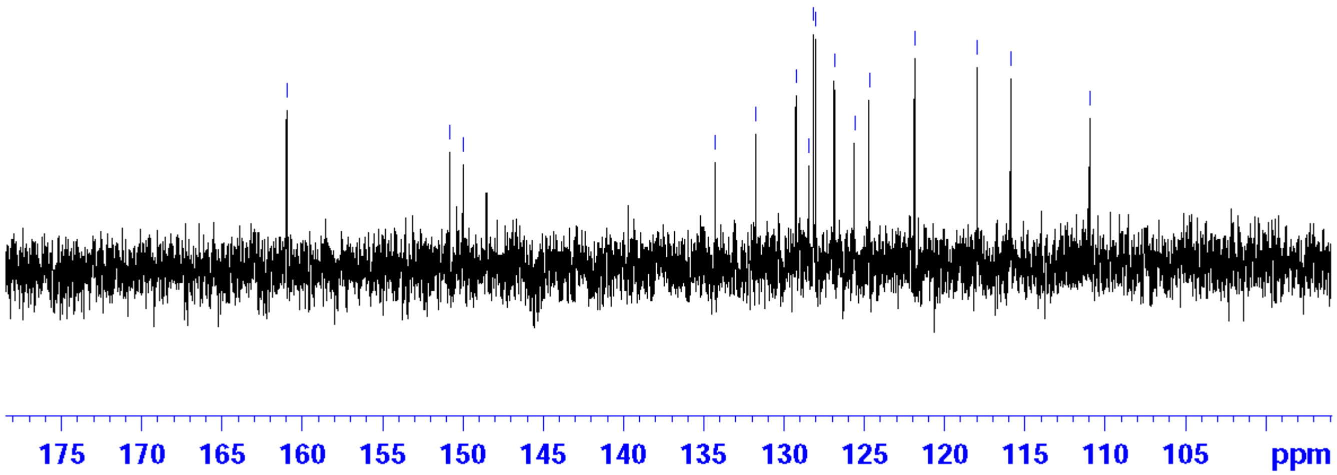

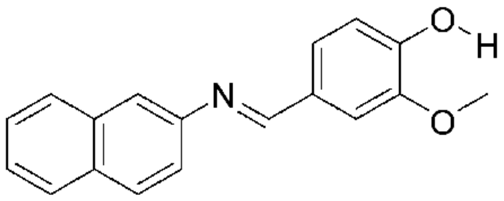

The University of Jordan
Faculty of Science
Department of Chemistry

Instrument Model:
Bruker 500 MHz–Avance III

Operator: Rola Hassouneh
nmr500@ju.edu.jo


```
Current Data Parameters
NAME          16nov15jalal
EXPNO                 1392
PROCNO                   1

F2 - Acquisition Parameters
Date_             20161122
Time                  9.43
INSTRUM              spect
PROBHD   5 mm PABBO BB/
PULPROG            zgpg30
TD                   65536
SOLVENT               DMSO
NS                    1176
DS                       4
SWH             32894.738 Hz
FIDRES           0.501934 Hz
AQ              0.9961472 sec
RG                  202.06
DW                  15.200 usec
DE                    6.50 usec
TE                   300.5 K
D1              2.00000000 sec
D11             0.03000000 sec
TD0                      1

======== CHANNEL f1 ========
SFO1          125.7708561 MHz
NUC1                   13C
P1                   10.00 usec
PLW1           93.32499695 W

======== CHANNEL f2 ========
SFO2          500.1320005 MHz
NUC2                    1H
CPDPRG[2            waltz16
PCPD2                80.00 usec
PLW2           17.39999962 W
PLW12           0.31127000 W
PLW13           0.19921000 W

F2 - Processing parameters
SI                   32768
SF            125.7577890 MHz
WDW                     EM
SSB      0
LB                    1.00 Hz
GB       0
PC                    1.20
```

The University of Jordan
Faculty of science
Department of Chemistry

Mass Spectrum Molecular Formula Report

Analysis Info

Analysis Name	F:\Data\2017JUN14\AREEJ_000044.d	Acquisition Date	6/19/2017 8:13:43 AM
Method	ESI-POS-2017	Operator	Bruker_PC
Sample Name	13(12415)	Instrument	apex-IV
Comment	MEOH		

Acquisition Parameter

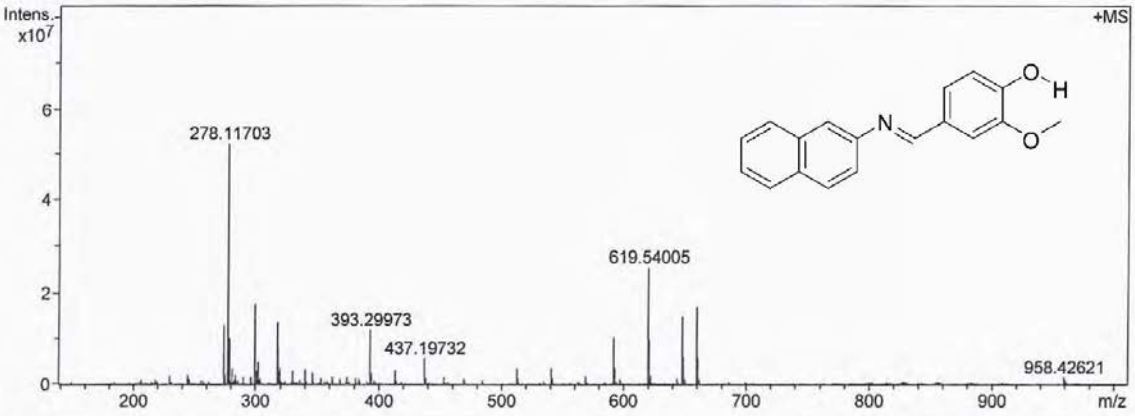

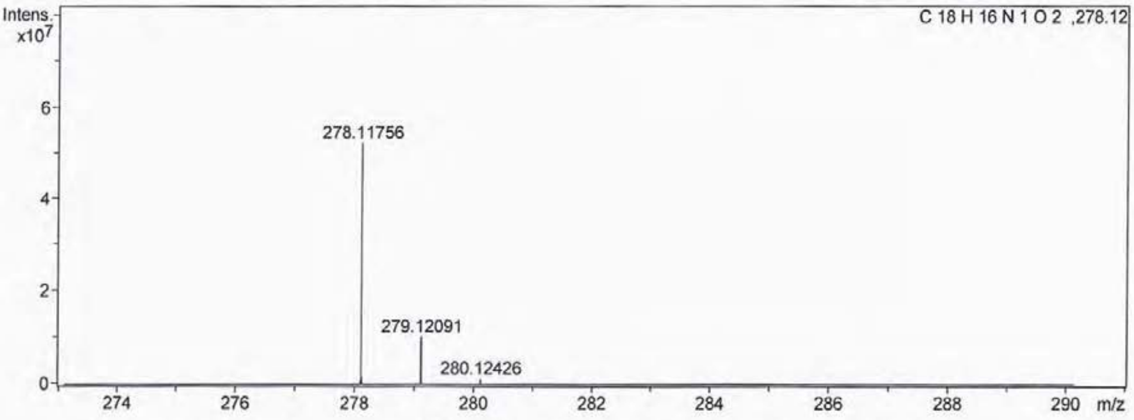

Sum Formula	Sigma	m/z	Err [ppm]	Mean Err [ppm]	Err [mDa]	rdb	N Rule	e⁻
C 18 H 16 N 1 O 2	0.005	278.11756	1.90	1.71	0.53	11.50	ok	even

Figure S 31: Mass Spectrum for Hit 112 (NCI 12415)

Mass Spectrum List Report

Analysis Info | Acquisition Date 6/19/2017 8:13:43 AM

Analysis Name F:\Data\2017JUN14\AREEJ_000044.d
Method ESI-POS-2017 | Operator Bruker_PC
Sample Name 13(12415) | Instrument apex-IV
Comment MEOH

Acquisition Parameter

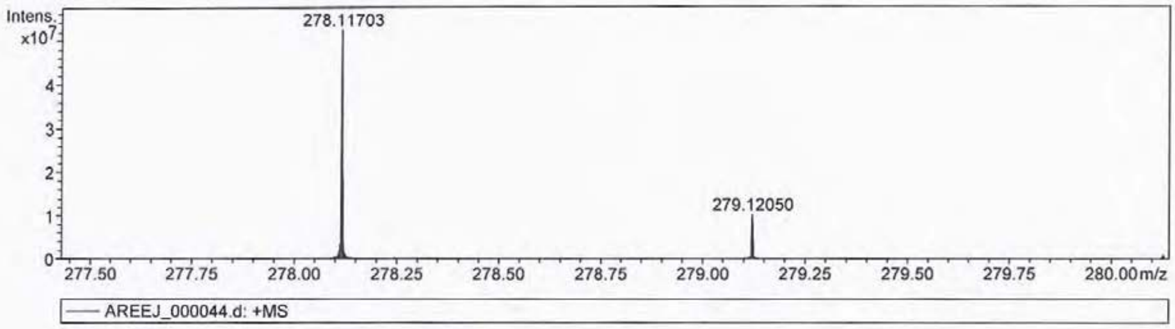

#	m/z	I	I %
1	274.27353	13089400	25.0
2	278.11301	3247748	6.2
3	278.11703	52310148	100.0
4	278.12103	3790884	7.2
5	279.12050	10282288	19.7
6	281.17204	3482016	6.7
7	300.09927	17608025	33.7
8	301.10283	3074047	5.9
9	302.30533	5162407	9.9
10	318.19658	13839597	26.5
11	318.24071	2955174	5.6
12	318.30050	8358222	16.0
13	319.20021	2848026	5.4
14	320.25634	3579169	6.8
15	330.33730	3019740	5.8
16	340.28308	3445061	6.6
17	346.33259	2677810	5.1
18	393.29973	12128411	23.2
19	413.26926	3149420	6.0
20	437.19732	5905134	11.3
21	512.51095	3635453	6.9
22	540.54358	3753113	7.2
23	591.50687	10495684	20.1
24	592.51075	3778459	7.2
25	619.54005	25534862	48.8
26	620.54347	10042321	19.2
27	647.57358	14845881	28.4
28	648.57671	6079226	11.6
29	659.30261	17119038	32.7
30	660.30686	5914942	11.3

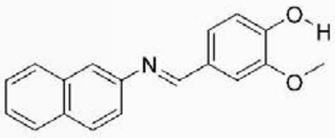

Figure S 32: Mass Spectrum for Hit 112 (NCI 12415)

"NMR" Charts &Mass Spectrum For Hit 141 (NCI 12492)

Figure S 33: ^{1}H-NMR Charts For Hit 141 (NCI 12492)

14
H1 NMR

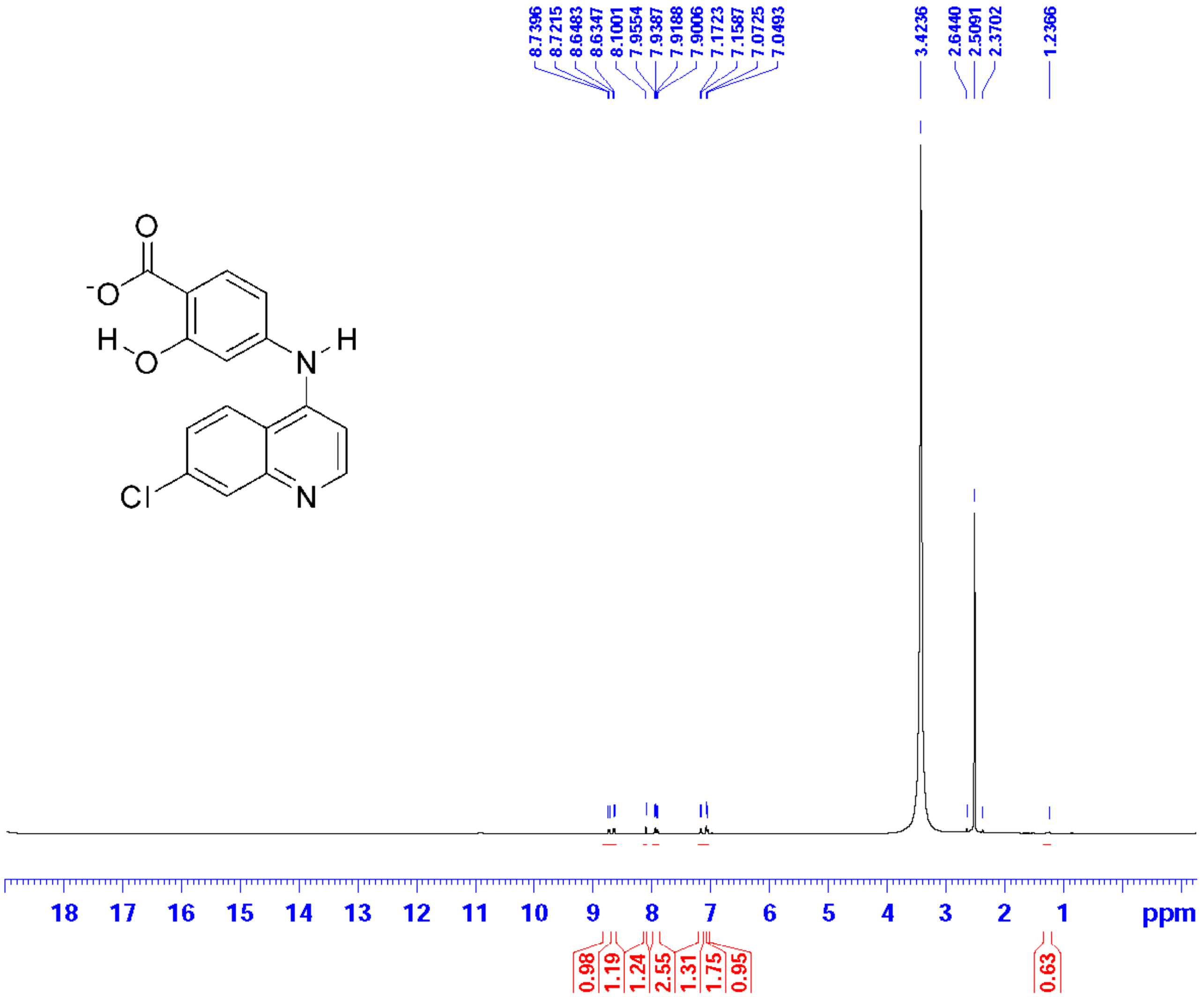

The University of Jordan
Faculty of Science
Department of Chemistry

Instrument Model:
Bruker 500 MHz–Avance III

Operator: Rola Hassouneh
nmr500@ju.edu.jo


```
Current Data Parameters
NAME          16nov15jalal
EXPNO                 1421
PROCNO                   1

F2 - Acquisition Parameters
Date_             20161122
Time                 16.37
INSTRUM              spect
PROBHD     5 mm PABBO BB/
PULPROG                 zg
TD                   65536
SOLVENT               DMSO
NS                     104
DS                       0
SWH              10135.135 Hz
FIDRES            0.154650 Hz
AQ               3.2331092 sec
RG                    3.15
DW                  49.333 usec
DE                    6.50 usec
TE                   300.4 K
D1              2.00000000 sec
TD0                      1

======== CHANNEL f1 ========
SFO1           500.1344108 MHz
NUC1                    1H
P1                   10.60 usec
PLW1           17.39999962 W

F2 - Processing parameters
SI                  131072
SF             500.1300000 MHz
WDW                     EM
SSB          0
LB                    1.00 Hz
GB           0
PC                    1.00
```

Figure S 34: ^{1}H-NMR Charts For Hit 141 (NCI 12492)

14
H1 NMR

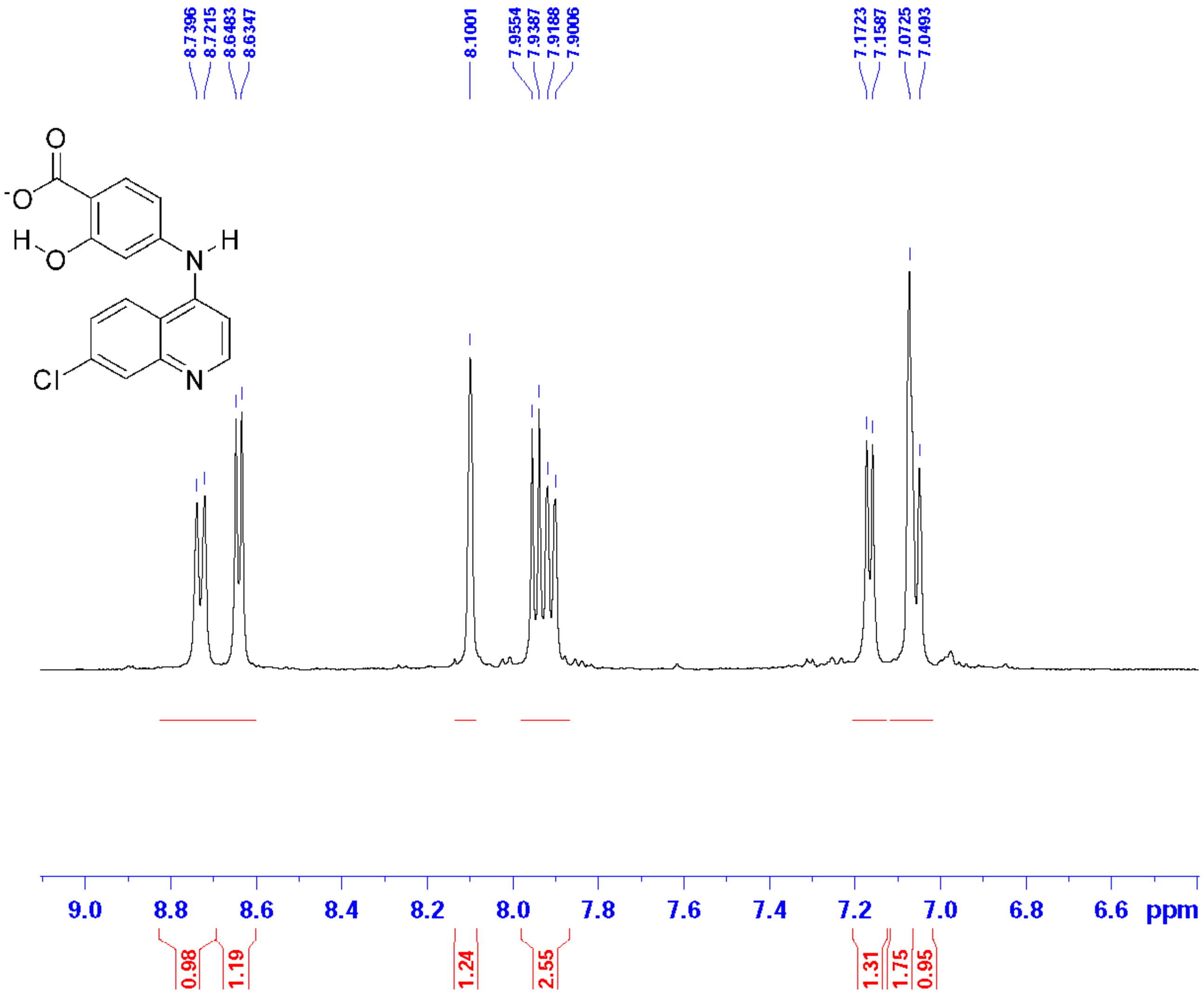

The University of Jordan
Faculty of Science
Department of Chemistry

Instrument Model:
Bruker 500 MHz–Avance III

Operator: Rola Hassouneh
nmr500@ju.edu.jo


```
Current Data Parameters
NAME          16nov15jalal
EXPNO                 1421
PROCNO                   1

F2 - Acquisition Parameters
Date_             20161122
Time                 16.37
INSTRUM              spect
PROBHD    5 mm PABBO BB/
PULPROG                 zg
TD                   65536
SOLVENT               DMSO
NS                     104
DS                       0
SWH             10135.135 Hz
FIDRES           0.154650 Hz
AQ              3.2331092 sec
RG                    3.15
DW                  49.333 usec
DE                    6.50 usec
TE                   300.4 K
D1             2.00000000 sec
TD0                      1

======== CHANNEL f1 ========
SFO1          500.1344108 MHz
NUC1                    1H
P1                   10.60 usec
PLW1           17.39999962 W

F2 - Processing parameters
SI                  131072
SF            500.1300000 MHz
WDW                     EM
SSB       0
LB                    1.00 Hz
GB        0
PC                    1.00
```

Figure S 35: ^{13}C-NMR Charts for Hit 141 (NCI 12492)

14
c13

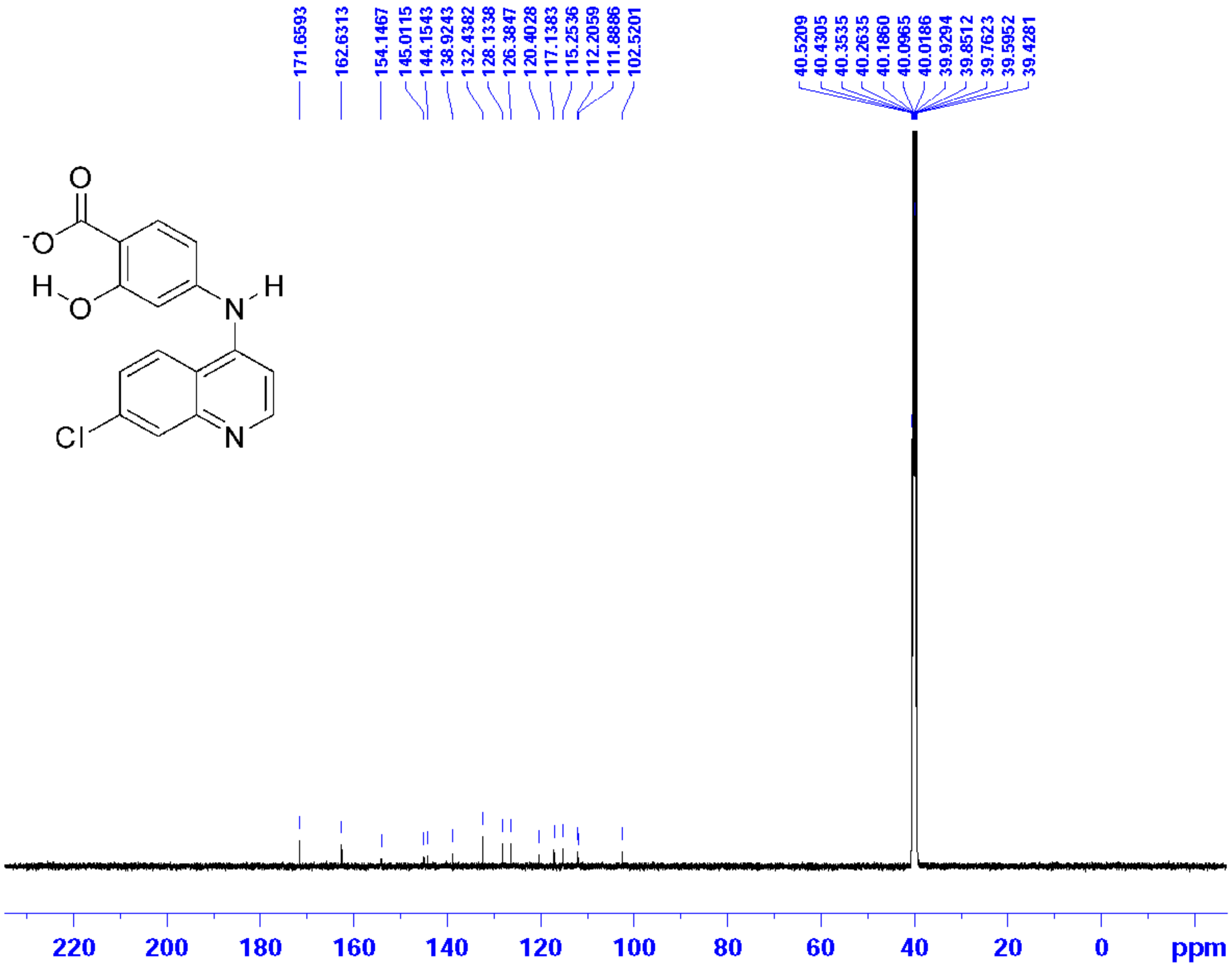

The University of Jordan
Faculty of Science
Department of Chemistry

Instrument Model:
Bruker 500 MHz–Avance III

Operator: Rola Hassouneh
nmr500@ju.edu.jo

```
Current Data Parameters
NAME          16nov15jalal
EXPNO                 1422
PROCNO                   1

F2 - Acquisition Parameters
Date_             20161123
Time                  3.56
INSTRUM              spect
PROBHD     5 mm PABBO BB/
PULPROG            zgpg30
TD                   65536
SOLVENT               DMSO
NS                   13966
DS                       4
SWH             32894.738 Hz
FIDRES           0.501934 Hz
AQ              0.9961472 sec
RG                  202.06
DW                  15.200 usec
DE                    6.50 usec
TE                   300.5 K
D1              2.00000000 sec
D11             0.03000000 sec
TD0                      1

======== CHANNEL f1 ========
SFO1         125.7708561 MHz
NUC1                  13C
P1                  10.00 usec
PLW1          93.32499695 W

======== CHANNEL f2 ========
SFO2         500.1320005 MHz
NUC2                   1H
CPDPRG[2           waltz16
PCPD2               80.00 usec
PLW2          17.39999962 W
PLW12          0.31127000 W
PLW13          0.19921000 W

F2 - Processing parameters
SI                  32768
SF            125.7577890 MHz
WDW                    EM
SSB       0
LB                   1.00 Hz
GB        0
PC                   1.20
```

Figure S 36: ^{13}C-NMR Charts for Hit 141 (NCI 12492)

14
c13

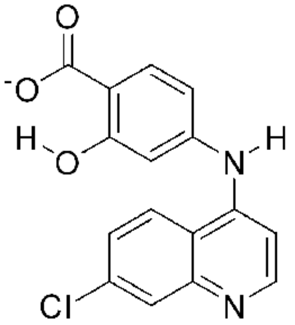

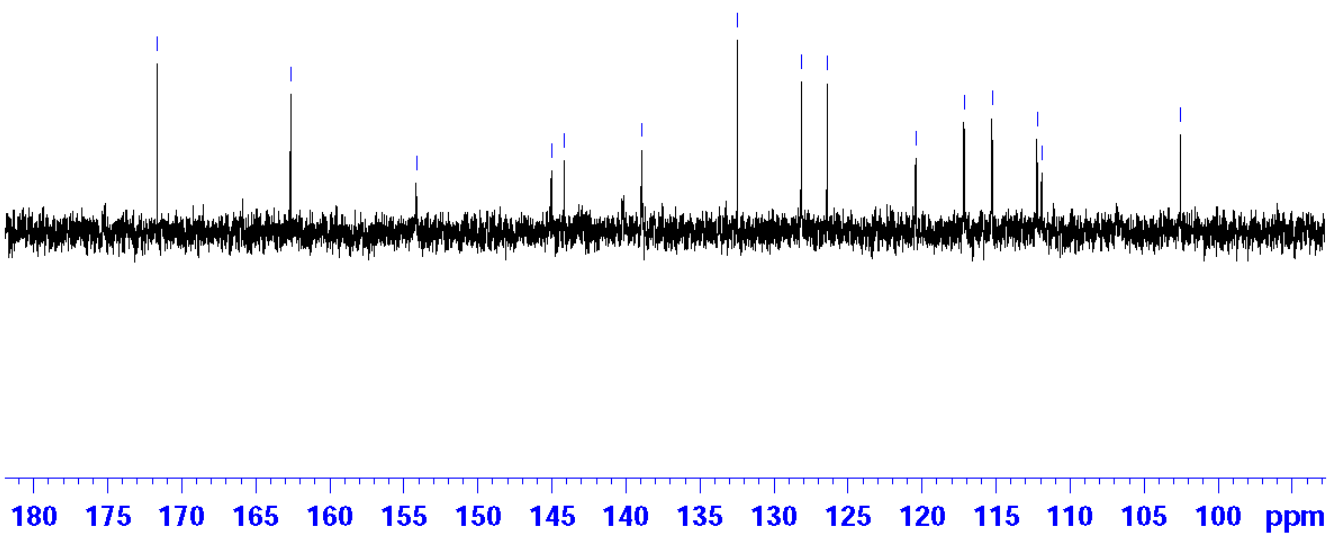

The University of Jordan
Faculty of Science
Department of Chemistry

Instrument Model:
Bruker 500 MHz–Avance III

Operator: Rola Hassouneh
nmr500@ju.edu.jo


```
Current Data Parameters
NAME          16nov15jalal
EXPNO                 1422
PROCNO                   1

F2 - Acquisition Parameters
Date_             20161123
Time                  3.56
INSTRUM              spect
PROBHD   5 mm PABBO BB/
PULPROG            zgpg30
TD                   65536
SOLVENT               DMSO
NS                   13966
DS                       4
SWH              32894.738 Hz
FIDRES            0.501934 Hz
AQ               0.9961472 sec
RG                  202.06
DW                  15.200 usec
DE                    6.50 usec
TE                   300.5 K
D1              2.00000000 sec
D11             0.03000000 sec
TD0                      1

======== CHANNEL f1 ========
SFO1           125.7708561 MHz
NUC1                   13C
P1                   10.00 usec
PLW1           93.32499695 W

======== CHANNEL f2 ========
SFO2           500.1320005 MHz
NUC2                    1H
CPDPRG[2           waltz16
PCPD2                80.00 usec
PLW2           17.39999962 W
PLW12           0.31127000 W
PLW13           0.19921000 W

F2 - Processing parameters
SI                   32768
SF             125.7577890 MHz
WDW                     EM
SSB      0
LB                    1.00 Hz
GB       0
PC                    1.20
```

The University of Jordan
Faculty of science
Department of Chemistry

Mass Spectrum Molecular Formula Report

Analysis Info

Analysis Name	F:\Data\2017JUN14\AREEJ_000045.d	Acquisition Date	6/19/2017 8:20:38 AM
Method	ESI-POS-2017	Operator	Bruker_PC
Sample Name	14(12492)	Instrument	apex-IV
Comment	MEOH		

Acquisition Parameter

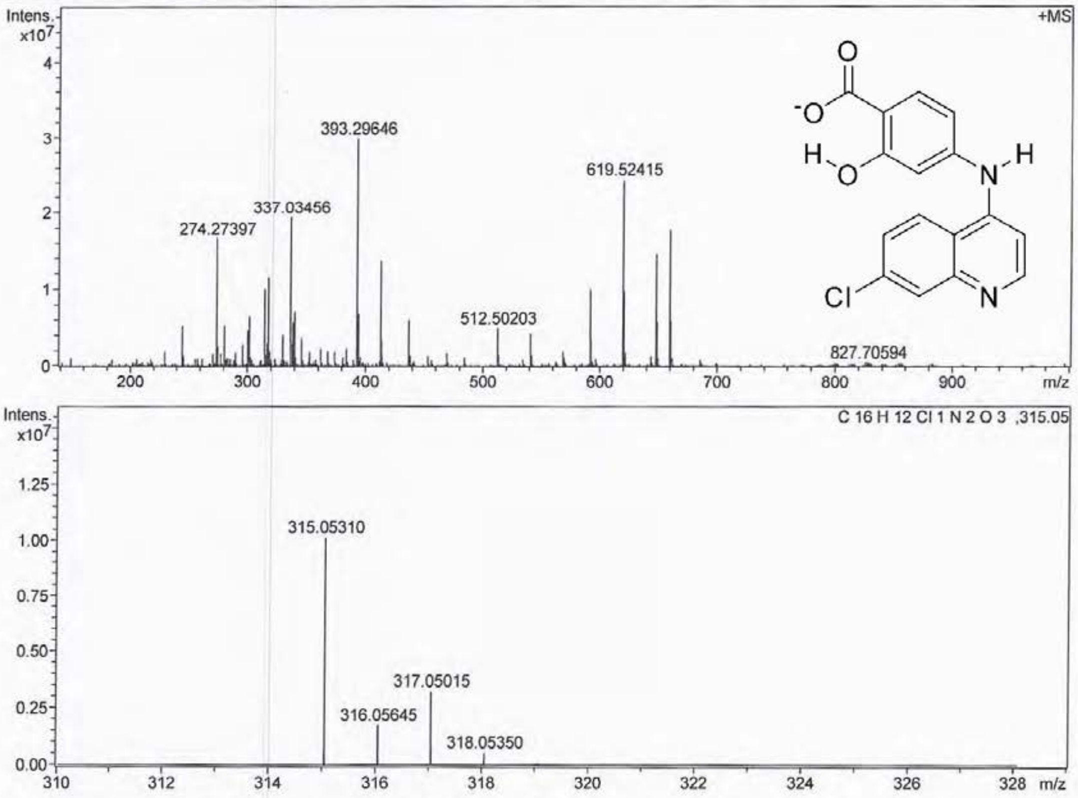

Sum Formula	Sigma	m/z	Err [ppm]	Mean Err [ppm]	Err [mDa]	rdb	N Rule	e⁻
C 16 H 12 Cl 1 N 2 O 3	0.025	315.05310	0.72	0.81	0.23	11.50	ok	even

Figure S 37: Mass Spectrum for Hit 141 (NCI 12492)

Mass Spectrum List Report

Analysis Info

Analysis Name	F:\Data\2017JUN14\AREEJ_000045.d	Acquisition Date	6/19/2017 8:20:38 AM
Method	ESI-POS-2017	Operator	Bruker_PC
Sample Name	14(12492)	Instrument	apex-IV
Comment	MEOH		

Acquisition Parameter

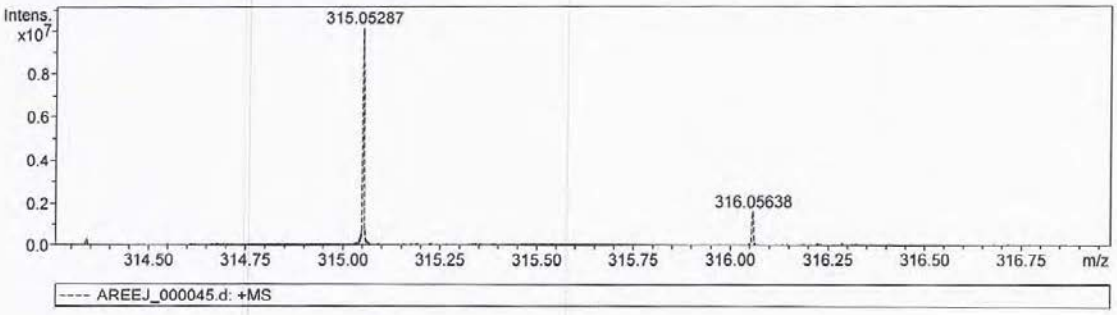

#	m/z	I	I %
1	245.07844	5309069	17.7
2	274.27397	16732708	55.9
3	281.17235	5340670	17.8
4	296.25602	2869358	9.6
5	301.14099	4842422	16.2
6	302.30529	6505375	21.7
7	315.05287	10151806	33.9
8	317.05008	3036703	10.1
9	318.30001	11539904	38.5
10	330.33643	4212503	14.1
11	337.03456	19482162	65.1
12	338.03814	3049525	10.2
13	339.03183	5884984	19.7
14	340.28190	7175028	24.0
15	346.33127	3798818	12.7
16	393.29646	29935589	100.0
17	394.30017	6921065	23.1
18	413.26503	13876873	46.4
19	414.26868	3310086	11.1
20	437.19233	6072152	20.3
21	512.50203	5091468	17.0
22	540.53303	4434711	14.8
23	591.49299	9972464	33.3
24	592.49687	3496731	11.7
25	619.52415	24531609	81.9
26	620.52742	9713245	32.4
27	647.55547	14607720	48.8
28	648.55843	5912165	19.7
29	659.28341	17808765	59.5
30	660.28779	6126105	20.5

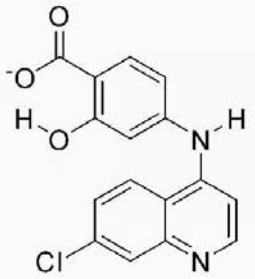

Figure S 38: Mass Spectrum for Hit 141 (NCI 12492)
